# The TPR2 corepressor forms condensates with repressors to fine-tune growth and development in rice

**DOI:** 10.1038/s44318-026-00852-7

**Published:** 2026-07-06

**Authors:** Yu Zhang, Bingchen Li, Yu Chen, Jiawei Li, Yao Wang, Tiantian Ye, Ying Ye, Zhiwei Liu, Zhiyue Feng, Stephanie Hutin, Shaoli Zhou, Jianping Guo, Ruihui Zhang, Zhu Liu, Faming Dong, Haiyan Xiong, Chloe Zubieta, Lizhong Xiong, Xuelei Lai

**Affiliations:** 1 National Key Laboratory of Crop Genetic Improvement, Hubei Hongshan Laboratory, Huazhong Agricultural University, Wuhan, China; 2 Laboratoire de Physiologie Cellulaire et Végétale, Université Grenoble-Alpes, CNRS, CEA, INRAE, IRIG-DBSCI, 17 rue des Martyrs, 38000 Grenoble, France

**Keywords:** Chromatin, Transcription & Genomics, Plant Biology

## Abstract

Transcriptional repression is a central mechanism regulating plant growth and development, yet how plant-specific corepressors achieve robust gene silencing remains unclear. Here, we identify the rice corepressor TPR2 (TOPLESS-RELATED 2) as a phase-separating protein that forms nuclear condensates through the cooperative action of an intrinsically disordered region (IDR1) and a plant-specific N-terminal tetramerization domain. Structure-guided mutagenesis disrupting tetramerization or deleting IDR1 markedly impaired condensate formation in vivo and in vitro. Complementation assays showed that only full-length TPR2, capable of robust condensate formation, rescued the growth defects of the *tpr2* mutant, whereas phase separation-deficient variants failed to do so. Mechanistically, we demonstrate that TPR2 co-condenses with repressors such as D53 and IAA3 to facilitate histone deacetylation and establish repressive chromatin states in a phase separation-dependent manner, thereby fine-tuning key developmental genes. Together, these findings define a condensate-based mechanism for transcriptional repression in plants, linking corepressor phase separation to chromatin modification, gene silencing and developmental control.

## Synopsis

Transcriptional corepressors coordinate chromatin-based gene silencing during plant growth and development, but how their activity is spatially organized remains poorly understood. This study shows that the rice corepressor TPR2 forms biomolecular condensates with transcriptional repressors to promote histone deacetylation, gene repression and normal plant development.

- TPR2 forms dynamic nuclear condensates through the cooperative action of IDR1 and a plant-specific tetramerization domain.
- Condensation-defective TPR2 variants fail to restore normal growth and development in *tpr2* plants.
- TPR2 co-condenses with repressors D53 and IAA3 to promote hypoacetylated, transcriptionally repressive chromatin states.
- TPR2 phase separation links corepressor assembly to chromatin modification, gene repression, and developmental control in rice.

## Abstract (web summary)

TPR2 condensates recruit transcriptional repressors to establish hypoacetylated chromatin states that coordinate gene expression and rice development.

## Graphical abstract

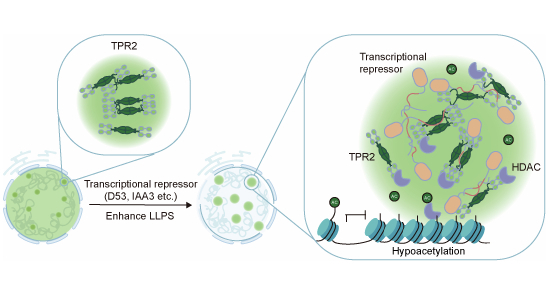

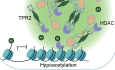

## Introduction

Transcriptional repression is a central mechanism regulating plant growth, development, and responses to environmental cues (Gao et al, 2023; Gouran and Brady, 2024; Jung et al, 2020; Li et al, 2025; Liu et al, 2014; Plant et al, 2021; Silva et al, 2020; Zhu et al, 2021). Recent studies suggest that the formation of biomolecular condensates through liquid-liquid phase separation (LLPS) provides a general mechanism for organizing cellular processes, including transcriptional regulation (Banani et al, 2017; Emenecker et al, 2020; Hyman et al, 2014; Su et al, 2021). LLPS can concentrate specific proteins and nucleic acids into dynamic compartments without membrane boundaries, thereby facilitating efficient biochemical reactions (Banani et al, 2017; López-Palacios and Andersen, 2023; Wang et al, 2024; Zhang et al, 2021). In gene regulation, LLPS is increasingly recognized as a mechanism for assembling transcriptional machinery at precise genomic loci (Gibson et al, 2019; Hnisz et al, 2017; Hu et al, 2024). While the role of LLPS in transcriptional activation is well established—exemplified by the formation of enhancer-transcription factor-mediator-RNA polymerase II hubs in animals (Boija et al, 2018; Henninger et al, 2021; Hnisz et al, 2017; Sabari et al, 2018; Shrinivas et al, 2019)—its contribution to transcriptional repression, particularly in plants, remains largely unexplored (Peng et al, 2025; Pougy et al, 2025).

Recent studies in animals have highlighted that certain repressors and chromatin modifiers may also function via LLPS, potentially influencing chromatin architecture and epigenetic states (Larson et al, 2017; Li et al, 2020b; Strom et al, 2017). However, the mechanistic links between LLPS and gene silencing are less well defined, and in some cases remain contentious (Murphy and Boettiger, 2024; Seif et al, 2020), compared with activation. In particular, whether LLPS of transcriptional repressors contributes to large-scale epigenetic regulation, such as influencing histone modification landscapes, is not well established. This knowledge gap is especially pronounced in plants, where transcriptional repression plays critical roles in orchestrating development and environmental responses (Gao et al, 2023; Gouran and Brady, 2024; Jung et al, 2020; Li et al, 2025; Liu et al, 2014; Montez et al, 2025; Plant et al, 2021; Silva et al, 2020).

TOPLESS (TPL) and TOPLESS-related (TPR) proteins constitute a conserved family of plant transcriptional corepressors related to Tup1 in yeast and Groucho/TLE proteins in animals (Oberoi et al, 2011; Plant et al, 2021; Sprague et al, 2000). In plants, TPL/TPRs are central regulators of development, stress responses and hormone signaling. They achieve gene repression primarily through direct physical interaction with EAR (ethylene response factor-associated amphiphilic repression) motifs present in a wide range of transcriptional repressors (Plant et al, 2021). In addition, TPL/TPRs are known to associate with chromatin-remodeling and histone deacetylase (HDAC) complexes, although whether these interactions are direct or mediated through adapter proteins remains unclear (Krogan et al, 2012; Long et al, 2006; Sun et al, 2023; Zhu et al, 2010). A unique feature of plant TPLs is the evolution of a novel tetramerization interface at the N-terminus, which distinguishes them from their non-plant homologs (Martin-Arevalillo et al, 2017). This tetramerization capacity, together with the presence of multiple EAR motifs in interacting partners such as the repressor D53, or the self-dimerization/oligomerization properties of others like IAA3, provides the potential for TPL/TPRs to engage in extensive higher-order multimerization (Ke et al, 2015; Ma et al, 2017; Roy et al, 2025). Such multivalent assemblies could, in principle, reinforce transcriptional repression (Bienz, 2020), yet the functional significance of this tetramerization and higher-order organization remains unknown. In addition to their structured N-terminal tetramerization domain and C-terminal WD40 repeats, TPL/TPRs also contain intrinsically disordered regions (IDRs) in the intervening sequence (Ma et al, 2017). Whether these IDRs contribute specific functions—for example, facilitating condensate formation or mediating dynamic interactions with other proteins—has not been established. These gaps highlight the need to understand how plant-specific structural innovations such as tetramerization, multimerization with repressors, and the presence of IDRs collectively shape the repressive activity of TPL/TPRs.

Here, we show that the rice corepressor TPR2 possesses an intrinsic ability to undergo LLPS, driven cooperatively by its N-terminal tetramerization domain and a specific IDR. Structure-guided mutagenesis disrupting tetramerization or deletion of the IDR markedly impaired TPR2 condensate formation in vivo and droplet assembly in vitro. These LLPS-deficient variants failed to restore wild-type histone acetylation patterns, gene repression, and developmental phenotypes in *tpr2* mutants. We further demonstrate that TPR2 is able to co-condense with transcriptional repressors such as D53 and IAA3, thereby enabling the assembly of functional repressive complexes at target genes. Our findings reveal that LLPS is integral to TPR2-mediated transcriptional repression, linking corepressor condensate formation to chromatin state control and plant developmental regulation.

## Results

### TPR2 forms liquid-like nuclear condensates in vivo and undergoes phase separation in vitro

**Figure EV1:**
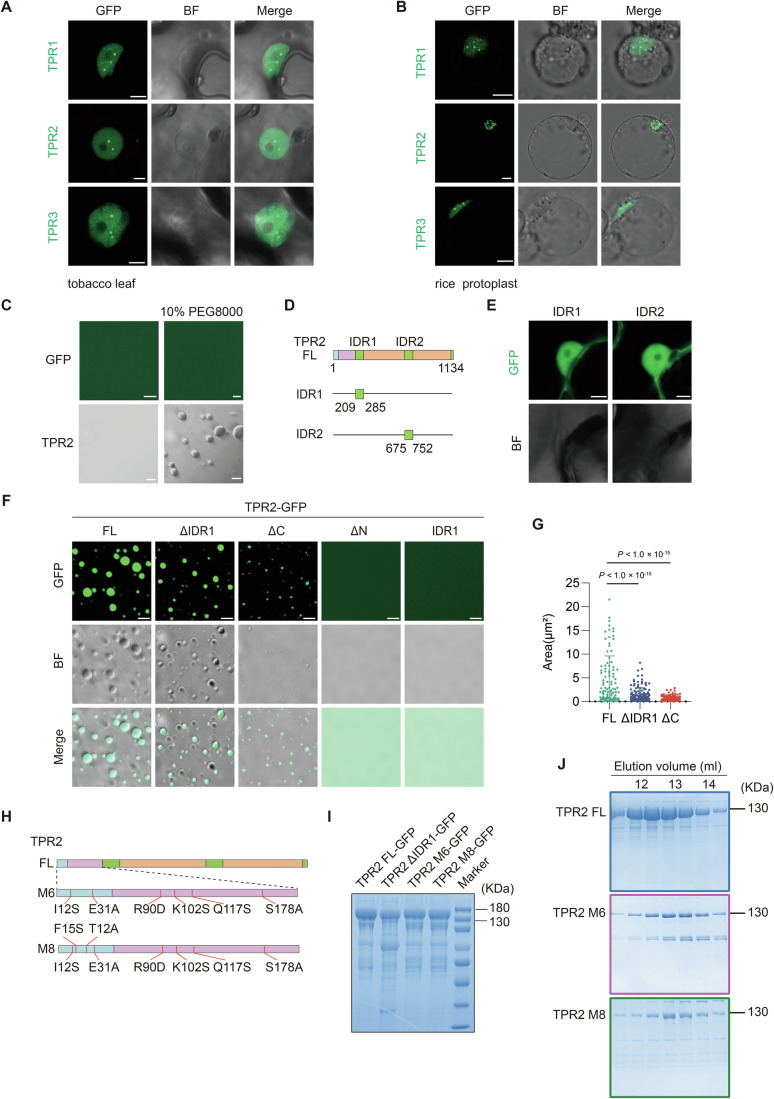
TPR2 phase separation is jointly mediated by N-terminal tetramerization and IDR1. (**A**) Confocal images showing the subcellular localization of TPR1, TPR2, and TPR3 in *N. benthamiana* leaf epidermal cells. Scale bars = 5 μm. (**B**) Subcellular localization of TPR1, TPR2, and TPR3 in rice protoplasts. Scale bars = 2 μm. (**C**) Representative images showing purified GFP and untagged TPR2 proteins incubated with 10% PEG8000. Scale bar = 5 μm. (**D**) Schematic diagram of TPR2-IDR1 and IDR2. (**E**) Confocal microscopy images of TPR2-IDR1 and IDR2. Scale bar = 2 μm. (**F**,** G**) In vitro phase separation of purified TPR2-FL, ΔIDR1, ΔC, ΔN, and IDR1 proteins in the presence of 10% PEG8000 (**F**) Proteins were labeled with GFP for visualization. Scale bar = 5 μm. (**G**) Quantification of droplet area. Data were mean ± SD (*n* = 3 fields per group); *P* values were determined by one-way ANOVA with Dunnett’s multiple-comparisons test. (**H**) Schematic representation of the M6 and M8 mutation sites in TPR2. (**I**) SDS–PAGE analysis of purified GFP-tagged TPR2-FL, TPR2-ΔIDR1, TPR2-M6, and TPR2-M8 proteins. (**J**) Fractions with the same elution volume from each injection of Fig. 2F were subjected to SDS–PAGE. Source data are available online for this figure.

**Figure 1:**
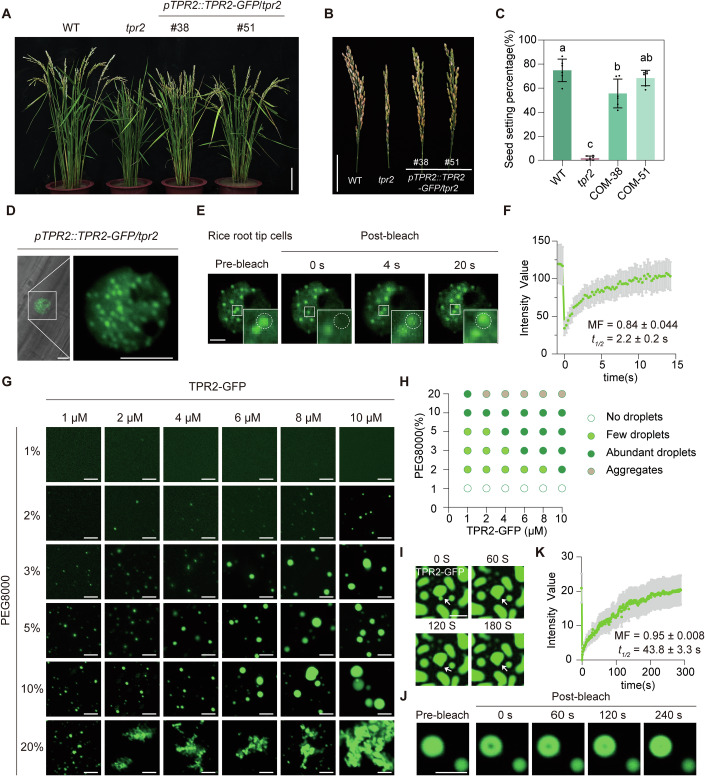
TPR2 forms liquid-like condensates in vivo and undergoes phase separation in vitro. (**A**–**C**) Plant architecture and seed setting rates of wild-type (ZH11), *tpr2* and complementary lines (*pTPR2::TPR2-GFP/tpr2*). (**A**) Plant morphology at the heading stage. Scale bar = 20 cm. (**B**) Panicle morphology. Scale bar = 5 cm. (**C**) Seed setting percentage (*n* = 6 biological replicates, each biological replicate representing the average seed setting rate of three panicles per plant). Data were mean ± SD; different lowercase letters above bars indicate a significant difference (*P* < 0.05, one-way ANOVA with Tukey’s multiple-comparisons test). The exact *P* values are listed in Dataset EV4. (**D**) Confocal images of lateral root tip cells of *pTPR2::TPR2-GFP/tpr2* seedlings at 10 d after germination. Scale bar = 10 μm. Representative images from three independent experiments. (**E**) FRAP analysis of TPR2-GFP in rice lateral root tip cells of *pTPR2::TPR2-GFP/tpr2* plant. Scale bar = 5 μm. (**F**) FRAP recovery curves of *pTPR2::TPR2-GFP/tpr2* plant. Mobile Fraction (MF) and half-time of recovery (*t*_1/2_) values are indicated on the graph. Data were mean ± SD (*n* = 3 independent experiments). (**G**) Confocal microscopy images showing TPR2 droplet formation at indicated concentrations of TPR2 and PEG8000. Scale bars = 5 μm. (**H**) In vitro TPR2-GFP phase diagram under different PEG8000 concentrations at room temperature. Scale bar = 5 μm. (**I**) Time-lapse images showing fusion of two TPR2-GFP droplets. White arrows indicate droplets before and after fusion. Scale bar = 5 μm. (**J**) FRAP analysis of TPR2-GFP droplets in vitro. Scale bar = 5 μm. (**K**) FRAP recovery curve of TPR2-GFP droplets. MF and *t*_1/2_ values are indicated on the graph. Data were mean ± SD (*n* = 3 droplets from three independent experiments). Source data are available online for this figure.

Previously, we found that the TOPLESS-related corepressor TPR2, a substrate of plant-specific serine proteinase NAL1 in rice (Li et al, 2023), formed foci in the nucleus in the *N. benthamiana* leaves and rice protoplasts. Similar condensate formation was also observed for its homologs, TPR1 and TPR3 (Fig. EV1A,B). To access the physiological relevance of these condensates, we generated transgenic lines expressing TPR2 under its own promoter in the *tpr2* mutant (*pTPR2::TPR2-GFP/tpr2*). These complementation lines restored *TPR2* transcript levels comparable to wildtype and substantially rescued the mutant phenotype (Fig. 1A–C; Appendix Fig. S1A–D). Notably, TPR2-GFP exhibited formation of nuclear puncta in rice root tip cells (Fig. 1D). Fluorescence recovery after photobleaching (FRAP) assays further demonstrated the dynamic nature of these structures, as TPR2-GFP rapidly redistributed from unbleached to bleached regions (Fig. 1E,F). Together, these observations indicate that TPR2 forms dynamic, liquid-like condensates in the plant nucleus.

To determine whether TPR2 can drive phase separation in vitro, the TPR2-GFP recombinant protein was purified in human embryonic kidney cells. PEG8000, a commonly used macromolecular crowding agent, was included to probe the intrinsic condensation propensity of TPR2 under simulated crowded cellular conditions. While PEG8000 does not fully recapitulate the physicochemical complexity of the nucleus, it will capture excluded volume effects and is routinely used in phase separation experiments (Wegmann et al, 2018). Under these conditions, purified TPR2-GFP formed condensates at protein concentrations as low as 2 μM in the presence of only 2% PEG8000 (Fig. 1G,H). Based on the TPR2–PEG phase diagram, we selected 10% PEG8000 for subsequent experiments, as this condition yielded robust and reproducible droplet formation across replicates. To exclude a potential effect of the GFP tag on LLPS behavior, we also examined untagged TPR2. Under the same buffer conditions, GFP alone remained diffuse, whereas untagged full-length TPR2 formed condensates comparable to those of TPR2-GFP (Fig. EV1C), indicating that droplet formation is an intrinsic property of TPR2. Furthermore, smaller droplets tended to coalesce into larger droplets over time, which is consistent with liquid-like properties (Fig. 1I). Similar to our observations in vivo, FRAP assays showed that the TPR2-GFP signal intensity substantially recovered after photobleaching, suggesting that TPR2-GFP have a dynamic exchange of fluorescent molecules within droplets (Fig. 1J,K). These data collectively demonstrate that TPR2 exhibits LLPS characteristics both in vivo and in vitro.

### The IDR1 and tetramerization domain contributes to TPR2 phase separation

**Figure 2:**
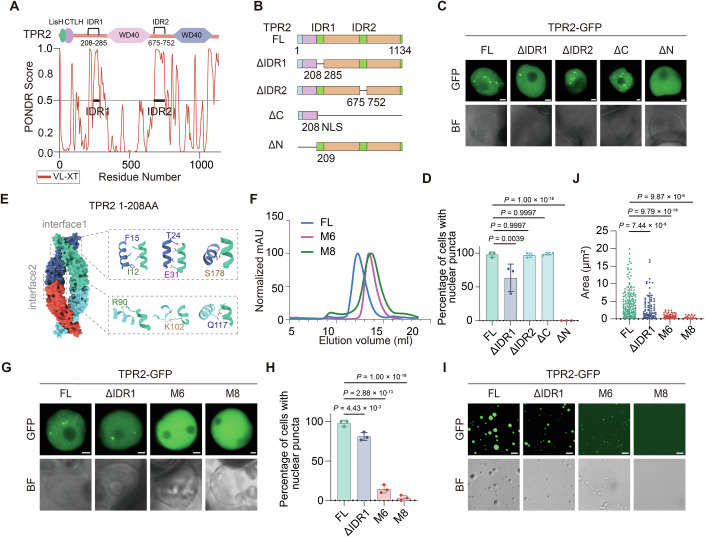
The IDR1 and tetramerization contributes to TPR2 phase separation. (**A**) Domain organization of TPR2 and prediction of IDRs using PONDR (www.pondr.com). (**B**–**D**) Subcellular localization of full-length (FL 1–1134 aa) and truncated TPR2 proteins (ΔIDR1, deletion of aa 209–285; ΔIDR2, deletion of aa 675–752; ΔC, deletion of aa 209–1134 with an added nuclear localization signal; ΔN, deletion of aa 1–208) fused to GFP at the C-terminus in *N. benthamiana* epidermal cells. (**B**) Schematic diagram of truncated proteins. (**C**) Confocal microscopy images. Scale bar = 2 μm. (**D**) Quantification of cells with nuclear puncta. Data were mean ± SD (*n* = 3 biologically independent samples; 30 transfected cells per group); *P* values were determined by one-way ANOVA with Tukey’s multiple-comparisons test. (**E**) Structural model of the N-terminal region (aa 1–208) of TPR2 showing tetramerization interfaces (right, zoomed-in view). (**F**) Elution profiles of purified TPR2-FL, TPR2-M6, and TPR2-M8 proteins subjected to size-exclusion chromatography. (**G**) Confocal images of *N. benthamiana* cells expressing TPR2-GFP or non-tetramerizing variants M6 (I12S, E31A, R90D, K102S, Q117S, and S178A) and M8 (I12S, F15S, T24A, E31A, R90D, K102S, Q117S, and S178A). Scale bar = 2 μm. (**H**) Quantification of cells with nuclear puncta. Data were mean ± SD (*n* = 3 biologically independent samples; 30 transfected cells per group); *P* values were determined by one-way ANOVA with Dunnett’s multiple-comparisons test. (**I**) In vitro droplet formation of TPR2-GFP (FL, ΔIDR1, M6, and M8) in the presence of 10% PEG8000. Scale bar = 5 μm. (**J**) Quantification of droplets. Data were mean ± SD (*n* = 3 fields per group); *P* values were determined by one-way ANOVA with Dunnett’s multiple-comparisons test. Source data are available online for this figure.

TPR2 proteins have an N-terminal domain containing a lissencephaly homologous (LisH) dimerization motif and a C-terminal to LisH (CTLH) motif. Following the conserved N-terminal domain are a proline and glutamine-rich linker and two C-terminal WD40 domains (Ke et al, 2015). Using PONDR (predictor of natural disordered regions) (Romero et al, 2001), we identified two IDRs (IDR1 and IDR2), one located between the N-terminal tetramerization domain and the first WD40 domain, and the other between the two WD40 domains (Fig. 2A; Appendix Fig. S2A–C). A number of studies have reported that protein phase separation is associated with intrinsically disordered regions (Brangwynne et al, 2015; Li et al, 2012; Nott et al, 2015). Therefore, we expressed TPR2-GFP constructs with deletion of IDR1 or IDR2 in tobacco (*N. benthamiana*) leaves, examined their subcellular localization and quantified the localization patterns. Whereas the removal of the IDR2 had little effect on TPR2 LLPS, deletion of the IDR1 domain considerably reduced, but did not eliminate, droplet formation (Fig. 2B–D). We also expressed IDR1 or IDR2 alone in tobacco leaves and found that neither formed detectable condensates in the nucleus (Fig. EV1D,E). Consistently, purified TPR2 lacking IDR1 still formed droplets in vitro, although their number and size were reduced compared with the full-length protein, whereas IDR1 alone did not undergo phase separation (Fig. EV1F,G). This suggests that, in addition to IDR1, other molecular features may contribute to TPR2 phase separation.

To identify additional regions that influence TPR2 phase separation, we individually expressed N-terminal and C-terminal deletion constructs of TPR2-GFP in tobacco leaves, examined subcellular localization and analyzed the distribution patterns of the different constructs. Compared with full-length (FL) TPR2, deletion of the N-terminal domain (ΔN, aa 209–1134) resulted in dramatically reduced phase separation ability in leaf cells and no fluorescent puncta were observed in any transfected cells. In contrast, the C-terminal truncation (ΔC, aa 1–208) showed no detectable difference in condensate formation or localization (Fig. 2B–D). Consistently, in vitro purified ΔN protein failed to undergo phase separation, whereas ΔC formed condensates with both the number and size of droplets significantly lower than those formed by FL (Fig. EV1F,G). These observations suggested that the N-terminal domain contributes to TPR2 phase separation.

The assembly of multivalent signaling proteins can promote phase separation, thus providing a molecular mechanism to explain biomolecular condensate formation and a biochemical route to understand the process of condensation (Banani et al, 2017; Li et al, 2012). Protein oligomerization often serves as an effective mechanism to enhance multivalency, thereby significantly promoting the formation of biomolecular condensates (Mitrea et al, 2016; Mitrea et al, 2018). Given that the N-terminal domain of TPR2 can form tetramers (Ke et al, 2015), we hypothesized that this oligomerization property might contribute to TPR2 phase separation by achieving the necessary threshold of multivalent interactors present in the IDRs for efficient condensation to occur. To verify this hypothesis, we examined published structural data and mapped eight amino acid residues positioned at the two interaction surfaces potentially involved in protein tetramerization (Ke et al, 2015) (Fig. 2E). To assess their functional relevance, we expressed and purified TPR2 FL and variant proteins carrying M6 mutation (contains six point mutations) or the M8 mutation (contains eight point mutations) (Fig. EV1H), and performed size-exclusion chromatography on FL, M6, and M8 proteins. Both M6 and M8 eluted at positions corresponding to approximately dimeric species, whereas FL TPR2 eluted at the expected position for a tetramer (Figs. 2F and EV1J), indicating that the M6 and M8 mutations successfully compromised the tetramerization capability of TPR2. We then expressed M6 and M8 in tobacco leaf epidermal cells; both mutants failed to form nuclear condensates in vivo, indicating that disruption of tetramerization compromises phase separation (Fig. 2G,H).

To further assess the contribution of IDR1 and tetramerization to condensate formation, we expressed and purified recombinant TPR2-GFP fusion proteins carrying the ΔIDR1, M6, or M8 mutations for in vitro assays (Fig. EV1I). Droplet-formation assays revealed that ΔIDR1 substantially reduced the ability of TPR2 to form phase-separated droplets. Likewise, both the M6 and M8 mutations impaired droplet formation in a stepwise manner, with M8 exhibiting the strongest defect (Fig. 2I, J). Taken together, these results demonstrate that both the intrinsically disordered region IDR1 and tetramerization are critical for TPR2 phase separation, and suggest that they act cooperatively to promote condensate assembly, with specific oligomerization putatively acting as a nucleation event that favors weak multivalent interactions via the IDRs.

### TPR2 regulates rice growth and development in a phase separation-dependent manner

**Figure EV2:**
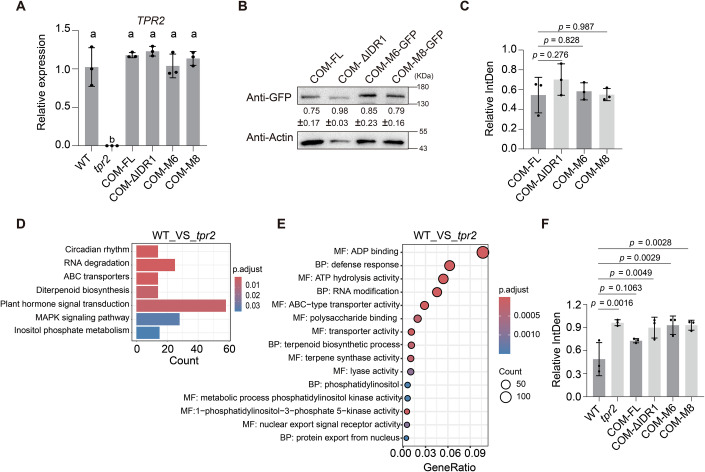
Phase separation of TPR2 negatively regulates H3K9ac levels at its transcriptionally repressed genes. (**A**) Expression levels of *TPR2* in WT, *tpr2*, COM-FL, COM-ΔIDR1, COM-M6 and COM-M8 plants. Data were means ± SD (*n* = 3 biological replicates). Different lowercase letters above bars indicate a significant difference at the *P* < *0.05* level by one-way ANOVA with Tukey’s multiple-comparisons test. The exact *P* values are listed in Dataset EV4. (**B**) Total proteins extracted from COM-FL, COM-ΔIDR1, COM-M6, and COM-M8 plants were analyzed by Western blot using anti-GFP antibody. Actin served as a loading control. Experiments were independently repeated at least three times with similar results. (**C**) Relative GFP-Tag protein levels in (**B**) were quantified by densitometric analysis and normalized to the loading control. Data were mean ± SD (*n* = 3 biological replicates). *P* values were determined by one-way ANOVA with Dunnett’s multiple-comparisons test. (**D**) Kyoto Encyclopedia of Genes and Genomes (KEGG) pathway enrichment analysis of downregulated genes in WT compared to *tpr2*. Enrichment analysis was performed using the clusterProfiler R package with Fisher’s exact test and Benjamini–Hochberg false discovery rate (FDR) correction for multiple testing. Pathways with adjusted *P* < 0.05 were considered significantly enriched. (**E**) Gene Ontology (GO) enrichment analysis of downregulated genes in WT compared to *tpr2*. Enrichment analysis was performed using the clusterProfiler R package with Fisher’s exact test and Benjamini–Hochberg false discovery rate (FDR) correction for multiple testing. GO terms with adjusted *P* < 0.05 were considered significantly enriched. (**F**) Relative H3K9ac levels in Fig. 4E were quantified by densitometric analysis, and H3 served as a loading control. Data were mean ± SD (*n* = 3 biological replicates). *P* values were determined by one-way ANOVA with Dunnett’s multiple-comparisons test. Source data are available online for this figure.

**Figure 3:**
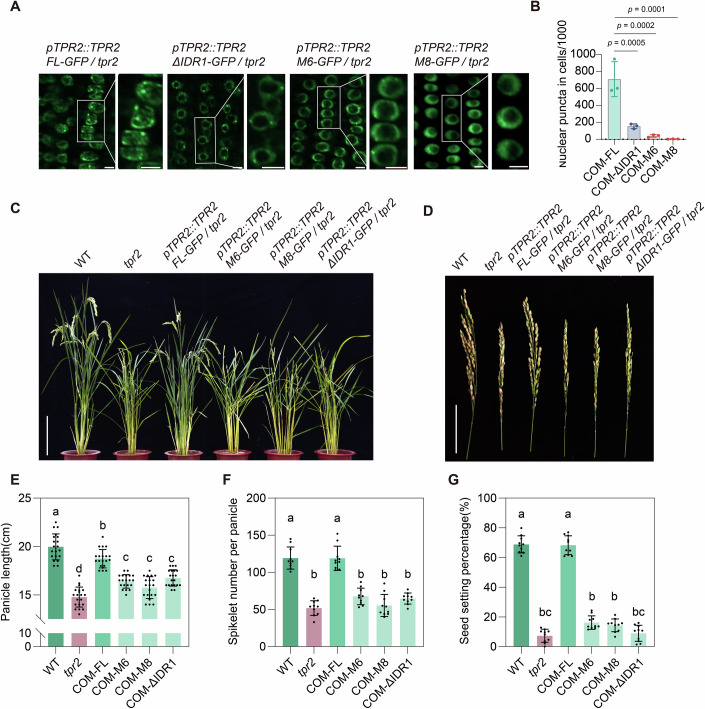
Phase separation is essential for TPR2 to regulate rice growth and development. (**A**) Confocal images of lateral root tip cells of *pTPR2::TPR2FL-GFP/tpr2* (COM-FL), *pTPR2::TPR2ΔIDR1-GFP/tpr2* (COM-ΔIDR)*, pTPR2::TPR2M6-GFP/tpr2* (COM-M6) and *pTPR2::TPR2M8-GFP/tpr2* (COM-M8). Scale bar = 5 μm. Representative images from three independent experiments. (**B**) Quantification of nuclear puncta per 1000 cells. (*n* = 3 fields per group); *P* values were determined by one-way ANOVA with Dunnett’s multiple-comparisons test. (**C**–**G**) Plant architecture and panicle-related traits of wild-type (ZH11), *tpr2* and complementary lines (COM-FL, COM-ΔIDR, COM-M6, and COM-M8). (**C**) Plant morphology at the heading stage. Scale bar = 20 cm. (**D**) Panicle morphology. Scale bar = 5 cm. (**E**) Panicle length (*n* = 20) (**F**) Spikelet number per panicle (*n* = 10) (**G**) Seed setting percentage (*n* = 10). Data in (**E**–**G**) are mean ± SD; different lowercase letters indicate significant differences (*P* < 0.05, one-way ANOVA with Tukey’s multiple-comparisons test). The exact *P* values are listed in Dataset EV4. Source data are available online for this figure.

To investigate whether the phase separation behavior of TPR2 plays a functional role in rice, we generated complementation lines expressing full-length TPR2 and its variants under the native *TPR2* promoter, including *pTPR2::TPR2-FL-GFP/tpr2* (COM-FL), *pTPR2::TPR2-ΔIDR1-GFP/tpr2* (COM-ΔIDR1), *pTPR2::TPR2-M6-GFP/tpr2* (COM-M6), and *pTPR2::TPR2-M8-GFP/tpr2* (COM-M8). Expression and immunoblot analyses indicated that TPR2 transcript and protein levels were similar among these lines (Fig. EV2A–C). Phase separation behavior was examined in the root tip nuclei of various rice complemented lines. Among them, the full-length TPR2-complemented line (COM-FL) exhibited the most prominent phase separation, characterized by clearly visible nuclear condensates. The ΔIDR1- and M6-complemented lines displayed highly attenuated phase separation, as shown by a strong decrease in the number of puncta per nucleus. In contrast, the M8-complemented line showed no detectable condensate formation (Fig. 3A,B). Moreover, in terms of plant growth and development, the complemented lines expressing full-length TPR2 closely resembled the wild type (WT) in plant height, panicle length, spikelet number per panicle, and seed-setting rate, whereas the lines expressing ΔIDR1, M6, or M8 variants exhibited phenotypes more similar to the *tpr2* mutant (Fig. 3C–G). In summary, our data provide strong evidence that phase separation is indispensable for TPR2 function in rice. The full-length TPR2, capable of forming nuclear condensates through phase separation, effectively complements the *tpr2* mutant phenotypes. In contrast, variants deficient in phase separation fail to rescue these defects, underscoring that phase separation is essential for TPR2’s biological function.

### Phase separation of TPR2 regulates genome-wide H3 acetylation

**Figure 4:**
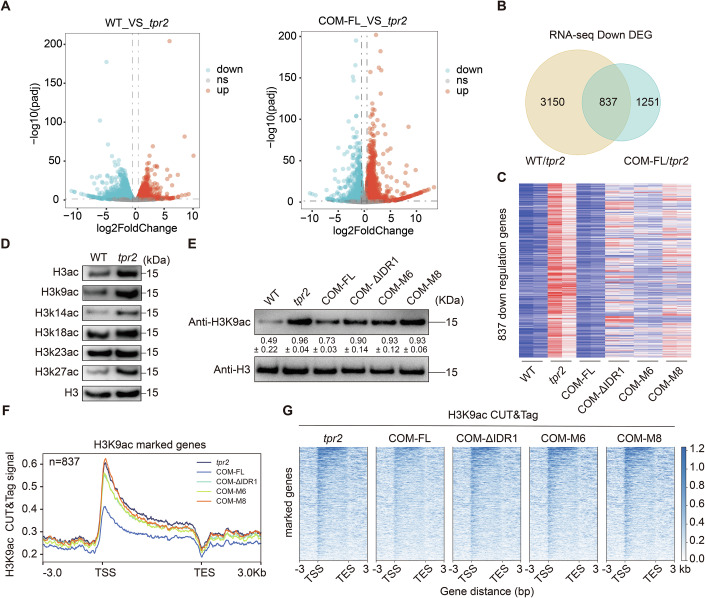
Phase separation of TPR2 negatively regulates H3K9ac levels at its transcriptionally repressed genes. (**A**) Volcano plot showing differentially expressed genes (DEGs) between WT and *tpr2* (left, *n* = 2 biological replicates per group); and between COM-FL and *tpr2* (right, *n* = 2 biological replicates per group). Differential expression analysis was performed using DESeq2 based on a negative binomial generalized linear model with Benjamini–Hochberg false discovery rate (FDR) correction for multiple testing. Genes with |log₂ (fold change)|>0.5 and adjusted *p* < 0.05 were considered significantly differentially expressed (red: upregulated; blue: downregulated; gray: not significant). (**B**) Venn diagram showing the overlap of downregulated DEGs in WT vs *tpr2* (3987 DEGs) and COM-FL vs *tpr2* (2088 DEGs). 837 DEGs were common to both comparisons. (**C**) Heatmap of 837 overlapping DEGs across WT, *tpr2*, COM-FL, COM-ΔIDR1, COM-M6, and COM-M8 plants. Gene expression levels are shown as FPKM values (red, high; blue, low). (**D**) Immunoblot analysis of histone acetylation levels in WT and *tpr2* seedlings. H3 acetylation was detected with anti-H3ac, anti-H3K9ac, anti-H3K14ac, anti-H3K18ac, anti-H3K23ac and anti-H3K27ac antibodies. Total H3 was used as a loading control. Experiments were independently repeated at least three times with similar results. (**E**) Immunoblot analysis of H3K9 acetylation levels in WT, *tpr2*, COM-ΔIDR1, COM-M6 and COM-M8 lines. Histone extracts from WT, *tpr2*, COM-ΔIDR1, COM-M6, and COM-M8 seedlings were subjected to immunoblotting using anti-acetyl-H3K9 antibody. Total H3 was used as a loading control. Experiments were independently repeated at least three times with similar results. (**F**) Density distribution of H3K9ac signals across the gene body of the 837 DEGs. Genes are grouped by genotype: *tpr2*, COM-FL, COM-ΔIDR1, COM-M6, and COM-M8. TSS, transcription start site; TES, transcription end site. (**G**) Heatmap of H3K9ac CUT&Tag signals upstream and downstream of the gene body. Regions include 3000 bp upstream of TSS and 3000 bp downstream of TES. Data were representative of two independent experiments. Source data are available online for this figure.

TPL/TPR family proteins have been widely reported to function as transcriptional corepressors by recruiting histone deacetylases, thereby reducing histone acetylation levels and suppressing gene expression (Causier et al, 2012; Jiang et al, 2020; Krogan et al, 2012; Long et al, 2006; Wang et al, 2013). To identify genes repressed by TPR2, we performed RNA-seq analysis using wild type (WT), *tpr2* mutant, and full-length complementation line (COM-FL). Violin plots depicting gene expression changes revealed a consistent down-regulation trend in both WT *vs. tpr2* and COM-FL *vs. tpr2* comparisons (Fig. 4A; Datasets EV1 and 2). Intersection analysis identified 837 genes significantly downregulated in both comparisons (Fig. 4B; Dataset EV3). The enrichment analysis recovered pathways closely related to known TPR2-associated functions. Notably, plant hormone signal transduction emerged as the most significantly enriched term in the Kyoto Encyclopedia of Genes and Genomes (KEGG) pathway analysis, and defense response was also prominently enriched in the Gene Ontology (GO) analysis, consistent with the established roles of TPR family proteins in the transcriptional regulation of growth, stress responses, and immunity (An et al, 2022; Plant et al, 2021; Wang et al, 2025). Beyond these expected categories, we also observed enrichment of secondary metabolism-related terms, suggesting that TPR2 may indirectly influence defense-associated metabolic programs. Interestingly, nuclear export-related terms were also enriched, raising the possibility that TPR2 condensates may coordinate not only transcriptional repression but also aspects of RNA or protein export, a hypothesis that warrants further investigation. (Fig. EV2D,E). Heatmap visualization showed that these genes exhibited relatively low expression in WT and COM-FL backgrounds but were substantially upregulated in *tpr2*, COM-ΔIDR1, COM-M6, and COM-M8 plants (Fig. 4C; Dataset EV3). These results indicate that TPR2 represses the expression of a set of genes, and that its phase separation ability is required for this repression.

Given the established link between histone acetylation and transcriptional activation, we next examined global histone acetylation levels in WT and *tpr2* plants. Immunoblot analyses revealed increased acetylation of total H3ac as well as H3K9ac, H3K14ac, and H3K27ac in the *tpr2* relative to WT (Fig. 4D), indicating that loss of TPR2 leads to a hyperacetylated chromatin state. We then selected H3K9ac as a representative marker for further analysis, as it exhibited the most pronounced change upon TPR2 mutation. Immunoblotting showed that H3K9ac levels were low in WT and COM-FL, but elevated in *tpr2* and in the TPR2 phase separation-defective lines COM-ΔIDR1, COM-M6, and COM-M8 (Fig. 4E). To directly assess histone acetylation at TPR2 target genes, we performed cleavage under targets and tagmentation (CUT&Tag) using anti-H3K9ac antibodies. Signal profiles over the 837 downregulated genes revealed the lowest H3K9ac enrichment in the COM-FL line, consistent with effective repression. By contrast, COM-ΔIDR1, COM-M6, and COM-M8 lines, which express phase separation-deficient TPR2 variants, showed markedly elevated H3K9ac signals, with COM-M8, carrying the most severely LLPS-impaired TPR2 variant, exhibiting levels comparable to *tpr2* (Fig. 4F; Appendix Fig. S3B). Consistently, hierarchical clustering of H3K9ac CUT&Tag signals across target genes showed that COM-ΔIDR1, COM-M6 and COM-M8 clustered closely with *tpr2* (Fig. 4G; Appendix Fig. S3B). Together, these results demonstrate that TPR2 suppresses transcription by modulating histone acetylation, and this repression depends on its capacity to undergo phase separation.

### Co-condensation of TPR2 and transcriptional repressors in vitro and in vivo

**Figure EV3:**
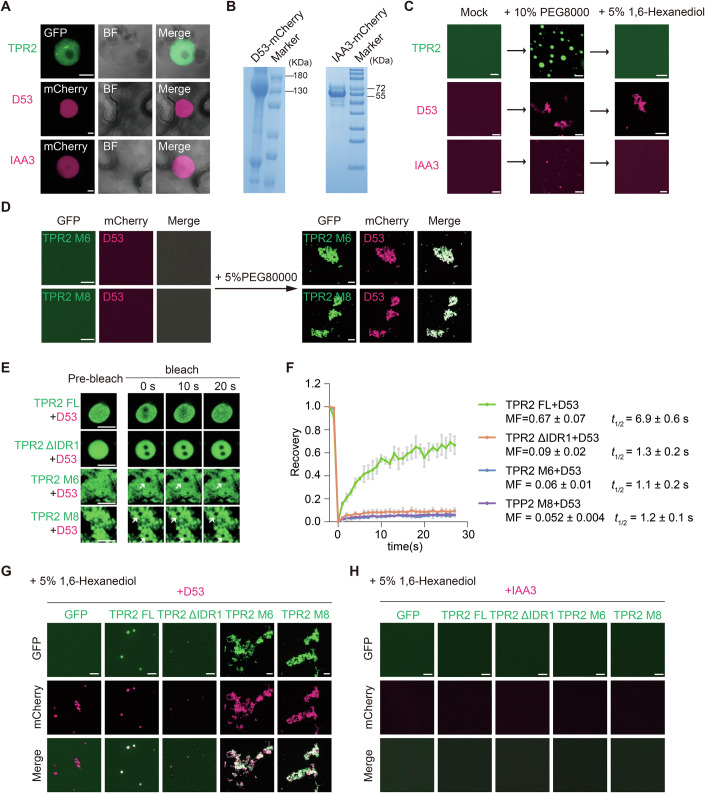
PEG-induced molecular crowding drives the formation of solid-like D53 assemblies with the phase separation-deficient TPR2 mutants. (**A**) Subcellular localization of TPR2-GFP, D53-mCherry and IAA3-mCherry in tobacco cells. Scale bar = 5 µm. (**B**) SDS–PAGE analysis of D53-mCherry and IAA3-mCherry proteins after affinity purification followed by size-exclusion chromatography. (**C**) Representative confocal images showing purified TPR2-GFP, D53-mCherry, or IAA3-mCherry proteins incubated with 10% PEG8000 followed by the addition of 5% 1,6-hexanediol. In the presence of 10% PEG8000 alone, TPR2-GFP formed large and abundant droplets, D53-mCherry formed condensed structures, and IAA3-mCherry formed small and sparse droplets. Upon addition of 5% 1,6-hexanediol, droplets formed by TPR2-GFP and IAA3-mCherry were completely dissolved, whereas D53-mCherry condensates remained visible. Scale bars = 5 μm. (**D**) Representative confocal images showing purified TPR2M6-GFP and TPR2M8-GFP proteins incubated with D53-mCherry under conditions without PEG8000 (left panels) or with 5% PEG8000 (right panels). Scale bars = 5 μm. (**E**) Representative images of FRAP assays on droplets formed by purified TPR2 FL-GFP and variants (ΔIDR1, M6, and M8) with D53-mCherry in vitro. White arrows indicate the photobleached region. Scale bars = 5 μm. (**F**) FRAP recovery curves of TPR2 FL-GFP and variants (ΔIDR1, M6, and M8) with D53-mCherry droplets. MF and *t*_1/2_ values are indicated on the graph. (**G**) Representative confocal images showing purified TPR2-FL-GFP and variants (ΔIDR1, M6, and M8) incubated with D53-mCherry in the presence of 10% PEG8000, followed by treatment with 5% 1,6-hexanediol. Under these conditions, droplets formed by TPR2-FL-GFP largely disappeared, while condensates formed by ΔIDR1, M6, and M8 variants remained visible. Scale bars = 5 μm. (**H**) Representative confocal images showing purified TPR2-FL-GFP and variants (ΔIDR1, M6, and M8) incubated with IAA3-mCherry in the presence of 10% PEG8000, followed by treatment with 5% 1,6-hexanediol. Under these conditions, phase-separated droplets formed by all TPR2 variant combinations completely disappeared. Scale bars = 5 μm. Source data are available online for this figure.

**Figure 5:**
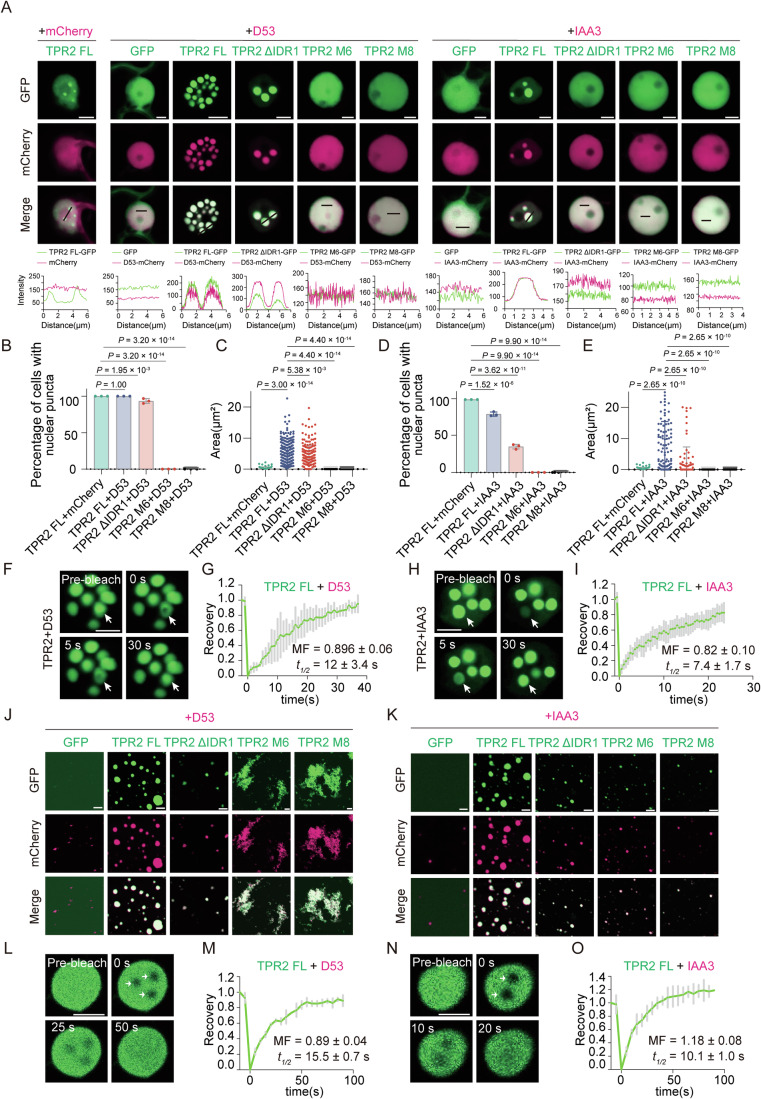
TPR2 condensates with repressors in vivo and in vitro. (**A**) Colocalization of TPR2-GFP variants with mCherry-tagged repressors. Representative confocal images (top panels) and quantification (bottom panels) showing TPR2-GFP variants (FL, ΔIDR1, M6, and M8; green) co-expressed with mCherry alone (negative control), D53-mCherry, or IAA3-mCherry (magenta). Scale bars = 5 μm. (**B**, **C**) Quantitative analysis of nuclear puncta formation with D53. (**B**) Percentage of cells showing nuclear puncta upon co-expression of TPR2 variants with D53. (**C**) Size of individual nuclear puncta in cells co-expressing TPR2 variants with D53. (**D**, **E**) Quantitative analysis of nuclear puncta formation with IAA3. (**D**) Percentage of cells showing nuclear puncta upon co-expression of TPR2 variants with IAA3. (**E**) Size of individual nuclear puncta in cells co-expressing TPR2 variants with IAA3. (**B**–**E**) Data were mean ± SD (*n* = 3 biologically independent samples; 20 transfected cells per group); *P* values were determined by one-way ANOVA with Tukey’s multiple-comparisons test. (**F**–**I**) FRAP analysis of nuclear condensates in *N. benthamiana* epidermal cells. (**F**) Representative images of FRAP assays on nuclear condensates formed by co-expression of TPR2 FL-GFP with D53-mCherry, showing a condensate before and at the indicated time points after photobleaching. White arrows indicate the photobleached region. Scale bars = 5 μm. (**G**) FRAP recovery curves of nuclear condensates formed by TPR2-GFP with D53-mCherry (*n* = 4). (**H**) Representative images of FRAP assays on nuclear condensates formed by co-expression of TPR2-FL-GFP with IAA3-mCherry. White arrows indicate the photobleached region. Scale bars = 5 μm. (**I**) FRAP recovery curves of nuclear condensates formed by TPR2-GFP with IAA3-mCherry (*n* = 10). Data in (**G**, **I**) are mean ± SD, MF and *t*_1/2_ values are indicated on the graph. (**J**,** K**) Representative images showing droplet formation of GFP or TPR2-GFP variants (FL, ΔIDR1, M6, and M8) with D53-mCherry (**J**) or IAA3-mCherry (**K**) in the presence of 10% PEG8000. Scale bars = 5 μm. (**L**–**O**) FRAP analysis of in vitro droplets. (**L**) Representative images of FRAP assays on droplets formed by TPR2 FL-GFP with D53-mCherry. White arrows indicate the photobleached region. (**M**) FRAP recovery curves of TPR2 FL-GFP with D53-mCherry droplets. (**N**) Representative images of FRAP assays on droplets formed by TPR2 FL-GFP with IAA3-mCherry. White arrows indicate the photobleached region. (**O**) FRAP recovery curves of TPR2 FL-GFP with IAA3-mCherry droplets. Scale bars in (**L**) and (**N**) = 5 μm. MF and *t*_1/2_ values are indicated on the graph. FRAP data were mean ± SD from three independent experiments. Source data are available online for this figure.

**Figure EV4:**
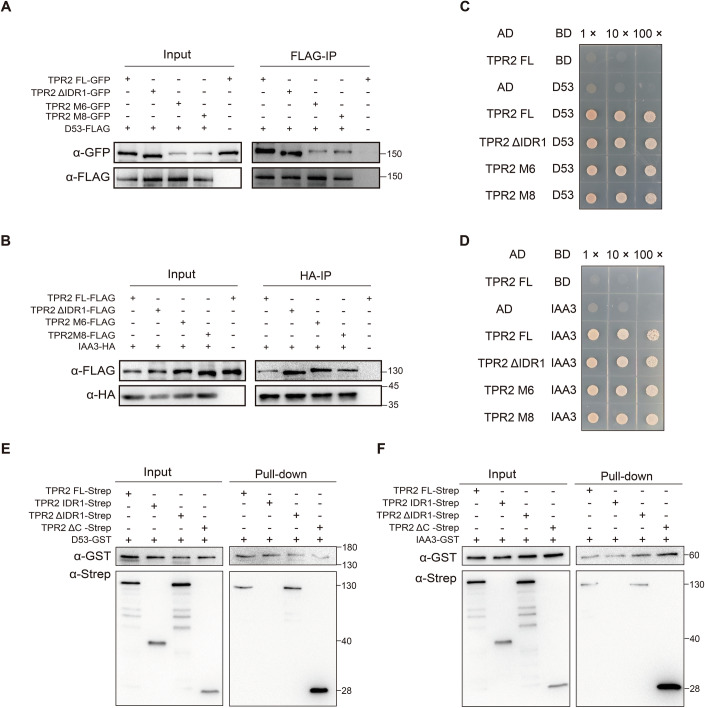
Both the full-length TPR2 and the phase separation-deficient mutants interact with repressors D53 and IAA3. (**A**,** B**) Co-IP in rice protoplasts and (**C**,** D**) Yeast two-hybrid assays showing interactions of TPR2 and the phase separation-deficient mutants with D53 and IAA3. (**E**,** F**) GST Pull-down assays indicate that the N-terminal domain of TPR2 is necessary and sufficient for direct interaction with both D53 and IAA3. Source data are available online for this figure.

TPL/TPRs are known to function as corepressors in multiple hormone signaling pathways by interacting with transcriptional repressors (An et al, 2022; Li et al, 2020a; Oh et al, 2014; Pauwels et al, 2010; Szemenyei et al, 2008; Wang et al, 2015). To further explore the molecular mechanism underlying TPR2-mediated transcriptional repression, we investigated whether TPR2 undergoes LLPS together with its interacting partners. D53 and IAA3, two representative repressors from the strigolactone and auxin signaling pathways, respectively, have been previously shown to physically interact with TPR2 (Jiang et al, 2013; Li et al, 2023). We therefore selected D53 and IAA3 to examine whether TPR2 achieves transcriptional repression through LLPS-based co-condensation with these repressors. In the absence of TPR2, D53 and IAA3 exhibited a diffused nuclear localization pattern in tobacco epidermal cells (Fig. EV3A). Upon co-expression with full-length TPR2 (TPR2-FL), both repressors formed nuclear condensates, and the number and size of TPR2/repressor puncta increased markedly, indicating enhanced phase separation, whereas, co-expression of D53 or IAA3 with TPR2-ΔIDR1, TPR2-M6, or TPR2-M8 resulted in few or no observable condensates, and D53 or IAA3 co-expressed with GFP alone did not undergo phase separation (Fig. 5A–E), suggesting that IDR1 and tetramerization are both required for co-condensation. Colocalization analysis revealed that both wild-type and mutant forms of TPR2 were localized to the nucleus and showed spatial overlap with D53 and IAA3 signals (Fig. 5A). Importantly, co-immunoprecipitation (Co-IP) and yeast two-hybrid (Y2H) assays confirmed that the TPR2 variants retained physical interactions with D53 and IAA3 (Fig. EV4A–D). This indicates that TPR2 phase separation-deficient mutants are still able to form complexes with the D53 and IAA3 repressors, but that these complexes no longer undergo LLPS. In addition, FRAP analysis showed that the fluorescence intensity of photobleached TPR2-D53/IAA3 condensates recovered rapidly, indicating dynamic molecular exchange consistent with LLPS properties (Fig. 5F–I).

To further assess the ability of TPR2 to co-phase separate with its transcriptional partners in vitro, we purified recombinant GFP-tagged TPR2-FL and its variant forms (ΔIDR1, M6, M8), along with mCherry-tagged D53 and IAA3 (Figs. EV1I and EV3B). In the presence of 10% PEG8000, D53-mCherry proteins readily formed solid-like aggregates, while IAA3-mCherry displayed only limited phase separation. Consistent with these distinct material properties, treatment with 5% 1,6-hexanediol (1,6-HD) had little effect on D53 assemblies but completely dissolved IAA3 condensates (Fig. EV3C), indicating that these two repressors differ in their intrinsic condensation behaviors. When co-incubated with TPR2-FL-GFP, both D53-mCherry and IAA3-mCherry were efficiently recruited into condensates (Fig. 5J,K). FRAP analysis showed that the mobile fractions exceeded 0.8 in both TPR2-D53 and TPR2-IAA3 co-condensates (Fig. 5L–O), indicating high molecular mobility and confirming the liquid-like properties of these assemblies. In contrast, when D53 was co-incubated with phase separation-defective TPR2 variants (ΔIDR1, M6, and M8), condensate formation was markedly impaired compared with TPR2-FL, with ΔIDR1 producing only a few condensates and M6/M8 predominantly forming large, irregular aggregates (Fig. 5J). FRAP analysis revealed that the mobile fractions of these assemblies were all below 0.1 (Fig. EV3E,F), indicating severely impaired molecular mobility and suggesting a transition toward more solid-like aggregates. Consistently, TPR2 FL-D53 condensates were largely dissolved by 1,6-HD, whereas TPR2 ΔIDR1 -D53, TPR2 M6 -D53, and TPR2 M8 -D53 assemblies were unaffected, closely resembling D53 alone (Fig. EV3G). These data suggest that, under the in vitro conditions used here, phase separation-defective TPR2 variants fail to support the dynamic co-condensate formation with D53 and are instead incorporated into solid-like assemblies. A different pattern was observed with IAA3. Co-phase separation of IAA3-mCherry showed a gradual decrease for the TPR2 mutants (ΔIDR1, M6, and M8) compared with TPR2-FL-GFP (Fig. 5K), indicating a progressive loss of droplet formation ability. However, unlike the D53-containing assemblies, all TPR2-IAA3 combinations, including those containing TPR2 variants, were fully dissolved by 1,6-HD (Fig. EV3H), indicating that these assemblies retained liquid-like properties despite reduced condensation efficiency. In summary, both in vivo and in vitro experiments demonstrate that TPR2 undergoes LLPS and acts as a key scaffold that recruits transcriptional repressors D53 and IAA3 into dynamic condensates.

### Analysis of potential target genes associated with the D53/IAA3-TPR2 co-condensate module

**Figure 6:**
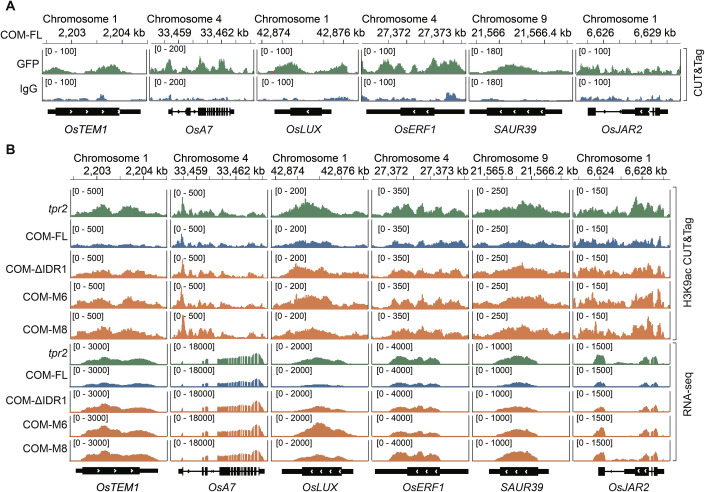
Phase separation of TPR2 promotes transcription suppression with repressors. (**A**) Integrative genome browser tracks showing GFP CUT&Tag signals at *OsTME1*, *OsA7*, *OsLUX*, *OsERF1*, *SAUR39*, and *OsJAR2* in COM-FL plants. (**B**) Integrative genome browser visualization of H3K9ac CUT&Tag (top) and RNA-seq (bottom) signals at  the same loci in *tpr2* and *pTPR2::TPR2FL-GFP/tpr2* (COM-FL), *pTPR2::TPR2ΔIDR1-GFP/tpr2* (COM-ΔIDR)*, pTPR2::TPR2M6-GFP/tpr2* (COM-M6), and *pTPR2::TPR2M8-GFP/tpr2* (COM-M8) plants. (**A**, **B**) Data from 20-day-old rice seedlings.

We demonstrated that the transcriptional corepressor TPR2 promotes the formation of nuclear-localized puncta and recruits the repressors D53 and IAA3 to these condensates. To investigate the functional role of this process in transcriptional repression, we focused on two known modules: IPA1-D53-TPR2 and ARF25-IAA3-TPR2 (Li et al, 2023; Song et al, 2017). Based on published studies and our expression data (Fig. 4), we selected representative targets: *OsTEM1* (*TEMPRANILLO1* in *Oryza sativa*) (Osnato et al, 2020), *OsA7* (*Oryza sativa H*^*+*^*-ATPases7*) (Hu et al, 2022), and *OsLUX* (*LUX ARRHYTHMO* in *Oryza sativa*)(Cai et al, 2022) (carrying IPA1-binding GTAC motifs), and *OsERF* (*Ethylene Response Factor gene in Oryza sativa*) (Hu et al, 2008), *SAUR39* (*Small Auxin-Up RNA Gene*) (Kant et al, 2009), and *OsJAR2(Oryza sativa Jasmonate Resistant 2)* (Jain et al, 2006) (carrying ARF25-binding AuxRE motifs). To determine whether TPR2 is associated with these loci in vivo, we performed GFP CUT&Tag in *pTPR2::TPR2-GFP/tpr2* plants to selectively enrich genomic loci bound by TPR2-GFP. In this approach, an anti-GFP antibody is used to selectively target the GFP-tagged TPR2 protein and thereby enrich nearby chromatin fragments, allowing high-resolution mapping of genomic regions associated with TPR2. Compared with IgG-CUT&Tag controls, significant enrichment was observed at these loci (Fig. 6A; Appendix Fig. S4A), indicating that TPR2 is associated with these potential target genes *in planta*.

**Figure EV5:**
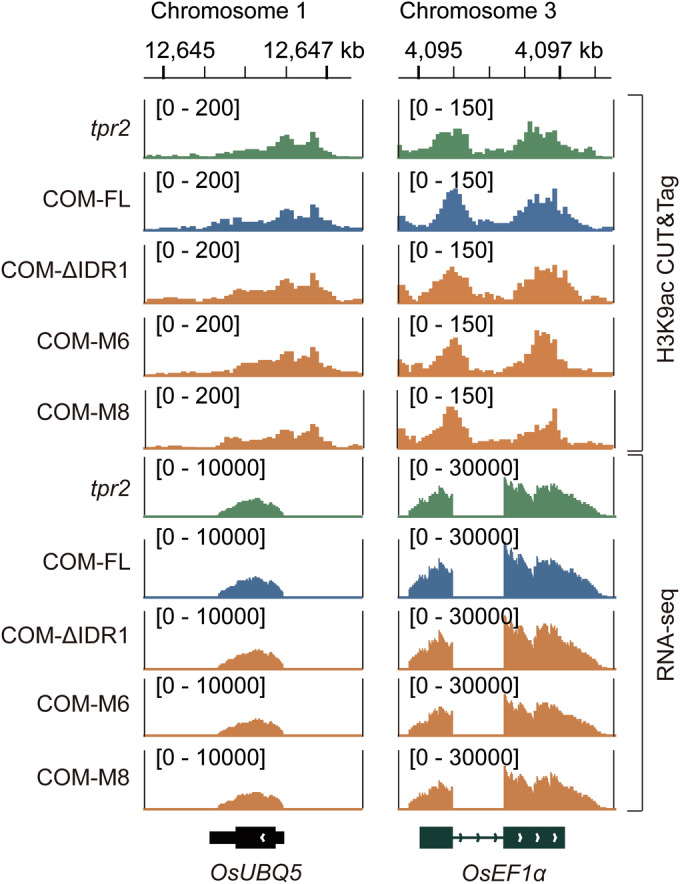
H3K9 CUT&Tag and RNA-seq signals of negative-control genes. (**A**) Integrative genome browser visualization of H3K9ac CUT&Tag (top) and RNA-seq signals (bottom) at *OsUBQ5* and *OsEF1α1* in *tpr2*, COM-FL, COM-ΔIDR, COM-M6, and COM-M8 plants.

We next examined whether TPR2 phase separation capacity correlates with chromatin and transcriptional states at these loci. CUT&Tag profiling of H3K9ac in *tpr2*, COM-FL, COM-ΔIDR1, COM-M6, and COM-M8 plants showed that H3K9ac levels at these potential target genes increase progressively as TPR2 phase separation is impaired (Fig. 6B). In parallel, expression analysis showed that these genes are more strongly repressed in COM-FL, but exhibit progressively elevated expression in COM-ΔIDR1, COM-M6, and COM-M8 (Fig. 6B). This trend was consistent with the H3K9ac changes at these loci and mirrored the graded reduction in TPR2 condensation capacity (Fig. 2F–I). By contrast, the negative-control housekeeping genes *OsUBQ5* and *OsEF1α* showed no appreciable differences either in H3K9ac levels or expression levels across the different TPR2 WT and variant complementation lines (Fig. EV5A). Together, these results, supported by the genome-wide H3K9ac profiling, TPR2 binding, transcriptomic, and H3K9ac immunoblot datasets in TPR2 WT and variants complementation lines, indicate that TPR2 condensation is associated with chromatin states and expression at a subset of potential target genes linked to the D53-TPR2 and IAA3-TPR2 pathways.

## Discussion

Despite the growing recognition of LLPS as a key principle in cellular organization, its role in transcriptional repression and chromatin silencing remains poorly understood. Most LLPS studies to date have focused on transcriptional activation, where phase-separated hubs assemble transcription factors, coactivators and RNA polymerase II to stimulate gene expression (Boija et al, 2018; Henninger et al, 2021; Hnisz et al, 2017; Sabari et al, 2018; Shrinivas et al, 2019). In contrast, how repressors and corepressors might form condensates to establish and maintain repressive chromatin states is far less explored, particularly in plants. The molecular mechanisms linking such repressive condensates to epigenetic regulation, including histone modification landscapes, are largely unknown. Here, we identify the rice corepressor TPR2 as a scaffold protein that recruits transcriptional repressors to condensates, thereby directly coupling LLPS to transcriptional repression and chromatin state control.

**Figure 7:**
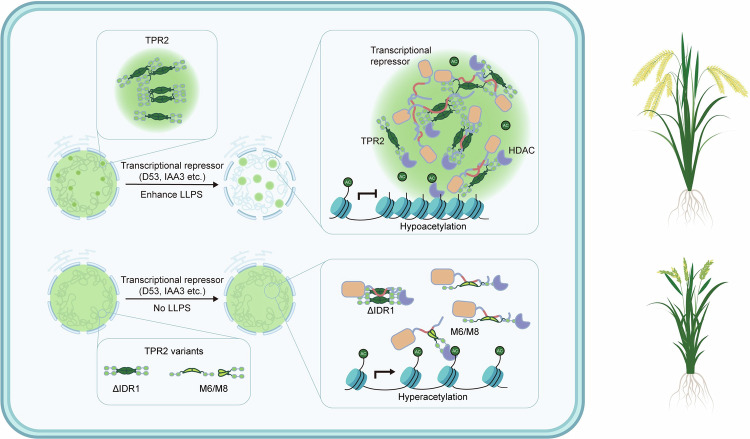
Model of TPR2-repressor-mediated epigenetic regulation and gene silencing via LLPS. In wild-type rice, TPR2 forms nuclear condensates through the cooperative action of its intrinsically disordered region (IDR1) and N-terminal tetramerization domain. These dynamic condensates recruit transcriptional repressors such as D53 and IAA3. The resulting TPR2-D53 or TPR2-IAA3 co-condensates are larger and more functionally potent than TPR2 droplets alone, facilitating robust histone deacetylation at target loci and establishing repressive chromatin states. This ensures proper regulation of genes controlling plant growth, flowering time, and panicle development. In contrast, TPR2 variants with partially or completely impaired phase separation retain physical interactions with D53 or IAA3 but fail to form functional condensates. Consequently, transcriptional repression is weakened, leading to reduced plant height, shorter panicles, fewer spikelets per panicle, and lower seed setting. This model highlights LLPS-dependent co-condensation as a key mechanism for assembling functional repressive complexes and controlling rice development.

Although IDRs are widely recognized as important contributors of LLPS, the relationship between structured oligomerization and condensate formation remains incompletely understood (Dignon et al, 2020). Our data indicate that both the N-terminal tetramerization domain and IDR1 are required for full TPR2 condensation capacity and biological activity. However, they do not establish whether oligomerization temporally precedes condensation or whether both emerge cooperatively from the same underlying multivalent interaction network. It is therefore likely that structured self-association and IDR-mediated interactions act together to promote higher-order assembly of TPR2 under cellular conditions. In this view, for proteins containing oligomerization domains that undergo LLPS, oligomerization and phase separation may not be fully separable processes, but rather mechanistically coupled features of condensate assembly. Consistent with this idea, many phase-separating proteins have been reported to contain oligomerization domains (Holehouse and Alberti, 2025; Hutin et al, 2026; Manishankar et al, 2026; Mohanty et al, 2022). In this study, we generated two TPR2 variants, M6 and M8, targeting residues within the tetramerization domain. M8 contains two additional substitutions on the basis of M6 and was initially designed to further disrupt TPR2 tetramerization. However, SEC analysis showed that both M6 and M8 elute as apparent dimers, indicating that these mutations compromise tetramerization but do not convert TPR2 into a monomeric species. Because SEC alone cannot determine which dimerization interface is retained, we illustrate two possible dimeric configurations for both mutants in our model (Fig. 7). Notably, M8 showed a broader SEC elution profile than M6 and caused stronger defects in condensate formation and chromatin regulation (Figs. 2–4), suggesting that the additional mutations in M8 may further destabilize the residual dimeric assembly or increase conformational heterogeneity. Future work to fully disrupt TPR2 tetramerization and generate bona fide monomeric variants will be important to further define how structured oligomerization controls TPR2 condensation and biological function.

Another important issue raised by our results is how to interpret the loss of function in phase separation-defective TPR2 variants in vivo. Although these mutants retain detectable interactions with D53 and IAA3, they show markedly reduced condensation capacity in vivo and fail to restore transcriptional repression, histone deacetylation, or developmental function. These observations indicate that simple binary partner binding is not sufficient for TPR2 activity and instead support the functional importance of condensation-competent higher-order repressive assemblies. At the same time, as in many studies using LLPS-defective variants, it remains difficult to determine to what extent the observed phenotypes reflect disruption of phase-separated organization itself or additional consequences of the mutations, including altered interaction dynamics, changes in assembly architecture, or impaired chromatin engagement that are not captured by Co-IP or yeast two-hybrid assays. More broadly, this highlights an important general consideration in the LLPS field: under physiological conditions, mutations that reduce condensate formation may perturb multiple interconnected properties of macromolecular assemblies, and functional defects, therefore, cannot always be assigned to loss of LLPS in isolation. Complementary approaches that perturb condensate formation without directly mutating the target protein may help address this limitation. For example, 1,6-hexanediol has been widely used to interfere with condensates mediated by weak hydrophobic interactions, although its application is more feasible at the cellular level than at the whole-plant or organismal level. Moreover, 1,6-hexanediol can non-specifically disrupt weak hydrophobic interactions and is therefore not sufficient on its own to establish LLPS. Orthogonal strategies, such as fusing solubility-enhancing tags like MBP to reduce condensation, or fusing oligomerization domains to enhance condensation, may provide additional ways to test the functional contribution of LLPS. Thus, chemical and engineering-based perturbation approaches should be interpreted together with other evidence, including concentration-dependent condensation, fusion behavior, FRAP recovery, mutational analysis, and in vivo functional assays.

Plant transcriptional repressors such as D53 contain a monocot-specific EAR motif, EAR2, which has been shown to mediate higher-order oligomerization of TPR2 by bridging tetramer-tetramer interactions and stabilizing TPR2-nucleosome binding (Ke et al, 2015; Ma et al, 2017). In the context of our study, this feature is particularly relevant because it provides an additional source of multivalency. Consistent with this idea, D53 enhances TPR2 condensation, suggesting that EAR2-containing repressors may promote the assembly of larger or more stable TPR2-containing repressive compartments. At present, however, our data do not allow us to conclude whether EAR2-dependent oligomerization is functionally important primarily because it promotes LLPS, because it strengthens chromatin engagement independently of LLPS, or because it contributes to both processes simultaneously. We therefore interpret EAR2 as a multivalent interaction module that reinforces TPR2 higher-order assembly, while the precise causal relationship between oligomerization, condensation, and chromatin repression remains to be resolved. More generally, how heterotypic condensates form, how their individual components are organized and interact within the condensed phase, and how such interactions can be quantitatively described remain important issues for future investigation.

The finding that LLPS is essential for TPR2 function opens new possibilities for “LLPS engineering” in crop improvement. By modulating IDR composition, oligomerization capacity, or interaction affinity with partner repressors, it may be possible to fine-tune the epigenetic regulation of key developmental or stress-responsive pathways. This strategy could complement existing genome-editing and transcriptional engineering approaches, offering an additional layer of control that operates through higher-order nuclear organization. However, the pleiotropic effects of LLPS modulation will require careful evaluation to avoid unintended developmental trade-offs.

In summary, we propose a working model in which TPR2 forms nuclear condensates through the cooperative action of an intrinsically disordered region and a plant-specific N-terminal tetramerization domain. These condensates act as scaffolds that dynamically incorporate repressors such as D53 and IAA3, thereby providing a platform that may locally enrich HDACs (Fig. 7). In this context, HDAC1 represents a plausible HDAC candidate, as it has been reported as a putative interactor of TPR3, a homolog of TPR2 (Tang et al, 2016). This raises the possibility that TPR2-containing condensates may participate in higher-order transcription factor-repressor-corepressor-HDAC assemblies that promote efficient deacetylation at specific loci. This condensate-based mechanism enforces transcriptional silencing by establishing hypoacetylated chromatin states, thereby shaping gene expression programs that regulate plant growth, flowering time, and panicle development. However, it is worth noting that the visible TPR2 puncta observed by confocal microscopy may represent only one form of higher-order TPR2 assembly, and smaller sub-diffraction-limit or percolated assemblies could also contribute to local HDAC recruitment, chromatin deacetylation, and transcriptional repression. More broadly, our study highlights LLPS as a versatile organizing principle in gene silencing and epigenetic regulation. In plants, the integration of LLPS into repressive regulatory circuits provides an important conceptual framework for understanding transcriptional control and suggests novel strategies for manipulating chromatin states to improve crop performance.

## Methods

**Table:** Reagents and tools table

Reagent/resource	Reference or source	Identifier or catalog number
**Experimental models**		
Rice: *Oryza sativa* ssp. japonica ZH11 wildtype	This study	
Rice: *tpr2-260*	This study	
Rice: *pTPR2::TPR2-GFP/tpr2*	This study	
Rice: *pTPR2::TPR2-ΔIDR1-GFP/tpr2*	This study	
Rice: *pTPR2::TPR2-M6-GFP/tpr2*	This study	
Rice: *pTPR2::TPR2-M8-GFP/tpr2*	This study	
**Recombinant DNA**		
**Transgenic line construction**		
*Plasmid: tpr2*	This study	
*Plasmid: pTPR2::TPR2-GFP*	This study	
*Plasmid: pTPR2::TPR2-ΔIDR1-GFP*	This study	
*Plasmid: pTPR2::TPR2-M6-GFP*	This study	
*Plasmid: pTPR2::TPR2-M8-GFP*	This study	
**Subcellular localization**		
*Plasmid: Pro35S:* GFP	This study	
*Plasmid: Pro35S:* TPR2-GFP	This study	
*Plasmid: Pro35S:* TPR1-GFP	This study	
*Plasmid: Pro35S:* TPR3-GFP	This study	
*Plasmid: Pro35S:* TPR2-YFP	This study	
*Plasmid: Pro35S:* TPR1-YFP	This study	
*Plasmid: Pro35S:* TPR3-YFP	This study	
*Plasmid: Pro35S:* TPR2 ΔC-GFP	This study	
*Plasmid: Pro35S:* TPR2 ΔN-GFP	This study	
*Plasmid: Pro35S:* TPR2 ΔIDR1-GFP	This study	
*Plasmid: Pro35S:* TPR2 ΔIDR2-GFP	This study	
*Plasmid: Pro35S:* TPR2 M6-GFP	This study	
*Plasmid: Pro35S:* TPR2 M8-GFP	This study	
*Plasmid: Pro35S:* TPR2-IDR1-GFP	This study	
*Plasmid: Pro35S:* TPR2-IDR2-GFP	This study	
*Plasmid: Pro35S:* mCherry	This study	
*Plasmid: Pro35S:* D53-mCherry	This study	
*Plasmid: Pro35S:* D53 mEAR2-mCherry	This study	
*Plasmid: Pro35S:* IAA3-mCherry	This study	
*Plasmid: Pro35S:* NAL1-mCherry	This study	
*Plasmid: Pro35S:* Blue	This study	
*Plasmid: Pro35S:* HDAC1-Blue	This study	
*Plasmid: Pro35S:* HDT701-Blue	This study	
**Protein expression and purification**		
*Plasmid:* TPR2 FL-strep	This study	
*Plasmid:* GFP-strep	This study	
*Plasmid:* TPR2 FL-GFP-strep	This study	
*Plasmid:* TPR2 ΔC-strep	This study	
*Plasmid:* TPR2 ΔC-GFP-strep	This study	
*Plasmid:* TPR2 ΔN-GFP-strep	This study	
*Plasmid:* TPR2 IDR1-GFP-strep	This study	
*Plasmid:* TPR2ΔIDR1-strep	This study	
*Plasmid:* TPR2ΔIDR1-GFP-strep	This study	
*Plasmid:* TPR2 M6-strep	This study	
*Plasmid:* TPR2 M6-GFP-strep	This study	
*Plasmid:* TPR2 M8-strep	This study	
*Plasmid:* TPR2 M8-GFP-strep	This study	
*Plasmid:* mCherry-strep	This study	
*Plasmid:* D53-mCherry-strep	This study	
*Plasmid:* IAA3-mCherry-strep	This study	
*Plasmid:* D53-GST	This study	
*Plasmid:* IAA3-GST	This study	
**Co-immunoprecipitation (Co-IP) assays**		
*Plasmid:* TPR2 FL-FLAG	This study	
*Plasmid:* TPR2 FL-GFP	This study	
*Plasmid:* TPR2ΔIDR1-FLAG	This study	
*Plasmid:* TPR2ΔIDR1-GFP	This study	
*Plasmid:* TPR2 M6-FLAG	This study	
*Plasmid:* TPR2 M6-GFP	This study	
*Plasmid:* TPR2 M8-FLAG	This study	
*Plasmid:* TPR2 M8-GFP	This study	
*Plasmid:* D53-FLAG	This study	
*Plasmid:* IAA3-FLAG	This study	
**Yeast two-hybrid(Y2H) assays**		
*Plasmid:* AD	This study	
*Plasmid:* BD	This study	
*Plasmid:* TPR2 FL -AD	This study	
*Plasmid:* TPR2ΔIDR1 -AD	This study	
*Plasmid:* TPR2 M6 -AD	This study	
*Plasmid:* TPR2 M8 -AD	This study	
*Plasmid:* D53 -BD	This study	
*Plasmid:* IAA3 -BD	This study	
**Antibodies**		
anti-FLAG	Sigma-Aldrich	Cat#F3165
anti-HA	ABclonal	Cat#AE008
anti-GFP	ABclonal	Cat#AE078
anti-Strep	ABclonal	Cat#AE066
anti-GST	ABclonal	Cat#AE001
anti-Actin	ABclonal	Cat#AC009
anti-H3ac	ABclonal	Cat#A17917
anti-H3K9ac	ABclonal	Cat#A21107
anti-H3K14ac	ABclonal	Cat#A7254
anti-H3K18ac	ABclonal	Cat#A7257
anti-H3K23ac	Abcam	Cat#Ab177275
anti-H3K27ac	Abcam	Cat#Ab4729
anti-H3K9me2	Abcam	Cat#ab1220
anti-H3	Abmart	Cat#p30266
HRP-conjugated Goat anti-Rabbit IgG (H + L)	ABclonal	Cat#AS014
HRP-conjugated Goat anti-Mouse IgG (H + L)	ABclonal	Cat#AS003
**Oligonucleotides and other sequence-based reagents**		
Oligonucleotides (5’ to3’)		
GCATGATCGAGCCAAGGCGGTGG	This study	sgRNA-TPR2
GCATGATCGAGCCAAGGCGGGTTTTAGAGCTAGAAATAGCAAGTTA	This study	TPR2-U6F
CCGCCTTGGCTCGATCATGCAACCTGAGCCTCAGCGCAGC	This study	TPR2-U6R
CGGCTTGAGCTCTGGGTTTA	This study	TPR2-260-genomic-F
TCGTTGCCAAGCAAAGATGG	This study	TPR2-260genomic-R
AGGCATGATCGAGCCAAGGC	This study	qTPR2-F
GAGCATGATATTCCGAGCTG	This study	qTPR2-R
CAGGTCGACTCTAGAGGATCCTCCCAGACCCGTTGCCTTTTTTCC	This study	Promoter TPR2-2301-F
CACCATGGATCCTCTAGAGTCGACGGCTCCGCCGATCCCAGCCTGCGC	This study	Promoter TPR2-2301-R
GCTGGGATCGGCGGAGCCGTCGACATGTCGTCGCTTAGCAGGGAGCTG	This study	TPR2-CDS-2301-F
CTCGCCCTTGCTCACCATGGATCCGACTTCTGGTTTGTTAGCTGCT	This study	TPR2-CDS-2301-R
TCTTGTGCCGCTCCTACCCATCCAAGGACTCCAACA	This study	TPR2-del209-285-F
TGTTGGAGTCCTTGGATGGGTAGGAGCGGCACAAGA	This study	TPR2-del285-209-R
TCCGGAGGTACCGGATCCTCTAGAGAATTCATGGTGAGCAAGGGCGAG	This study	PML28-mEGFP-F
GGGGTGGCTCCAGGCGCTCTCGAGCTTGTACAGCTCGTCCATGCCGAG	This study	PML28-mEGFP-R
ATTATCGATCCGGAGGTACCATGTCGTCGCTTAGCAGGGA	This study	PMLl28-TPR2-EGFP-F
TGGCTCCAGGCGCTCTCGAGGACTTCTGGTTTGTTAGCTG	This study	PMLl28-TPR2-EGFP-R
AAGAATTATCGATCCGGAGGTACCATGCCCACTCCGGTGGCCGCCGCG	This study	PML28-D53-mCherry-F
CTTGCTCACCATGAATTCTCTAGAACAATCTAGAATTATTCTTGGCGG	This study	PML28-D53-mCherry-R
AAGAATTATCGATCCGGAGGTACCATGTCGCCGCCCCTCGAGCTCGAC	This study	PML28-IAA3-mCherry-F
CTTGCTCACCATGAATTCTCTAGAGTTCCTGTTCTTGGACTTTTCCCC	This study	PML28-IAA3-mCherry-R
ATTATCGATCCGGAGGTACCATGTCGTCGCTTAGCAGGGA	This study	PML28-TPR2-F
TGGCTCCAGGCGCTCTCGAGGACTTCTGGTTTGTTAGCTG	This study	PML28-TPR2-R
GTGGCTCCAGGCGCTCTCGAGGATCATTCTTGTGCCGCTCCTCCC	This study	PML28-TPR2-N-R
AATTATCGATCCGGAGGTACCATGAATGGAGCAAGAGCTCCTCC	This study	PML28-TPR2-C-F
ATTATCGATCCGGAGGTACCATGAATGGAGCAAGAGCTCCTCC	This study	PML28-mGFP-IDR1-F
ATGAATTCTCTAGAGGATCCGCTTGCACCAGAGAAGGACAC	This study	PML28-mGFP-IDR1-R
CTGGTTCCGCGTGGATCCATGCCCACTCCGGTGGCCGCC	This study	pgex-4T-1-D53-F
ATGCGGCCGCTCGAGTCGACACAATCTAGAATTATTCTTGG	This study	pgex-4T-1-D53-R
CTGGTTCCGCGTGGATCCATGTCGCCGCCCCTCGAGCT	This study	pgex-4T-1-IAA3-F
ATGCGGCCGCTCGAGTCGACGTTCCTGTTCTTGGACTTTT	This study	pgex-4T-1-IAA3-R
TCTCGAGCTTTCGCGAGCTCATGTCGTCGCTCAGCCGGGAG	This study	TPR1-1301S-GFP-F
TTGCATGCCTGCAGGTCGACTCTTTGGATTTGGTCTGCAGC	This study	TPR1-1301S-GFP-R
TCTCGAGCTTTCGCGAGCTCATGTCGTCGCTTAGCAGGGAG	This study	TPR2-1301S-GFP-F
TTGCATGCCTGCAGGTCGACGACTTCTGGTTTGTTAGCTGCT	This study	TPR2-1301S-GFP-R
TCTCGAGCTTTCGCGAGCTCATGTCGTCGCTGAGCCGGGAG	This study	TPR3-1301S-GFP-F
TTGCATGCCTGCAGGTCGACTCTTTCTGGTTGATCAGAACTT	This study	TPR3-1301S-GFP-R
TTGCATGCCTGCAGGTCGACGTCCGAACGGAGCCGCTTGGGAGG	This study	TPR2-208N-1301S-GFP-R
TCTCGAGCTTTCGCGAGCTCAATGGAGCAAGAGCTCCTCC	This study	TPR2-209-1301S-F
GCAGATCTATCGATTCTAGAATGTCGTCGCTCAGCCGGGAG	This study	OsTPR1-FL-PM999-YFP-F
TTGCTCACCATGGCTCTAGATCTTTGGATTTGGTCTGCAGC	This study	OsTPR1-FL-PM999-YFP-R
GCAGATCTATCGATTCTAGAATGTCGTCGCTTAGCAGGGAG	This study	OsTPR2-FL-PM999-YFP-F
TTGCTCACCATGGCTCTAGAGACTTCTGGTTTGTTAGCTGCT	This study	OsTPR2-FL-PM999-YFP-R
GCAGATCTATCGATTCTAGAATGTCGTCGCTGAGCCGGGAG	This study	OsTPR3-FL-PM999-YFP-F
TTGCTCACCATGGCTCTAGATCTTTCTGGTTGATCAGAACTT	This study	OsTPR3-FL-PM999-YFP-R
TCTTGTGCCGCTCCTACCCATCCAAGGACTCCAACA	This study	R2-del209-285-1301S-GFP-F
TGTTGGAGTCCTTGGATGGGTAGGAGCGGCACAAGA	This study	R2-del285-209-1301S-GFP-R
TCTCGAGCTTTCGCGAGCTCATGGAGAGCAGAGCATATGAGGGCTC	This study	R2-del672-753-1301S-GFP-F
TTGCATGCCTGCAGGTCGACTGCCAACTTCCAGGTTTTAACCT	This study	R2-del672-753-1301S-GFP-R
AAAGATGATGATGATAAAGGTACCATGCCCACTCCGGTGGCCGCCGCG	This study	D53-1301S-mcherry-F
CAGGTCGACTCTAGAGGATCCACAATCTAGAATTATTCTTGGCGG	This study	D53-1301s-mcherry-R
AAAGATGATGATGATAAAGGTACCATGTCGCCGCCCCTCGAGCTC	This study	IAA3-1301S-mCherry-F
CAGGTCGACTCTAGAGGATCCGTTCCTGTTCTTGGACTTTTC	This study	IAA3-1301S-mCherry-R
TACGAACGATAGCCGGTACCATGTCGTCGCTTAGCAGGGAG	This study	TPR2-1301U-FLAG-F
TGATCTTTGTAATCGGATCCGACTTCTGGTTTGTTAGCTGC	This study	TPR2-1301U-FLAG-R
TTCCAGATTACGCGGGTACCATGTCGCCGCCCCTCGAGCT	This study	IAA3-PM999-3*HA-F
AACGATCGGGAATTGGATCCCTAGTTCCTGTTCTTGGACT	This study	IAA3-PM999-3*HA-R
TTTCACCATTTACGAACGATAGCCGGTACCATGCCCACTCCGGTGGCCGC	This study	D53-mCherry-1301u-F
GTCACCATCATGATCTTTGTAATCGGATCCCTTGTACAGCTCGTCCATGC	This study	D53-mCherry-1301u-R
TGGCCATGGAGGCCGAATTCATGTCGTCGCTTAGCAGGGA	This study	TPR2-BD-F
GCTGCAGGTCGACGGATCCCTCAGACTTCTGGTTTGTTAG	This study	TPR2-BD-R
CCATGGAGGCCAGTGAATTCATGCCCACTCCGGTGGCCGCCGCG	This study	D53-AD-F
AGCTCGAGCTCGATGGATCCTCAACAATCTAGAATTATTCTTGG	This study	D53-AD-R
CCATGGAGGCCAGTGAATTCATGTCGCCGCCCCTCGAGCT	This study	IAA3-AD-F
AGCTCGAGCTCGATGGATCCCTAGTTCCTGTTCTTGGACT	This study	IAA3-AD-R
**Chemicals, Enzymes and other reagents**		
Pierce™ Anti-DYKDDDDK Magnetic Agarose	Thermo Fisher Scientific	Cat#A36797
Pierce™ Anti-HA Magnetic Beads	Thermo Fisher Scientific	Cat#88837
Glutathione Sepharose 4B	GE Healthcare	Cat#17-0756-05
Hyperactive Universal CUT&Tag Assay Kit for Illumina Pro	Vazyme	Cat#TD904
Agencourt AMPure XP beads	Beckman Coulter	Cat#A63881
Strep-Tactin® resins	IBA Lifesciences	Cat#2-1505-010
EpiQuik Total Histone Extraction Kit	Epigentek	Cat#OP-0006-100
*Escherichia coli* DH5α	Tsingke	Cat#TSC-C14
*Escherichia coli* BL21(DE3)	TransGen Biotech	Cat#CD601
*Agrobacterium tumefaciens* AH105	This paper	
*Agrobacterium tumefaciens* GV3101	WEIDI	Cat#AC1003
Expi293FTM	Invitrogen	Cat#: A14527
**Software**		
PONDR	https://www.pondr.com	
IGV	https://igv.org/	Version 2.19.1
R	https://www.r-project.org/	Version 4.4.2
ZEN	https://www.zeiss.com.cn/	Version 3.4
GraphPad Prism	https://www.graphpad.com/	Version 8
ImageJ	https://imagej.net/	Version 1.53q
**Other**		
Illumina HiSeq 2000	Illumina	
Illumina HiSeq-PE150	Illumina	

### Plant materials

To construct a *TPR2*-knockout line using CRISPR-Cas9 technology, the 20 bp-specific single guide RNA target sequence of *TPR2* was designed and cloned into the TKC vector driven by the OsU6 promoter (He et al, 2018). The resulting vector was then transformed into rice calli of wild-type ZH11 (*O. sativa ssp. japonica*) through *Agrobacteria*-mediated transformation. The homozygous mutants, named *tpr2*, were identified by sequencing and polymerase chain reaction (PCR) analysis.

To construct the complement lines in the *tpr2* background, the full-length coding sequences (CDS) of *TPR2*, *TPR2ΔIDR1*, *TPR2M6*, and *TPR2M8*, each driven by the *TPR2* promoter (~2 kb) from ZH11 were cloned into the pCAMBIA2301-GFP vector. The resulting vectors were transformed into rice calli of homozygous *tpr2* through *Agrobacteria*-mediated transformation. The positive transgenic lines were identified by PCR.

### Protein expression and purification

TPR2-FL/ΔIDR1/M6/M8 was cloned into a modified pMLink vector with C-terminal Strep-Tag II and EGFP-tag. D53 and IAA3 were cloned into a modified pMLink vector with C-terminal Strep-Tag II and mCherry-tag. The construct was transformed into the human embryonic kidney 293 cell line (Expi293FTM (Invitrogen) cat#: A14527), and cultured in a 37 °C thermostatic shaker containing 5% CO_2_ at 150–175 rpm for 60 h. Then harvest and lysis cells with buffer W (100 mM Tris-HCl, pH 8.0, 150 mM NaCl, 1 mM EDTA), and lysed using a high-pressure cell disrupter (JNBIO). The cell lysates were centrifuged at 16,000×*g* for 1 h at 4 °C. The supernatant containing soluble proteins was loaded onto a column equipped with Strep-Tactin® resins (cat.No.2-1505-010 IBA Lifesciences), washed with buffer W and eluted with buffer E (100 mM Tris-HCl, pH 8.0, 150 mM NaCl, 1 mM EDTA, 5–10 mM d-Desthiobiotin). The purified protein was concentrated and subjected to gel-filtration chromatography (Superose 6; GE Healthcare, catalog no. 29-0915 96) in a buffer containing 25 mM Tris-HCl, pH 8.0, 150 mM NaCl, and 2 mM dithiothreitol. Proteins during all purification processes were subjected to SDS–polyacrylamide gel electrophoresis (SDS–PAGE) and visualized by Coomassie Blue staining.

### Size-exclusion chromatography (SEC) for TPR2 and variants

TPR2-FL/ΔIDR1/M6/M8 was cloned into a modified pMLink vector with C-terminal Strep-Tag II. The construct was transformed into the human embryonic kidney 293 cell line (Expi293FTM (Invitrogen) cat#: A14527) and purified via affinity chromatography. Purified proteins were concentrated by SEC.

SEC was performed on a Superose 6 10/300 GL column (GE Healthcare) equilibrated with SEC buffer (20 mM Tris-HCl, pH 8.0, 150 mM NaCl, and 2 mM DTT) at 4 °C. Protein samples (500–1000 μL) were loaded and eluted at 0.5 mL/min, with absorbance monitored at 280 nm.

The apparent molecular weight of M6 and M8 mutants was roughly estimated by comparing their elution positions with that of TPR2-FL, which forms a dimer under these conditions. Peak shifts were used solely to infer relative oligomeric states, without precise molecular weight determination.

### Droplet-formation assay

TPR2 FL-GFP recombinant proteins were diluted in 20 mM Tris-HCl (pH 8.0) and 150 mM NaCl to final concentrations of 1–20 μM. Phase separation was induced by mixing the protein solution with PEG8000 (1–40% w/v) at a 1:1 ratio (protein: PEG). Droplet formation was observed under a fluorescence microscope.

### Subcellular localization

Microscopy images were acquired with a Zeiss LSM980 confocal microscope. To determine the subcellular localization of TPR2 in rice plants, we observed the 7-day-old seedlings' root tips of *ProTPR2::TPR2-GFP/tpr2* transgene plants. To determine the subcellular localization of TPR2 in rice protoplasts, the construct 35S::TPR2 was transformed into rice protoplasts. Fluorescence was examined 16 h post-transformation. To determine the subcellular localization of the TPR2 in *N.benthamiana* epidermal leaf cells, the coding sequences of TPR2 and its variants were cloned into pCAMBIA1301-35S-GFP vector, the coding sequences of D53 and IAA3 were cloned into pCAMBIA1301-35S-mCherry vector, respectively. The constructs were transformed into Agrobacteria GV3101 (WEIDI, AC1003). Different combinations as indicated were transiently expressed in tobacco leaves, and fluorescence was examined 36 h post-transformation.

### Yeast two-hybrid assays

The coding sequence of the TPR2 and its variants were cloned into the pGBKT7 vector (binding domain), while the full-length coding sequence of D53 and IAA3 were cloned into the pGADT7 vector (activation domain). These constructs were co-transformed into the Y2H Gold yeast strain. Co-transformations with empty pGBKT7 and pGADT7 vectors served as negative controls. Yeast transformants were first selected on SD/-Leu/-Trp medium for 2–3 days to confirm the presence of both plasmids. To test for protein-protein interactions, transformants were transferred to SD/-Ade/-His/-Leu/-Trp medium, and growth was assessed after 3–4 days. Positive growth indicated a potential interaction between TPR2 (and its variants) with D53 or IAA3.

### Co-immunoprecipitation (Co-IP) assays

The coding sequences of TPR2-FL, TPR2-ΔIDR1, TPR2-M6, and TPR2-M8 were cloned into both pCAMBIA1301-EGFP and pCAMBIA1301-35S-FLAG vectors. D53 was cloned into pCAMBIA1301-FLAG, and IAA3 into pM999-HA. GFP-tagged TPR2 and FLAG-tagged D53, or FLAG-tagged TPR2 and HA-tagged IAA3, were co-expressed in rice leaf protoplasts. After 14–16 h of incubation at room temperature, protoplasts were harvested and lysed in Co-IP buffer (50 mM Tris-HCl, pH 7.5, 150 mM NaCl, 1 mM EDTA, 0.5% Triton X-100, 1 mM DTT, 1 mM PMSF, and 1× protease inhibitor cocktail [Roche]). Cell lysates were cleared by centrifugation, and supernatants were incubated with anti-FLAG or anti-HA magnetic beads (Thermo Fisher Scientific) for 6 h. Beads were washed with Co-IP buffer, and bound proteins were eluted by boiling in SDS–PAGE loading buffer at 95 °C for 10 min. Eluted proteins were resolved by 10% SDS–PAGE and analyzed by immunoblotting with anti-FLAG (Sigma-Aldrich, 1:5000), anti-HA (ABclonal, AE008, 1:3000), or anti-GFP (ABclonal, AE078, 1:3000) antibodies.

### In vitro pull-down assays

D53 and IAA3 were separately cloned into the GST-tagged pGEX-4T-1 vector and expressed in *E. coli*, TPR2 was cloned into the StrepII-tagged pMlink vector and expressed in the human embryonic kidney 293 cell line. In the GST pull-down assays, purified recombinant proteins were added in GST-binding buffer (20 mM Tris-HCl, pH 8.0, 200 mM NaCl, 1 mM MgCl_2_, 0.5% CA-630, 1 mM EDTA, and 1× complete protease inhibitor cocktail [Roche]) as indicated. Each protein mixture was incubated with 30 μL GST beads for 4 h at 4 °C and washed five times with binding buffer. After incubation, the beads were added to 1× loading buffer and boiled for 10 min. Samples were then detected separately by western blotting with anti-GST (ABclonal, catalog no. AE001; 1:5000 dilution) and anti-Strep (ABclonal, catalog no. AE066; 1:5000 dilution) antibody and anti-mouse IgG (ABclonal, catalog no. AS003; 1:10,000 dilution) as a secondary antibody.

### Fluorescence recovery after photobleaching (FRAP) assay

FRAP of TPR2-GFP condensates in rice root tip cells and TPR2-GFP with D53-mCherry or IAA3-mCherry complex bodies in *N. benthamiana* leaf epidermal cells was performed on a Zeiss LSM980 microscope using a ×40 objective. Nuclear puncta were excited with a 488-nm laser. A defined nuclear region of interest (ROI) was photobleached using 60% laser power with ten iterations. Two prebleach images were collected, followed by time-lapse acquisition at 1-s intervals for 30 s post-bleaching. Fluorescence intensity within the bleached ROI was quantified using ZEN software. Recovery curves were normalized to the pre-bleach intensity and corrected for overall photobleaching using a non-bleached reference region.

FRAP of TPR2-GFP droplets was performed on a Zeiss LSM980 microscope using a ×40 objective. The central region of a TPR2 droplet was bleached using a laser intensity of 100% at 488 nm with 20 iterations. Fluorescence recovery was recorded every 1 s for 600 s after bleaching. Images were acquired using ZEN software. Recovery curves were normalized to the pre-bleach intensity and corrected for overall photobleaching using a non-bleached reference region.

FRAP data were normalized to pre-bleach intensity. The mobile fraction (MF) was determined from the plateau phase of the recovery curve by averaging the final three time points. The half-time of recovery (t₁/₂) was calculated as the time required to reach 50% of maximal recovery using linear interpolation. All analyses were performed using custom scripts in GraphPad Prism.

### RNA-seq and data analysis

Two-week-old plants of WT, *tpr2*, *pTPR2::TPR2-FL-GFP/tpr2* (COM-FL), *pTPR2::TPR2-ΔIDR1-GFP/tpr2* (COM-ΔIDR1), *pTPR2::TPR2-M6-GFP/tpr2* (COM-M6), and *pTPR2::TPR2-M8-GFP/tpr2* (COM-M8) without roots were harvested for total RNA extraction. Library construction and sequencing were performed by Novogene Inc (Tianjin, China) with HiSeq-PE150 (Illumina Inc., San Diego, CA, USA) using the paired-end sequencing strategy. Raw reads were first subjected to quality control using Fastp (v.0.23.4). Then, clean reads were mapped to the rice genome (Nipponbare MSU7.0) using HISAT2 (v.2.2.1) with default parameters. The read numbers mapped to each gene were counted using HTSeq (v0.11.2). The differential expression analysis was performed using the DESeq2 R package (v1.30.1). DEGs were identified as those with at least onefold change (|log2FC|>0.5) and an adjusted *P* value less than 0.05. Gene expression was quantified by StringTie (1.3.0-foss-2016b) software with RPKM (reads per kilobase of transcript per million mapped reads) normalization.

### CUT&Tag and data analysis

CUT&Tag assay was performed as described previously with modifications (Kaya-Okur et al, 2019). Nuclei were isolated from 0.5 g of 14-day-old rice leaves. Tissue was homogenized in ice-cold PBS buffer (1× PBS buffer, 1% protease inhibitor) and centrifuged at 600×*g* for 5 min at 4 °C. The pellet was washed twice with M2 buffer (1× PBS, 1% protease inhibitor) and M3 buffer (1× PBS buffer, 5% sucrose, 0.05% Tween-20, 2 μM MgCl₂, 1% protease inhibitor), respectively. Finally, the purified nuclei were resuspended in 100 μl Wash buffer (20 mM Tris-HCl, pH 7.5, 150 mM NaCl, 1 mM MgCl₂, 10% glycerol, 1% protease inhibitor) and immobilized to concanavalin A-coated beads (Vazyme, TD902) with incubation at room temperature for 10 min. CUT&Tag assays were performed using the Vazyme TD904 CUT&Tag Kit. The bead-bound nuclei were incubated in 50 μl of antibody buffer (Antibody Buffer (-), with 5% Digitonin) with 1:50 anti-H3K9ac (Abclonal, A21107) antibody dilution at 4 °C by rotating overnight. The next day, antibody-incubated nuclei were resuspended in 50 µl of Dig-wash buffer with a 1:100 dilution of secondary antibody (Vazyme, TD902) and incubated at room temperature with gentle rotation for 1 h. To eliminate binding antibodies, nuclei were washed three times with 200 µl Dig-wash buffer. For tagmentation, 2 μl of pA/G-Tnp Pro was diluted in 98 μl Dig-300 buffer (final concentration 0.04 μM) and added to each sample (100 μl total volume), followed by rotation at room temperature for 1 h. pA-Tn5-bound nuclei were washed with 200 µl of Dig-300 buffer three times. Following antibody incubation, nuclei-pA/G-Tnp complexes were resuspended in 50 μl TTBL (prepared by mixing 40 μl Dig-300 buffer with 10 μl 5× TTBL) and incubated at 37 °C for 60 min in a PCR machine without a heated lid. After incubation, 2 μl 10% SDS was added and incubated at 55 °C for 10 min with gentle mixing. Supernatants were collected by magnetic separation and transferred to new tubes for subsequent steps. DNA Extract Beads Pro (25 μl; Vazyme TD904 kit) were washed twice with 200 μl 1× B&W buffer and resuspended in 50 μl 2× B&W buffer for subsequent DNA extraction. Fragmented DNA was purified using DNA Extract Beads Pro (Vazyme TD904 kit). Briefly, 50 μl of pre-washed DNA Extract Beads Pro was added to the supernatant from the tagmentation reaction, mixed thoroughly, and incubated at room temperature for 20 min. Beads were captured with a magnetic rack, washed twice with 200 μl 1× B&W buffer, and air-dried for 2–5 min. DNA was eluted in 15 μl ddH₂O for PCR amplification. Purified genomic DNA was amplified using the universal i5 primer and barcoded i7 primer (Vazyme, TD202) using Vazyme Master Mix (Vazyme, TD902).

Library PCR products were cleaned with Agencourt AMPure XP beads (Beckman Coulter, A63881) and sequenced on Illumina HiSeq 2000. Raw sequencing reads were processed with FastP (v0.23.2) to remove low-quality reads and trim low-quality bases and adapter sequences using default parameters. The quality-controlled reads were aligned to the rice reference genome (MSU7.0) with Bowtie2 (v2.3.5.1) using default settings.(Langmead and Salzberg, 2012). Peak calling was performed on non-redundant, uniquely mapped reads by MACS2 v.2.2.7.1 with the parameters “-f BAMPE -q 0.05 -call-summits” (Zhang et al, 2008). Peaks called in at least two replicates were used for differential modification analysis by R package DiffBind v.3.6.1 (Ross-Innes et al, 2012) with its built-in DESeq2 analyser. Peaks with |log2 (fold change)| >1 and false discovery rate (FDR) <0.05 were defined as differentially modified regions, and their associated genomic features were annotated using the R package ChIPseeker (Yu et al, 2015). Then, duplicated reads were discarded using samtools (v1.9) (Li et al, 2009). Wig files produced by deepTools (v2.5.3)(Ramírez et al, 2016) software with RPKM normalization were used for data visualization by IGV (version 2.19.1). The binding intensity Heatmaps and Profiles calculated by deepTools (v3.5.5).

## Supplementary information

Appendix

Peer Review File

Dataset EV1

Dataset EV2

Dataset EV3

Dataset EV4

Source data Fig. 1

Source data Fig. 2

Source data Fig. 3

Source data Fig. 4

Source data Fig. 5

EV Figure Source Data

Appendix Figure Source Data

Expanded View Figures

## Data Availability

The sequencing datasets generated in this study have been deposited in the NCBI Sequence Read Archive (SRA) and are available for reviewer access: The H3K9ac CUT&Tag data were available under accession number PRJNA1320512 at: https://dataview.ncbi.nlm.nih.gov/object/PRJNA1320512?reviewer=g0egibvslr1t1d7vd0n26p4igp. The GFP CUT&Tag data are deposited under accession number PRJNA1316634 at: https://dataview.ncbi.nlm.nih.gov/object/PRJNA1316634?reviewer=okuh10el1l575f33ep0em2jidg. The RNA-seq dataset is available under accession number PRJNA1319274 at: https://dataview.ncbi.nlm.nih.gov/object/PRJNA1319274?reviewer=f7sotie2i3uemlt5jt8p6t0sa4. The source data of this paper are collected in the following database record: biostudies:S-SCDT-10_1038-S44318-026-00852-7.
