# Supplementary material for: The TPR2 corepressor forms condensates with repressors to fine-tune growth and development in rice
Source: EMBO J. 2026 Jul 6;45(15):5330–56. doi: 10.1038/s44318-026-00852-7 (PMC13434662; doi:10.1038/s44318-026-00852-7)
Supplement: Supplementary file 1 [file 44318_2026_852_MOESM14_ESM.pdf]

Expanded View Figures

Figure EV1. TPR2 phase separation is jointly mediated by N-terminal tetramerization and IDR1.

(**A**) Confocal images showing the subcellular localization of TPR1, TPR2, and TPR3 in *N. benthamiana* leaf epidermal cells. Scale bars = 5 μm. (**B**) Subcellular localization of TPR1, TPR2, and TPR3 in rice protoplasts. Scale bars = 2 μm. (**C**) Representative images showing purified GFP and untagged TPR2 proteins incubated with 10% PEG8000. Scale bar = 5 μm. (**D**) Schematic diagram of TPR2-IDR1 and IDR2. (**E**) Confocal microscopy images of TPR2-IDR1 and IDR2. Scale bar = 2 μm. (**F**, **G**) In vitro phase separation of purified TPR2-FL, ΔIDR1, ΔC, ΔN, and IDR1 proteins in the presence of 10% PEG8000 (**F**) Proteins were labeled with GFP for visualization. Scale bar = 5 μm. (**G**) Quantification of droplet area. Data were mean ± SD ($n = 3$ fields per group); P values were determined by one-way ANOVA with Dunnett's multiple-comparisons test. (**H**) Schematic representation of the M6 and M8 mutation sites in TPR2. (**I**) SDS–PAGE analysis of purified GFP-tagged TPR2-FL, TPR2-ΔIDR1, TPR2-M6, and TPR2-M8 proteins. (**J**) Fractions with the same elution volume from each injection of Fig. 2F were subjected to SDS–PAGE. Source data are available online for this figure.

© The Author(s)

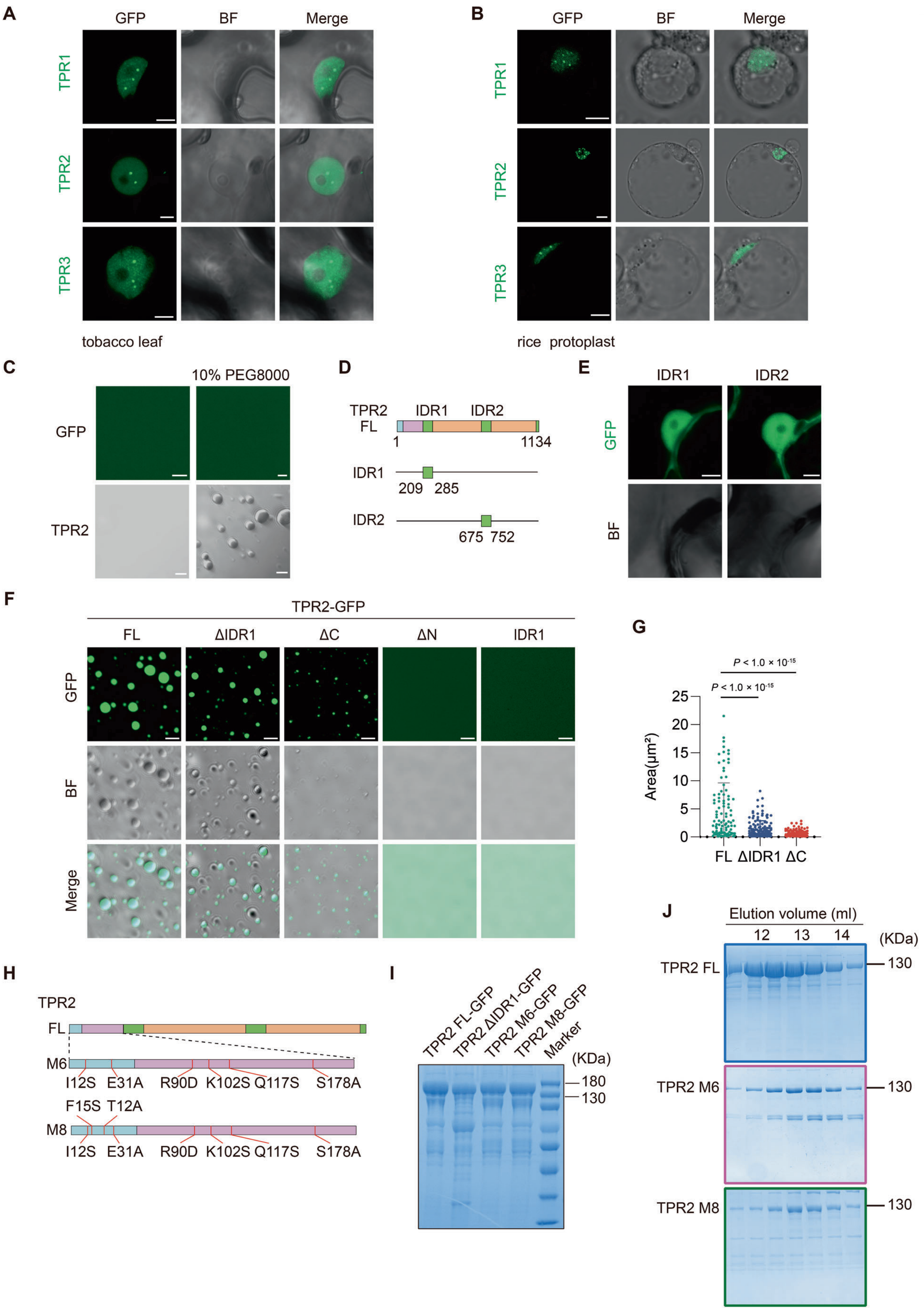
A
GFP
BF
Merge
TPR1
TPR2
TPR3
tobacco leaf
B
GFP
BF
Merge
TPR1
TPR2
TPR3
rice protoplast
C
10% PEG8000
GFP
TPR2
D
TPR2 FL
IDR1
IDR2
1
1134
IDR1
209 285
IDR2
675 752
E
IDR1
IDR2
GFP
BF
F
TPR2-GFP
FL
ΔIDR1
ΔC
ΔN
IDR1
GFP
BF
Merge
G
P < 1.0 × 10-15
P < 1.0 × 10-15
Area(μm²)
25
20
15
10
5
0
FL ΔIDR1 ΔC
H
TPR2
FL
M6
I12S E31A R90D K102S Q117S S178A
F15S T12A
M8
I12S E31A R90D K102S Q117S S178A
I
TPR2 FL-GFP
TPR2 ΔIDR1-GFP
TPR2 M6-GFP
TPR2 M8-GFP
Marker
(KDa)
180
130
J
Elution volume (ml)
12 13 14
(KDa)
TPR2 FL
130
TPR2 M6
130
TPR2 M8
130

 © The Author(s)

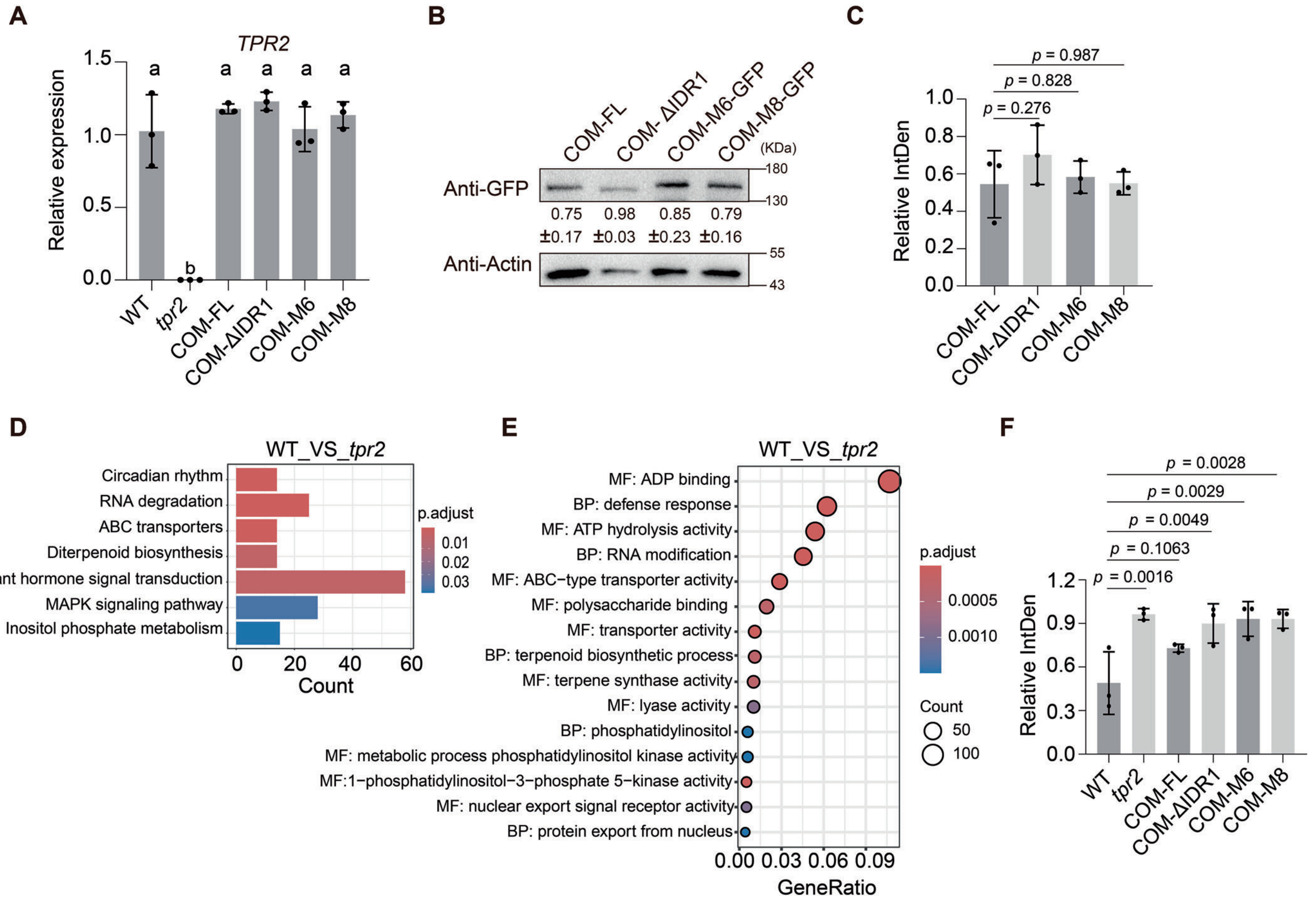

Figure EV2. Phase separation of TPR2 negatively regulates H3K9ac levels at its transcriptionally repressed genes.

(**A**) Expression levels of *TPR2* in WT, *tpr2*, COM-FL, COM-ΔIDR1, COM-M6 and COM-M8 plants. Data were means ± SD ($n = 3$ biological replicates). Different lowercase letters above bars indicate a significant difference at the $P < 0.05$ level by one-way ANOVA with Tukey's multiple-comparisons test. The exact *P* values are listed in Dataset EV4. (**B**) Total proteins extracted from COM-FL, COM-ΔIDR1, COM-M6, and COM-M8 plants were analyzed by Western blot using anti-GFP antibody. Actin served as a loading control. Experiments were independently repeated at least three times with similar results. (**C**) Relative GFP-Tag protein levels in (**B**) were quantified by densitometric analysis and normalized to the loading control. Data were mean ± SD ($n = 3$ biological replicates). *P* values were determined by one-way ANOVA with Dunnett's multiple-comparisons test. (**D**) Kyoto Encyclopedia of Genes and Genomes (KEGG) pathway enrichment analysis of downregulated genes in WT compared to *tpr2*. Enrichment analysis was performed using the clusterProfiler R package with Fisher's exact test and Benjamini–Hochberg false discovery rate (FDR) correction for multiple testing. Pathways with adjusted $P < 0.05$ were considered significantly enriched. (**E**) Gene Ontology (GO) enrichment analysis of downregulated genes in WT compared to *tpr2*. Enrichment analysis was performed using the clusterProfiler R package with Fisher's exact test and Benjamini–Hochberg false discovery rate (FDR) correction for multiple testing. GO terms with adjusted $P < 0.05$ were considered significantly enriched. (**F**) Relative H3K9ac levels in Fig. 4E were quantified by densitometric analysis, and H3 served as a loading control. Data were mean ± SD ($n = 3$ biological replicates). *P* values were determined by one-way ANOVA with Dunnett's multiple-comparisons test. Source data are available online for this figure.

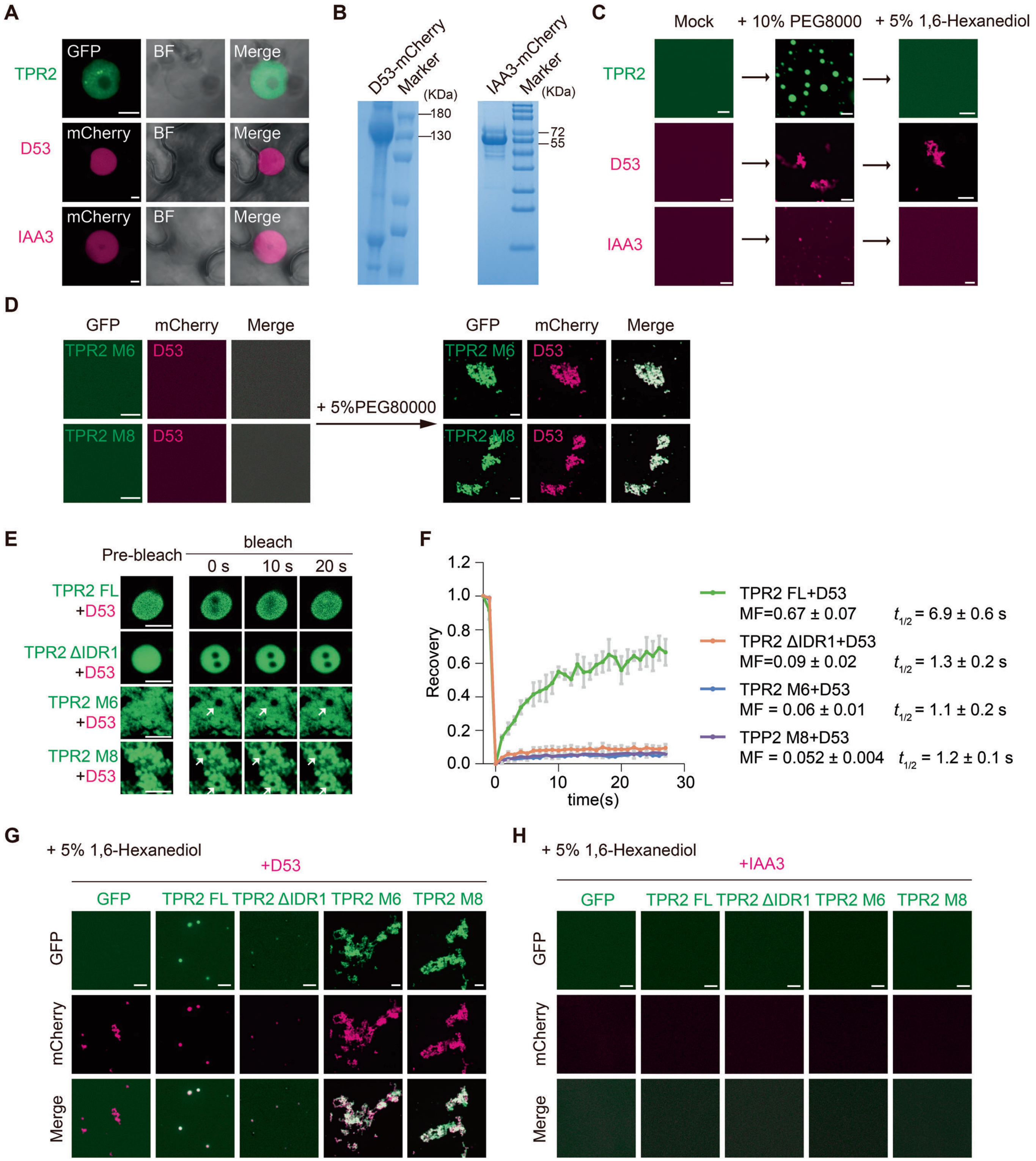
A
TPR2
D53
IAA3
GFP
mCherry
BF
Merge
B
D53-mCherry
Marker
IAA3-mCherry
(KDa)
180
130
72
55
C
Mock
+ 10% PEG8000
+ 5% 1,6-Hexanediol
D
+ 5%PEG80000
TPR2 M6
TPR2 M8
E
Pre-bleach
bleach
0 s
10 s
20 s
TPR2 FL +D53
TPR2 ΔIDR1 +D53
TPR2 M6 +D53
TPR2 M8 +D53
F
Recovery
time(s)
TPR2 FL+D53 MF=0.67 ± 0.07 t1/2 = 6.9 ± 0.6 s
TPR2 ΔIDR1+D53 MF=0.09 ± 0.02 t1/2 = 1.3 ± 0.2 s
TPR2 M6+D53 MF = 0.06 ± 0.01 t1/2 = 1.1 ± 0.2 s
TPP2 M8+D53 MF = 0.052 ± 0.004 t1/2 = 1.2 ± 0.1 s
G
+ 5% 1,6-Hexanediol
+D53
GFP
TPR2 FL
TPR2 ΔIDR1
TPR2 M6
TPR2 M8
mCherry
Merge
H
+ 5% 1,6-Hexanediol
+IAA3

© The Author(s)

Figure EV3. PEG-induced molecular crowding drives the formation of solid-like D53 assemblies with the phase separation-deficient TPR2 mutants.

(**A**) Subcellular localization of TPR2-GFP, D53-mCherry and IAA3-mCherry in tobacco cells. Scale bar = 5 μm. (**B**) SDS–PAGE analysis of D53-mCherry and IAA3-mCherry proteins after affinity purification followed by size-exclusion chromatography. (**C**) Representative confocal images showing purified TPR2-GFP, D53-mCherry, or IAA3-mCherry proteins incubated with 10% PEG8000 followed by the addition of 5% 1,6-hexanediol. In the presence of 10% PEG8000 alone, TPR2-GFP formed large and abundant droplets, D53-mCherry formed condensed structures, and IAA3-mCherry formed small and sparse droplets. Upon addition of 5% 1,6-hexanediol, droplets formed by TPR2-GFP and IAA3-mCherry were completely dissolved, whereas D53-mCherry condensates remained visible. Scale bars = 5 μm. (**D**) Representative confocal images showing purified TPR2M6-GFP and TPR2M8-GFP proteins incubated with D53-mCherry under conditions without PEG8000 (left panels) or with 5% PEG8000 (right panels). Scale bars = 5 μm. (**E**) Representative images of FRAP assays on droplets formed by purified TPR2 FL-GFP and variants (ΔIDR1, M6, and M8) with D53-mCherry in vitro. White arrows indicate the photobleached region. Scale bars = 5 μm. (**F**) FRAP recovery curves of TPR2 FL-GFP and variants (ΔIDR1, M6, and M8) with D53-mCherry droplets. MF and $t_{1/2}$ values are indicated on the graph. (**G**) Representative confocal images showing purified TPR2-FL-GFP and variants (ΔIDR1, M6, and M8) incubated with D53-mCherry in the presence of 10% PEG8000, followed by treatment with 5% 1,6-hexanediol. Under these conditions, droplets formed by TPR2-FL-GFP largely disappeared, while condensates formed by ΔIDR1, M6, and M8 variants remained visible. Scale bars = 5 μm. (**H**) Representative confocal images showing purified TPR2-FL-GFP and variants (ΔIDR1, M6, and M8) incubated with IAA3-mCherry in the presence of 10% PEG8000, followed by treatment with 5% 1,6-hexanediol. Under these conditions, phase-separated droplets formed by all TPR2 variant combinations completely disappeared. Scale bars = 5 μm. Source data are available online for this figure.

© The Author(s)

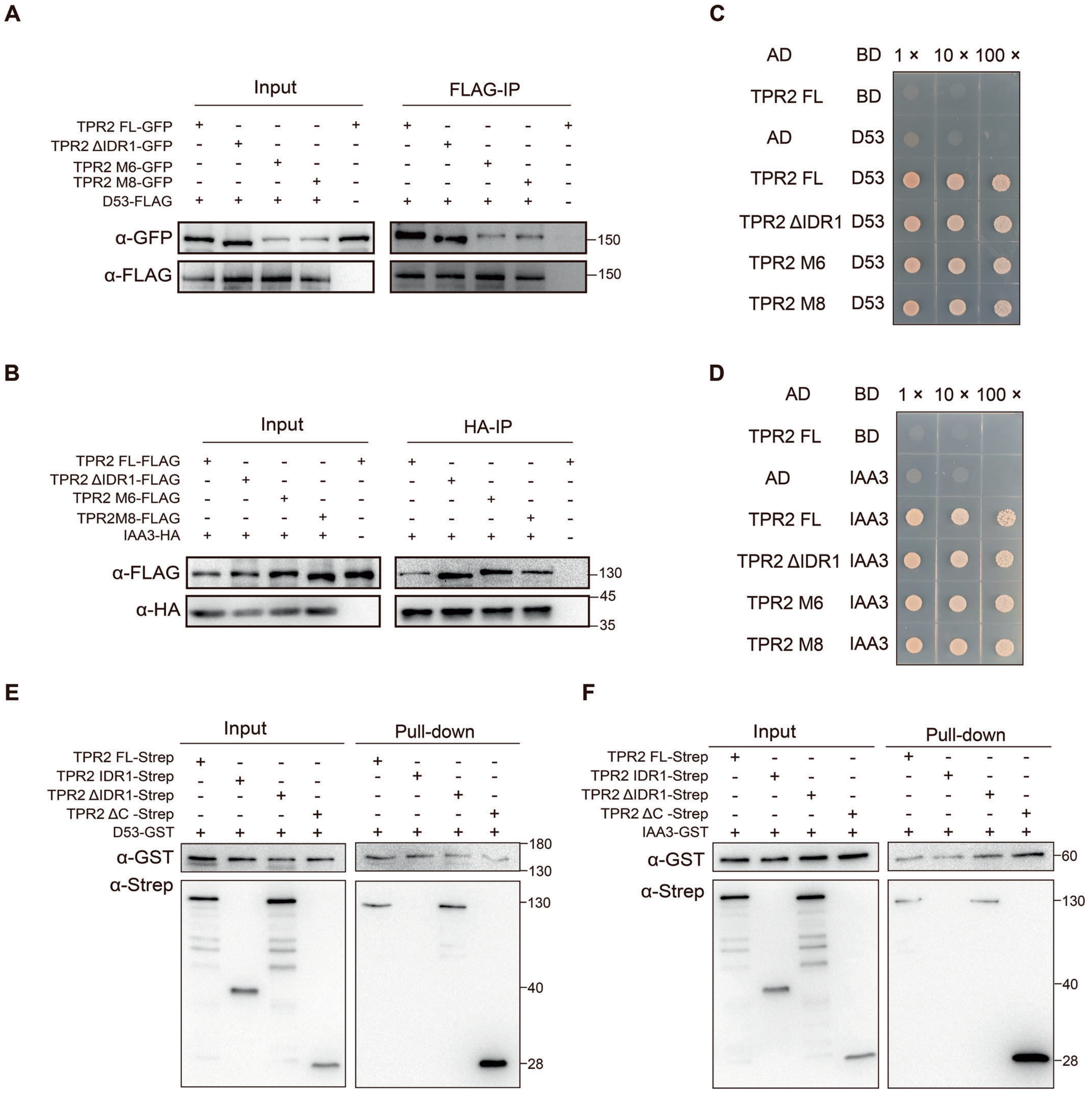

Figure EV4. Both the full-length TPR2 and the phase separation-deficient mutants interact with repressors D53 and IAA3.

(**A**, **B**) Co-IP in rice protoplasts and (**C**, **D**) Yeast two-hybrid assays showing interactions of TPR2 and the phase separation-deficient mutants with D53 and IAA3. (**E**, **F**) GST Pull-down assays indicate that the N-terminal domain of TPR2 is necessary and sufficient for direct interaction with both D53 and IAA3. Source data are available online for this figure.

 © The Author(s)

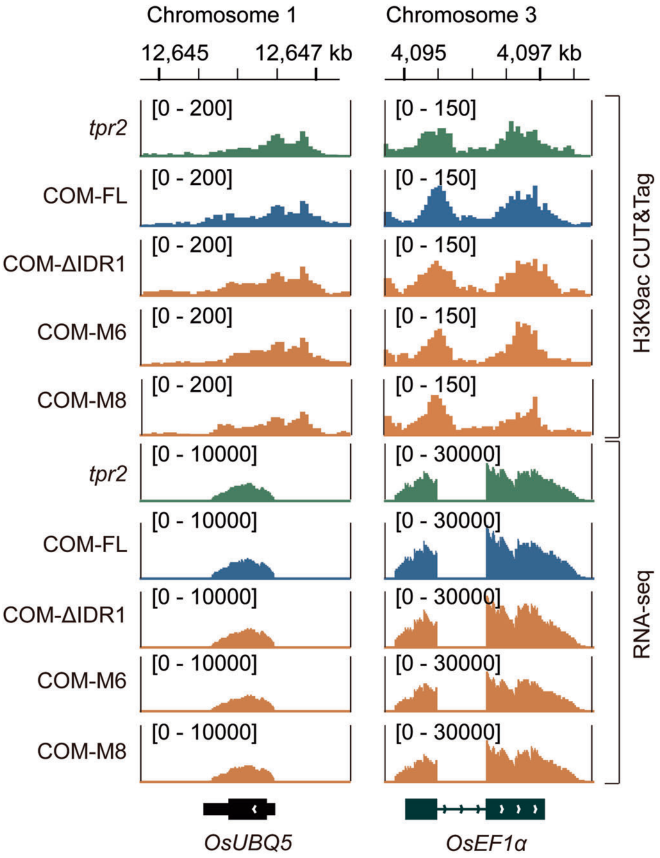

Figure EV5. H3K9 CUT&Tag and RNA-seq signals of negative-control genes.

(**A**) Integrative genome browser visualization of H3K9ac CUT&Tag (top) and RNA-seq signals (bottom) at *OsUBQ5* and *OsEF1a1* in *tpr2*, COM-FL, COM-ΔIDR, COM-M6, and COM-M8 plants.
