# Supplementary material for: The TPR2 corepressor forms condensates with repressors to fine-tune growth and development in rice
Source: EMBO J. 2026 Jul 6;45(15):5330–56. doi: 10.1038/s44318-026-00852-7 (PMC13434662; doi:10.1038/s44318-026-00852-7)
Supplement: Supplementary file 2 [file 44318_2026_852_MOESM1_ESM.pdf]

APPENDIX

The TPR2 corepressor forms condensates with repressors to fine-tune growth and development in rice

Table of Contents

Appendix Figure S1	2	Identification of positive transgenic lines.
Appendix Figure S2	3	Prediction of phase separation propensity of rice TPR proteins
Appendix Figure S3	4	High reproducibility of RNA-seq data and genome-wide distribution of H3K9ac signals.
Appendix Figure S4	5	GFP CUT&Tag of *pTPR2::TPR2-GFP/tpr2* plant.

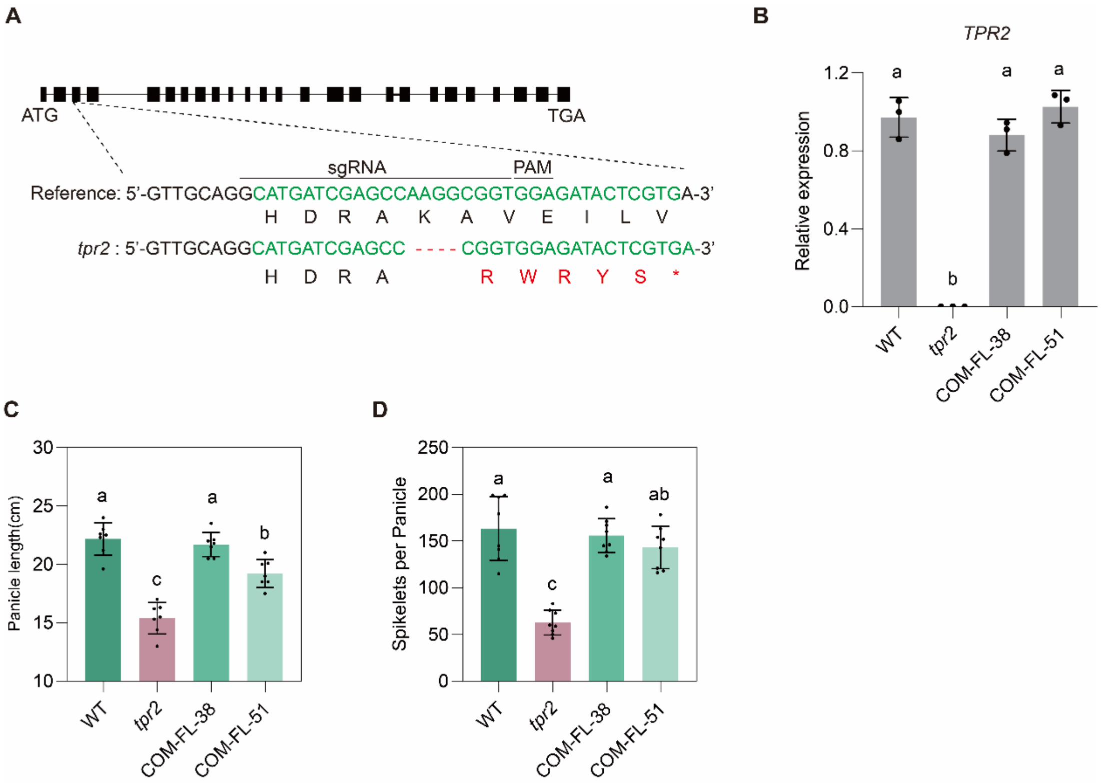

Appendix Figure S1: Identification of positive transgenic lines. (**A**) Schematic presentation of the *TPR2*. Exons are shown as black boxes and introns as lines. sgRNA target sites are indicated in black, with PAM sequences underlined. (**B**) The expression levels of *TPR2* in WT, *tpr2* and complementation lines. Data are means ± SD (n = 3 biological replicates). (**C**-**D**) Comparison of panicle length, spikelet per panicle between ZH11, *tpr2* mutant and complementation lines under normal growth conditions in field. Data are mean ± SD (n = 7, 8 plants). Different lowercase letters above bars indicate a significant difference at the $P < 0.05$ level by one-way ANOVA with Tukey's multiple comparisons test. The exact P values are listed in Table EV4.

A

	catGRANULE	PLAAC	Pscore	ESpritz (DisProt)	Hydropathy	SEG	Charged residue	Phos frequency	DeepPhase	DeepCoil
Score	0.753	-0.010	1.510	0.028	0.466	0.014	0.207	0.000	/	0.000
Rank	0.727	0.910	0.701	0.648	0.414	0.135	0.332	/	/	/

B C

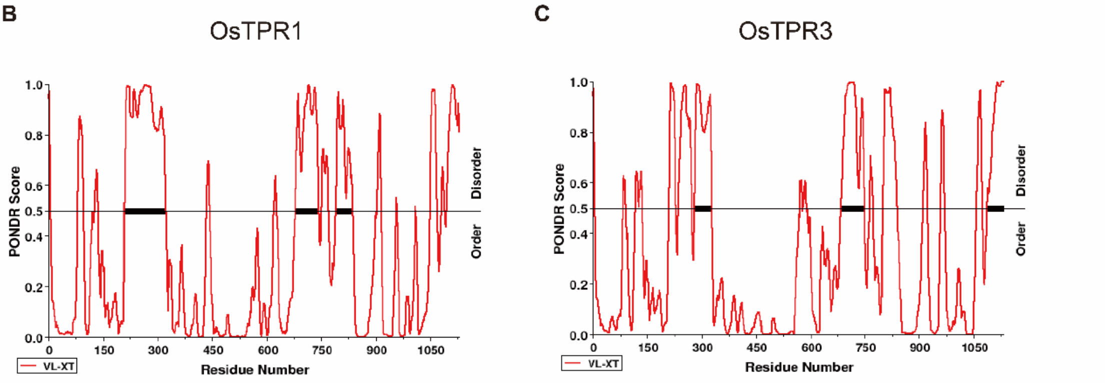

Appendix Figure S2: Prediction of phase separation propensity of rice TPR proteins （**A**） Phase-separation–related sequence properties of OsTPR2 were predicted using the PhaSePred web server (http://predict.phasep.pro/), which integrates multiple predictors including catGRANULE, PLAAC, PScore, ESpritz, DeepPhase and DeepCoil. Scores and proteome-wide ranks for each feature are shown. (**B**, **C**) Prediction of IDRs in OsTPR1 and OsTPR3 using PONDR (www.pondr.com).

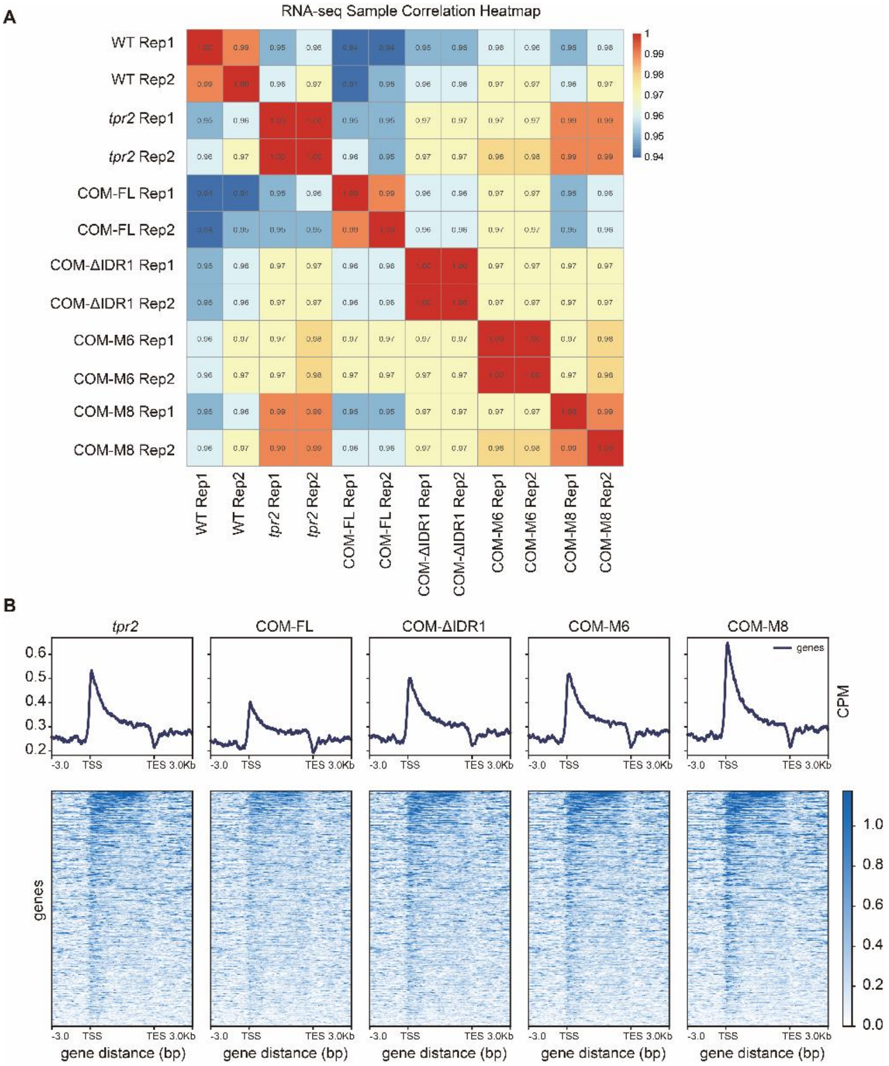

Appendix Figure S3: High reproducibility of RNA-seq data and genome-wide distribution of H3K9ac signals. (**A**) Correlation analysis of RNA-seq samples. (**B**) Normalized H3K9ac CUT&Tag profiles in replicate 2. Top, average signal across genes from 3 kb upstream of TSS to 3 kb downstream of TES. Bottom, heatmap of normalized signal for individual genes across the same regions.

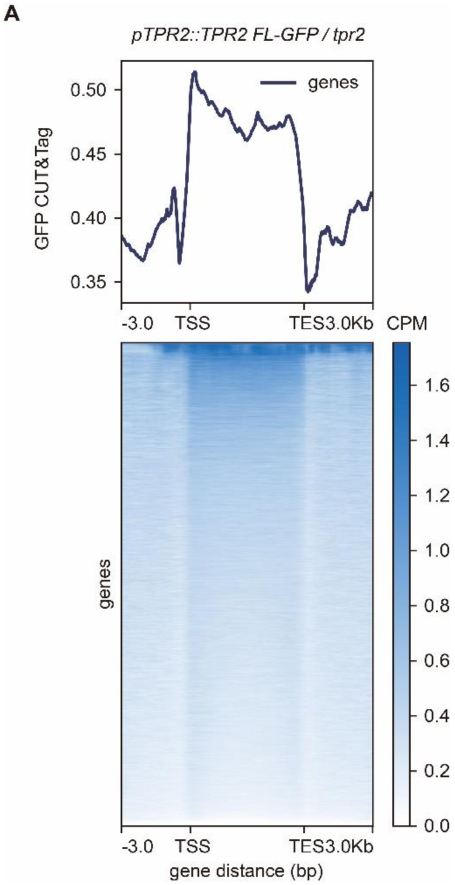

Appendix Figure S4: GFP CUT&Tag of *pTPR2::TPR2-GFP/tpr2* plant. (**A**)Top, average signal across genes from 3 kb upstream of TSS to 3 kb downstream of TES. Bottom, heatmap of normalized signal for individual genes across the same regions.
