# Supplementary material for: The TPR2 corepressor forms condensates with repressors to fine-tune growth and development in rice
Source: EMBO J. 2026 Jul 6;45(15):5330–56. doi: 10.1038/s44318-026-00852-7 (PMC13434662; doi:10.1038/s44318-026-00852-7)
Supplement: Supplementary file 3 [file 44318_2026_852_MOESM2_ESM.pdf]

THE EMBO JOURNAL

The TPR2 corepressor forms condensates with repressors to fine-tune growth and development in rice

Yu Zhang, Bingchen Li, Yu Chen, Jiawei Li, Yao Wang, Tiantian Ye, Ying Ye, Zhiwei Liu, Zhiyue Feng, Stephanie Hutin, Shaoli Zhou, Jianping Guo, Ruihui Zhang, Zhu Liu, Faming Dong, Haiyan Xiong, Chloe Zubieta, Lizhong Xiong, and Xuelei lai

Corresponding author(s): Xuelei lai (xuelei_lai@mail.hzau.edu.cn) , Lizhong Xiong (lizhongx@mail.hzau.edu.cn)

Review Timeline:

Submission Date:	22nd Sep 25
Editorial Decision:	25th Nov 25
Revision Received:	23rd Mar 26
Editorial Decision:	5th May 26
Revision Received:	8th May 26
Accepted:	10th Jun 26

Editor: Cornelius Schneider

Transaction Report:

(Note: With the exception of the correction of typographical or spelling errors that could be a source of ambiguity, letters and reports are not edited. Depending on transfer agreements, referee reports obtained elsewhere may or may not be included in this compilation. Referee reports are anonymous unless the Referee chooses to sign their reports.)

1st Editorial Decision 25th Nov 2025

Dear Prof. Lai,

Thank you for submitting your manuscript for consideration by the EMBO Journal. It has now been seen by two referees. A third referee had agreed to review but did not return their report despite several chasers. We have therefore decided to proceed with the reports of two referees which you can find below.

As you can see from the reports both referees think that the findings are interesting and relevant. However, both referees also voice a large number of major concerns. We think that the referee reports are fair and constructive. Given the referees' positive recommendations, I would like to invite you to submit a revised version of the manuscript, addressing the comments of all three reviewers. I should add that it is EMBO Journal policy to allow only a single round of revision, and acceptance of your manuscript will therefore depend on the completeness of your responses in this revised version. Given that the requested revisions are extensive I think it would be helpful to discuss potential revision experiments by e-mail or videoconferencing whichever you prefer.

When preparing your letter of response to the referees' comments, please bear in mind that this will form part of the Review Process File, and will therefore be available online to the community. For more details on our Transparent Editorial Process, please review our Editorial Policies: https://link.springer.com/partners/embo-press/editorial-policies

We generally allow three months as standard revision time. As a matter of policy, competing manuscripts published during this period will not negatively impact on our assessment of the conceptual advance presented by your study. However, we request that you contact the editor as soon as possible upon publication of any related work, to discuss how to proceed. Should you foresee a problem in meeting this three-month deadline, please let us know in advance and we may be able to grant an extension.

Thank you for the opportunity to consider your work for publication. I look forward to your revision.

Yours sincerely,

Cornelius Schneider, PhD
Editor
The EMBO Journal
c.schneider@embojournal.org

Instructions for preparing your revised manuscript:

Read our guidance for manuscript revisions and related editorial policies: https://link.springer.com/journal/44318/submission-guidelines#cms-Revised-submissions

Please make sure you upload a letter of response to the referees' comments together with the revised manuscript.

Please also check that the title and abstract of the manuscript are brief, yet explicit, even to non-specialists.

When assembling figures, please refer to our figure preparation guideline in order to ensure proper formatting and readability in print as well as on screen:
https://media.springernature.com/original/springer-cms/rest/v1/content/27825798/data/v1

At EMBO Press we ask authors to provide source data for the main manuscript figures. You will receive a separate email with instructions for providing source data with your revised manuscript, including how to upload and organize the files.

IMPORTANT: When you send the revision we will require
- a point-by-point response to the referees' comments, with a detailed description of the changes made (as a word file).
- a word file of the manuscript text.
- individual production quality figure files (one file per figure)
- a complete author checklist
- Expanded View files (replacing Supplementary Information)
- a Reagents and Tools Table as part of the Methods section

Please remember: Digital image enhancement is acceptable practice, as long as it accurately represents the original data and

conforms to community standards. If a figure has been subjected to significant electronic manipulation, this must be noted in the figure legend or in the 'Materials and Methods' section. The editors reserve the right to request original versions of figures and the original images that were used to assemble the figure.

We realize that it is difficult to revise to a specific deadline. In the interest of protecting the conceptual advance provided by the work, we recommend a revision within 3 months (23rd Feb 2026). Please discuss the revision progress ahead of this time with the editor if you require more time to complete the revisions. Use the link below to submit your revision:

https://emboj.msubmit.net/cgi-bin/main.plex

Referee #1:

This manuscript presents a compelling study on the role of phase separation of TOPLESS-RELATED 2 (TPR2) corepressor, focusing on its contribution to transcriptional repression. The central premise, that TPL/TPRs form multivalent hubs via their N-terminal tetramerization and partner binding (e.g., D53, IAA3), and that IDRs guide this process, is sound and supported by functional consequences. The use of a native promoter-driven TPR2-GFP line to observe condensation in vivo is a good support of physiological relevance. The manuscript is generally well-written and structured. However, several key claims require stronger experimental support, primarily concerning the biochemical validation of interactions and the statistical rigor of the imaging data. I have the following specific comments to strengthen the manuscript.

Major Comments
1. A key experiment is missing to distinguish the driver of TPR2 condensation in vivo.
• The finding that the first 208 amino acids are sufficient for phase separation is important. However, to determine if full-lengthTPR2 condensation is driven by homotypic interaction (via the N-terminus) or heterotypic interactions with partners, it is critical to test the phase separation capability of the ΔC fragment in vitro, which is mislabeled as (aa 209-1134). If this fragment does not condense, it would strongly support the model that partner binding is essential for full-length TPR2 condensation.

2. Figure 5 - Phase Separation Assays Need Rigor and Controls: The in vitro condensation data requires significant strengthening to support their interpretation.
• Quantitative analysis of condensation frequency, number, and size across a large number of cells is needed for the TPR2 WT and mutant lines. Representative images alone are not sufficient and could be biased.
• Missing Controls: The figure must include the phase behavior of each individual protein (TPR2, D53, IAA3) alone before showing mixtures as controls. In addition, for cocondensation, a basic control using only a fluorescent protein (in vivo and in vitro) is also necessary to demonstrate that the observed condensation is specific and not passive absorption.
• The data suggest that IAA3 and D53 may interact directly with IDR1. This is a testable hypothesis, and given that the authors have the proteins, a direct in vitro binding assay (e.g., ITC, BLI, SPR, or pull-down) should be performed.
• Protein Quality and Specificity: The aggregation of TPR2 with D53 is difficult to interpret. The D53 protein preparation appears impure ("dirty") in ED Fig 11, and the observed aggregates may be non-specific precipitation from contamination rather than a specific biological interaction. The purification protocol for D53 must be optimized (e.g., adding ion-exchange or size-exclusion chromatography after affinity purification) to ensure data reliability. The intriguing aggregation of M6/M8 with D53 should be investigated in a no-PEG condition to clarify its nature. PEG provides too extreme a condition for these aggregates.

3. Validation and Interpretation of Genomic Data (Figures 4 & 6):
• Figure 4: A critical missing control is an immunoblot to confirm protein levels of the IDR1, M6, and M8 mutants side-by-side with WT and tpr2in the assays shown in Figure 4b and 4d. Furthermore, the analysis should go beyond general consistency in the heatmap; the authors should comment on and explain specific gene hits that are consistent or inconsistent with phase separation behavior between the mutants.
• Figure 6: The model that idr1 null, M6, and M8 phenocopy the full tpr2deletion based on H3K9ac patterns is not fully convincing from the presented data. Each of them looks so different in Fig 6A. The analysis would be strengthened in 6C-F by including a negative control module (unrelated to D53 or IAA3) to demonstrate that the observed effects are specific to the relevant D53 and IAA3 pathways.

4. Experimental Validation of the Model (Figure 7): The proposed structural states of the mutants (M6 as a dimer, M8 as a monomer) are excellent information for the model, but are not experimentally verified. These oligomeric states should be confirmed using biochemical methods such as SEC-MALS or simple SEC-based calibration.

Minor Points:
• Avoid claiming IDR as a direct condensation drive, which can also act as spacers only or modulators, a nuance the authors' own data supports (IDR2 likely as a spacer).
• The authors could easily strengthen the bioinformatic support for the roles of IDR1/IDR2 by using established biophysical prediction tools to analyze their potential as "stickers" or "spacers."

• There are citation errors: ED Fig. 4a,b is incorrectly cited as ED Fig. 3a,b.
• There are labeling inconsistencies: The ΔC construct is defined as aa 209-1134, but is incorrectly labeled as (ΔC, aa 675-752) in line 157. A thorough check for similar errors is recommended.
• Expanding the analysis of TPR2-GFP condensation beyond root tips to other cell types and plant parts would provide valuable physiological insight into its role in development.

Referee #2:

- general summary and opinion about the principle significance of the study, its questions and findings
The authors report that the rice corepressor TPR2 undergoes phase separation (likely LLPS) to regulate transcriptional repression. The authors show that TPR2 forms nuclear condensates through the cooperative action of the IDR1 and a plant-specific N-terminal tetramerisation domain. Phase separation-deficient TPR2 mutant variants failed to rescue developmental defects in tpr2 rice mutants, did not establish proper histone acetylation patterns, or effectively repress selected target genes with pleiotropic effects on general (de-)acetylation patterns. Furthermore, the study reveals that TPR2 co-condenses with repressors D53 and IAA3 to facilitate histone deacetylation and gene silencing in a phase separation-dependent manner. This is a significant and timely contribution to understanding transcriptional repression mechanisms in plants, connecting it to the emerging topic of phase separation. The work is relatively well-executed with appropriate approaches. The identification of a condensate-based mechanism for gene silencing fills an important gap in the relevant plant condensate literature. However, several major concerns regarding the specificity of LLPS, the mechanistic connection to histone deacetylation, and the distinction between LLPS-specific vs. oligomerisation-dependent functions need to be addressed. Authors need to improve the overall clarity of assessment, especially with their biochemical analyses and provide the necessary shortcomings of their observations and approaches. The manuscript has substantial merit and makes important contributions, but the concerns raised, particularly regarding mechanism and causality in the in vitro studies, require additional experimental work or more thorough discussion.
Major strengths
1. The authors provide a good demonstration of a condensate-based mechanism for transcriptional repression in plants (paradigm here of TPR2-likely, other TPRs follow the same pattern), filling an important gap in understanding LLPS roles beyond transcriptional activation. I would, though, recommend that the authors consider and succinctly discuss works on transcript expression regulation by other condensates, such as processing bodies and stress granules.
2. The study combines in vivo and in vitro phase separation assays, structural biology, genetics, genomics, and chromatin analysis to build a narrative for functional condensation.
3. The identification of both IDR1 and the tetramerisation domain as cooperative drivers of phase separation (likely LLPS), with structure-guided mutagenesis (an identified interface from previously published datasets), is relevant.
4. The complementation experiments in rice clearly demonstrate that phase separation capacity correlates with biological function, with graded phenotypes matching those of condensation propensity.
5. The connection between TPR2 condensates, histone deacetylation (H3K9ac), and developmental outcomes provides a relatively meaningful mechanistic insight.

- specific major concerns essential to be addressed to support the conclusions
1. The authors use 10% PEG8000 as a macromolecular crowding agent to induce phase separation in vitro. While this is a commonly used approach, several concerns arise regarding the physiological relevance and experimental design. First, it remains unclear whether the condensates observed under such relatively high PEG concentrations are representative of assemblies that form in vivo. The use of PEG, particularly of high molecular weight, can artificially "push" phase separation by promoting excluded-volume effects rather than mimicking native crowding. I would agree with the authors if they would comment on the viscosity of the nucleus, where TPR2 condensation takes place, but all additions in the buffer need to be justified. Appropriate controls in the absence of PEG should be included to verify the intrinsic propensity of TPR2 to undergo LLPS. Furthermore, the authors should determine a phase diagram to define the saturation concentration (Csat), as this would provide a quantitative framework to interpret the phase behaviour. The chosen PEG concentration (10%) appears arbitrary; justification is needed for this specific value, as lower (e.g., 5%) or higher (e.g., 20%) PEG levels could substantially alter condensate formation dynamics. In addition, more information on the physicochemical composition of the in vitro buffer system is required, particularly regarding salt identity and concentration, which can influence phase separation through salting-out or electrostatic screening effects. The inclusion of GFP alone as a negative control is appropriate; however, GFP differs substantially from TPR2 in both size and biophysical properties. Incorporating additional non-phase-separating proteins of comparable size, charge, and domain organisation would strengthen the argument that the observed condensates are specific to TPR2 rather than a general crowding artifact. It would also be important to contextualise the protein concentrations used in vitro (3-10 μM) relative to the estimated physiological levels of TPR2 in rice nuclei. This could be experimentally assessed through nuclear isolation followed by quantitative western blotting with anti-GFP antibodies, normalised to the number of nuclei. Finally, the use of a GFP fusion tag should be explicitly discussed as a potential limitation. GFP can act as a weak solubilising tag and, due to its size and rigidity, may influence condensate nucleation, stability, and molecular dynamics by increasing hydrodynamic radius and reducing diffusion.
2. Lack of clarity in oligomerisation biochemistry: for example, lines 168-187, 281-290: The M6 and M8 tetramerisation mutants show reduced or absent condensate formation in vivo, but in vitro results are more complex: with D53, the M6 and M8 variants

form 'solid-like condensates/aggregates' rather than dynamic droplets. D53 in the presence of 10% PEG forms solid-like condensates, which confound the composition/properties of the co-condensate of TRP2/D53 (see, for example, the FRAP data and the linear relationship of signal recovery, suggesting an Ostwald-like ripening process, seen later through the increase in size of the co-condensate). Furthermore, the distinction between phase-separated liquid droplets and aggregates is critical but not always clear. How do the authors distinguish between failed LLPS and aggregation? Are there additional material property measurements (e.g., 1,6-hexanediol sensitivity, fusion dynamics)? Note the very slow dynamics reported in the paper (fusion in the order of minutes, which is not discussed). The gel-like properties of D53 alone (line 281-282) complicate interpretation, and additional discussion would be required. Please, provide additional characterisation and/or discussion of (putative) material properties and clarify terminology distinguishing liquid-phase, gel-phase, and aggregated states.
3. Lines 271-275: The TPR2 mutants retain physical interactions with D53 and IAA3 (Co-IP and Y2H) but fail to form condensates and cannot rescue function. Is the functional defect truly due to loss of LLPS, or could it result from: altered interaction dynamics/kinetics not captured by Co-IP? Disrupted higher-order assembly independent of liquid phase properties? Effects on nucleosome binding or other functions? Further discussion is required here.
4. Lines 362-375: The discussion of EAR2 motifs promoting TPR2 oligomerisation raises the key question of whether oligomerisation per se, rather than liquid phase properties, is the key functional feature; I do not see the suggested causality here. The authors should more explicitly address whether they can distinguish LLPS-specific functions from general requirements for oligomerisation or multivalent interactions. This is a fundamental question for the field, but the manuscript creates confusion here. The link between the oligomeric motif and IDR1 interaction is unclear. There is no evidence that oligomer precedes the formation of the condensate. The authors do not attempt any experiment in this direction. I would assume that an opposing scenario would be valid: the oligomeric state is reduced due to the interaction with IDR1 in high Csat (where crowding overtakes low affinity), and hence, the oligomers do not form. Instead, a condensate forms that builds up all the effects described. This intellectual clarity is required as the authors do not address this fundamental query. Furthermore, the authors shall perform SEC analyses or other similar approaches to determine the oligomeric state. This can also be done by purifying the condensates through sedimentation (under conditions promoting condensation). Under the same conditions, the affinity to D53 or IAA3 shall be examined.
5. Lines 234-235: 'Immunoblot analyses revealed increased acetylation at histone H3K9ac, H3K14ac, and H3K27ac in the tpr2 mutant' and lines 71-73: 'TPL/TPRs are known to associate with chromatin-remodelling and histone deacetylase (HDAC) complexes, although whether these interactions are direct or mediated through adaptor proteins remains unclear.' The authors demonstrate that TPR2 condensates are required for histone deacetylation but do not show whether HDACs are enriched in TPR2 condensates, whether the HDAC recruitment/activity is enhanced by LLPS, or the molecular mechanism linking condensate formation to deacetylase activity. They can count a relatively few number of condensates in the nucleoplasm, and the real action may take place in percolation diffraction-limited condensates not visible by confocal microscopy. At least, this interpretation shall be discussed. Perhaps, authors could try to colocalise these complexes in vivo.
6. Lines 361-375: The discussion emphasises plant-specific EAR2 motifs in D53, but the relationship between EAR2-mediated oligomerisation and LLPS is unclear. Is EAR2 required for co-condensation, or is it merely enhancing? Do other EAR-containing repressors also co-condense with TPR2?
7. The authors use only PONDR to predict IDRs. They should extend this part using additional tools for accuracy, and provide the datasets at least as supplemental data. This is because different disorder prediction algorithms use distinct training sets, features, and machine learning approaches, leading to potentially different predictions. Current best practices in the IDR/LLPS field recommend using multiple complementary prediction tools to establish consensus regions. Other tools shall be used to define the molecular grammar of these IDRs, and composition analysis (charge patterning, etc., defined through e.g., PLAAC). Authors could produce a chimeric GFP, testing whether IDR1 and tetramerisation from TPR2 by themselves can drive phase separation in heterologous contexts. Do TPR1 and TPR3 (which also phase separate, Extended Data Fig. 1) have similar IDRs?

Of lesser concern
1. Lines 145-147: 'Whereas the removal of the IDR2 had little effect on TPR2 LLPS, deletion of the IDR1 domain considerably reduced, but did not eliminate, droplet formation.' If IDR2 has no apparent role in LLPS, what is its function? Is it involved in other aspects of TPR2 activity?
2. Lines 116-118, 277: FRAP recovery is shown for TPR2 alone and co-condensates, but quantitative analysis of recovery rates, mobile fractions, and half-times would be valuable. Comparison between different conditions (FL vs. condensate mutants, {plus minus} repressors) is missing. See also comment 2 in the major concerns above for the description of kinetics.
3. Lines 234-235: Multiple acetylation marks increase in tpr2 mutants (H3K9ac, H3K14ac, H3K27ac), but CUT&Tag focuses only on H3K9ac. Are other repressive marks, such as H3K27me3, H3K9me2, affected? This would provide a more complete picture of chromatin state changes-the authors provide only correlative evidence.
4. The manuscript does not address: How quickly condensates form/dissolve, and whether condensate dynamics correlate with gene expression kinetics and how simple developmental or hormonal signals might regulate TPR2 condensation.
5. Extended Data Figure 5a: The schematic of mutation sites would benefit from showing the actual structural context.
6. Figure 6a, b: IGV browser tracks need scale bars and y-axis labels for direct comparison.
7. Lines 428-430: 'Phase separation was induced by supplementing the reaction with 10% (w/v) PEG8000'. In addition, temperature control details?
8. Abstract: acronyms shall be explained, for example, D53, IAA3 and TPR. Furthermore, the presumptive dynamicity has not been shown. Do they dissolve in the presence of hexanediol or other compounds?
9. Line 106: The cleavage of the TPR2 by NAL1 is not fully explained here. Does it also play a role in the condensation process?
10. Line 128: As suggested above, the mobile fraction shall be defined.

11. Line 142 and elsewhere: the IDR acronym has been defined, and the authors still spell it out.
12. Line 281: Please, rephrase as using the word 'under' is a bit confusing and may mean "lower than".
13. The GFP-CUT&Tag shall be explained for non-experts.
14. In general, the proteins purified are of low purity (for example, Extended Data Fig. 5 and 11, for D53 and IAA3). Could the authors comment on this? I agree that sometimes, especially with IDR-containing proteins, high purity cannot be achieved. Did the authors try to tandemly use additional purification steps? This is a certain limitation in the approach they used, and shall be highlighted in the text.
15. Extended Fig. 7: The b and c panels are interesting, but are not sufficiently discussed in the text; I also miss the context here.
16. Some additional clarity on statistics would be required in the Figure Legends. For example, authors report a population size of n=6 for seed setting, but it is not fully clear from how many biological replicates the population size comes from.
17. Fig. 3a: In the M8, panel a, the image quality is either low or the protein has diffused into the nucleolus. Could the authors please comment? Why do they pick regions with different distances from each nucleus for this panel? The same cells shall be used.

Referee #1:

This manuscript presents a compelling study on the role of phase separation of TOPLESS-RELATED 2 (TPR2) corepressor, focusing on its contribution to transcriptional repression. The central premise, that TPL/TPRs form multivalent hubs via their N-terminal tetramerization and partner binding (e.g., D53, IAA3), and that IDRs guide this process, is sound and supported by functional consequences. The use of a native promoter-driven TPR2-GFP line to observe condensation in vivo is a good support of physiological relevance. The manuscript is generally well-written and structured. However, several key claims require stronger experimental support, primarily concerning the biochemical validation of interactions and the statistical rigor of the imaging data. I have the following specific comments to strengthen the manuscript.

Response: We sincerely thank the reviewer for the positive and thoughtful evaluation of our work.

Major Comments

1. A key experiment is missing to distinguish the driver of TPR2 condensation in vivo.

• The finding that the first 208 amino acids are sufficient for phase separation is important. However, to determine if full-lengthTPR2 condensation is driven by homotypic interaction (via the N-terminus) or heterotypic interactions with partners, it is critical to test the phase separation capability of the ΔC fragment in vitro, which is mislabeled as (aa 209-1134). If this fragment does not condense, it would strongly support the model that partner binding is essential for full-length TPR2 condensation.

Response: We thank the reviewer for this insightful suggestion. To directly address whether the N-terminal region of TPR2 is sufficient to drive phase separation, we expressed and purified the ΔC fragment (aa 1-208) and examined its phase separation behavior in vitro. Under molecular crowding conditions (10% PEG8000), the ΔC

fragment was able to form phase-separated droplets, demonstrating that the N-terminal region of TPR2 possesses intrinsic phase separation capacity. However, the droplets formed by TPR2 N-terminal were substantially fewer and smaller than those formed by the full-length protein under the same conditions. Importantly, our previous results showed that deletion of the IDR1 region also significantly compromises the phase separation capacity of TPR2. Taken together, these results suggest that the N-terminal region provides an intrinsic driving force for condensation, while additional regions, including IDR1, contribute cooperatively to promote efficient TPR2 phase separation. These new data have been incorporated into the revised manuscript (**line 154-157**), and present in **Fig. EV1F, G**.

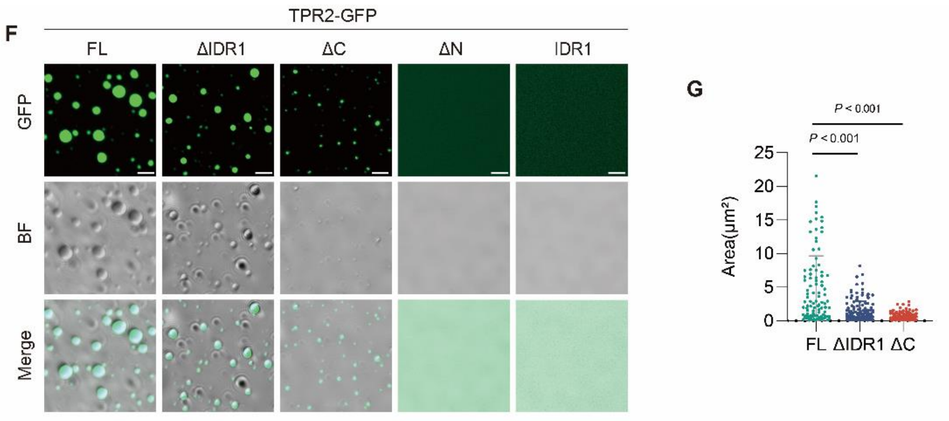

Figure EV1. TPR2 phase separation is jointly mediated by N-terminal tetramerization and IDR1. (**F-G**) *In vitro* phase separation of purified TPR2-FL, ΔIDR1, ΔC, ΔN, and IDR1 proteins in the presence of 10% PEG8000 (**F**). Proteins were labeled with GFP for visualization. Scale bar = 5 μm. (**G**) Quantification of droplet area. Data are mean ± SD (n = 3 fields per group); P values were determined by one-way ANOVA with Dunnett's multiple-comparisons test.

However, we have also observed that TPR2 heterotypic interactions with binding repressors IAA3 and D53 can dramatically increase its phase separation capacity **(Fig. 5A-E)**. Thus, our data support a model in which TPR2 condensation is driven by multivalent homotypic interactions involving both the N-terminal region and IDR1, and that heterotypic interactions with repressors further enhance the phase separation *in vivo*.

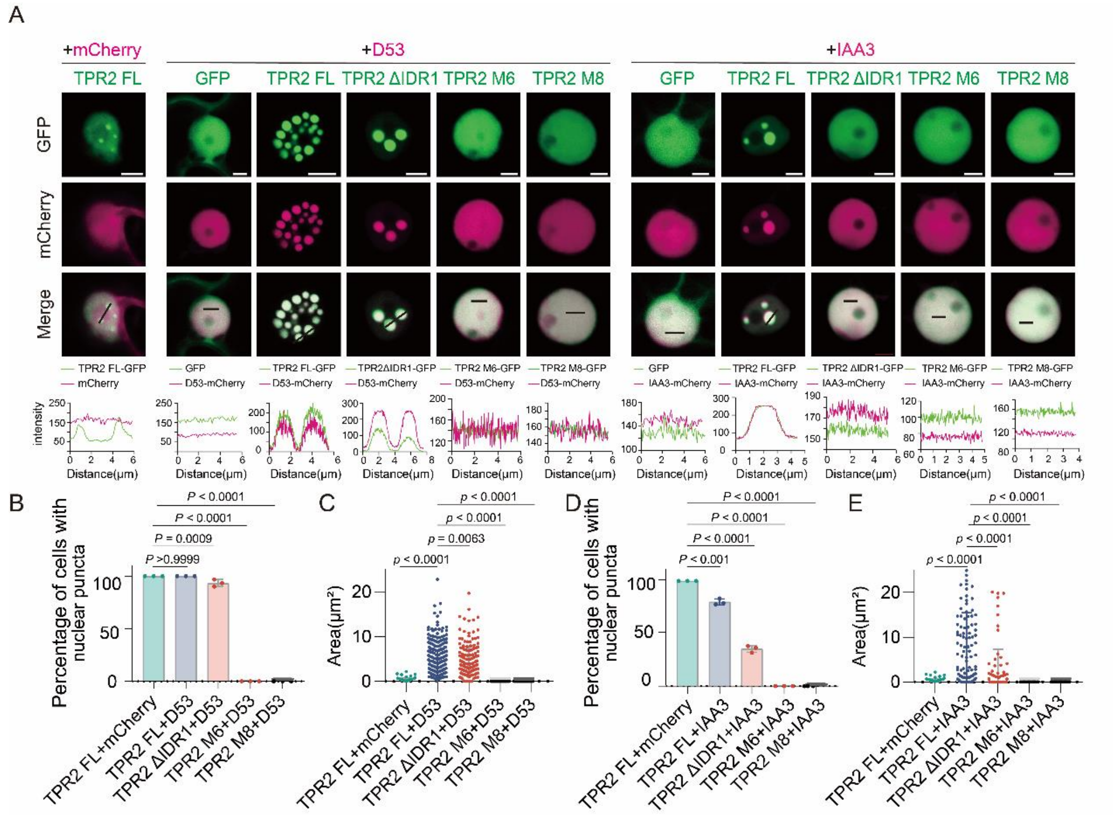

Figure 5. TPR2 condensate with repressors *in vivo* and *in vitro*. (**A**) Colocalization of TPR2-GFP variants with mCherry-tagged repressors. Representative confocal images (top panels) and quantification (bottom panels) showing TPR2-GFP variants (FL, ΔIDR1, M6, M8; green) co-expressed with mCherry alone (negative control), D53-mCherry, or IAA3-mCherry (magenta). Scale bars = 5 μm. (**B**, **C**) Quantitative analysis of nuclear puncta formation with D53. (**B**) Percentage of cells showing nuclear puncta upon co-expression of TPR2 variants with D53. (**C**) Size of individual nuclear puncta in cells co-expressing TPR2 variants with D53. (**D**, **E**) Quantitative analysis of nuclear puncta formation with IAA3. (**D**) Percentage of cells showing nuclear puncta upon co-expression of TPR2 variants with IAA3. (**E**) Size of individual nuclear puncta in cells co-expressing TPR2 variants with IAA3. (**B**-**E**) Data are mean ± SD (n = 3 biologically independent samples; 20 transfected cells per group); P values were determined by one-way ANOVA with Dunnett's multiple-comparisons test.

2. Figure 5 - Phase Separation Assays Need Rigor and Controls: The in vitro condensation data requires significant strengthening to support their interpretation.

• Quantitative analysis of condensation frequency, number, and size across a large number of cells is needed for the TPR2 WT and mutant lines. Representative images alone are not sufficient and could be biased.

Response: we thank the reviewer for this important suggestion. To strengthen the analysis and avoid potential bias from representative images, we performed quantitative analysis of condensation across a large number of cells for each condition. Specifically,

for TPR2 WT and mutant constructs co-expressed with D53 or IAA3, we quantified (i) the percentage of cells exhibiting condensates, as well as (ii) the number and size of condensates per cell in cells displaying phase separation. For each condition, 60 cells were analyzed.

This quantitative analysis revealed that, compared with TPR2 WT, the mutant forms of TPR2 showed a pronounced reduction in condensate formation when co-expressed with D53 or IAA3. In particular, the number of condensates per cell and the average condensate size were significantly decreased in the mutant backgrounds. These results quantitatively support the conclusion that the tested mutations impair the phase separation capacity of TPR2 in vivo.

The quantitative analyses have been added to the revised manuscript (**line 287-293** and are presented in **Fig. 5B – E**.

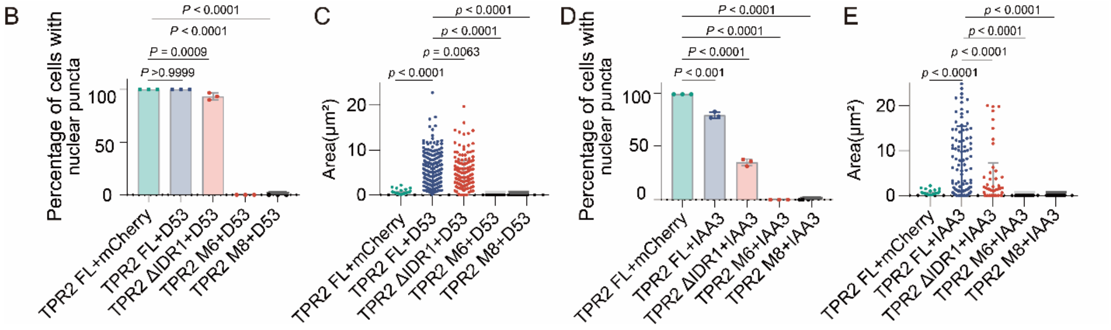

Figure 5. TPR2 condensate with repressors *in vivo* and *in vitro*. (**B**, **C**) Quantitative analysis of nuclear puncta formation with D53. (**B**) Percentage of cells showing nuclear puncta upon co-expression of TPR2 variants with D53. (**C**) Size of individual nuclear puncta in cells co-expressing TPR2 variants with D53. (**D**, **E**) Quantitative analysis of nuclear puncta formation with IAA3. (**D**) Percentage of cells showing nuclear puncta upon co-expression of TPR2 variants with IAA3. (**E**) Size of individual nuclear puncta in cells co-expressing TPR2 variants with IAA3. (**B-E**) Data are mean ± SD (*n* = 3 biologically independent samples; 20 transfected cells per group); *P* values were determined by one-way ANOVA with Dunnett's multiple-comparisons test.

• Missing Controls: The Figure must include the phase behavior of each individual protein (TPR2, D53, IAA3) alone before showing mixtures as controls. In addition, for co-condensation, a basic control using only a fluorescent protein (in vivo and in vitro) is also necessary to demonstrate that the observed condensation is specific and not passive absorption.

Response: we thank the reviewer for highlighting the importance of appropriate controls. In the revised manuscript, we have added the requested controls to rigorously assess the phase behavior of the individual proteins and to exclude the possibility that the observed condensates arise from nonspecific recruitment of fluorescent proteins.

First, to examine the intrinsic phase behavior of each component, we analyzed the localization of TPR2-GFP, D53-mCherry, and IAA3-mCherry expressed individually *in vivo*. In addition, we tested the phase separation behavior of the purified proteins individually *in vitro* under molecular crowding conditions (PEG).

These new data have been incorporated into the revised manuscript (**line 305-310**), and present in **Fig. EV3A, C.**

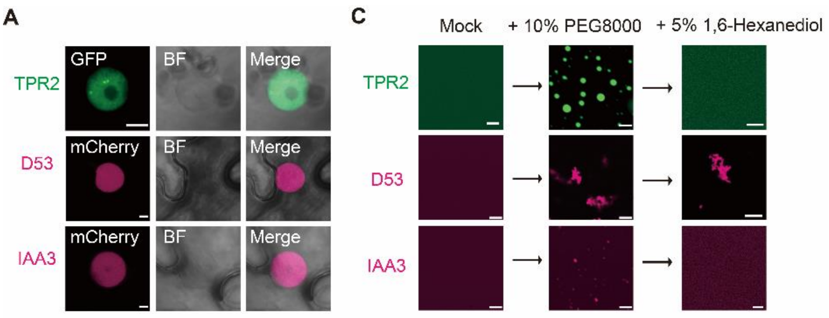

Figure EV3. PEG-induced molecular crowding drives the formation of solid-like D53 assemblies with the phase separation deficient TPR2 mutants. (**A**) Subcellular localization of TPR2-GFP, D53-mCherry and IAA3-mCherry in tobacco cells. Scale bar, 5 µm. (**C**) Representative confocal images showing purified TPR2-GFP, D53-mCherry, or IAA3-mCherry proteins incubated with 10% PEG8000 followed by addition of 5% 1,6-hexanediol. In the presence of 10% PEG8000 alone, TPR2-GFP formed large and abundant droplets, D53-mCherry formed aggregates, and IAA3-mCherry formed small and sparse droplets. Upon addition of 5% 1,6-hexanediol, droplets formed by TPR2-GFP and IAA3-mCherry were completely dissolved, whereas D53-mCherry condensates remained visible. Scale bars = 5 µm.

Second, to control for potential nonspecific recruitment of fluorescent proteins during co-condensation, we included fluorescent protein controls both *in vivo* and *in vitro*. *In vivo*, we co-expressed TPR2-GFP with mCherry, as well as D53-mCherry or IAA3-mCherry with GFP, and the corresponding images have been added to **Fig. 5A**. *In vitro*, we examined mixtures of purified D53 with GFP and purified IAA3 with GFP, and these results are shown in **Fig. 5J** and **Fig. 5K**, respectively.

Together, these controls demonstrate that the observed condensates arise from specific interactions among TPR2 and its partners rather than nonspecific partitioning of fluorescent proteins.

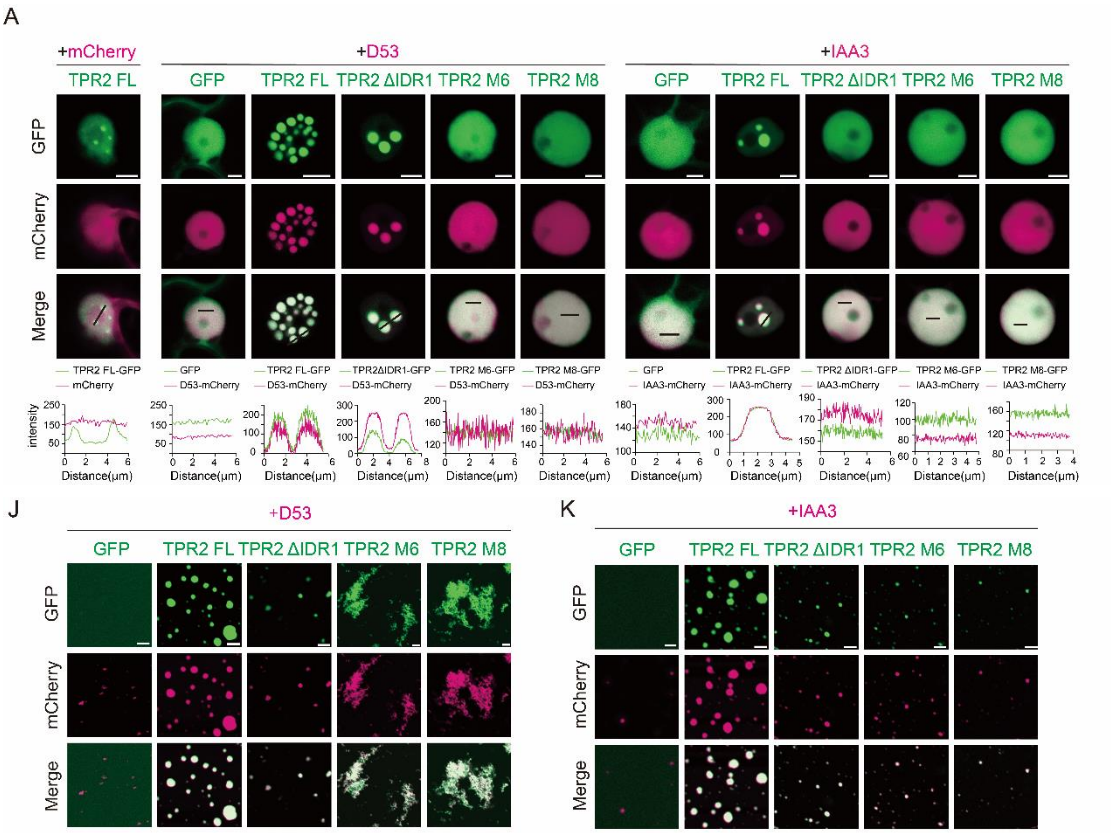

Figure 5. TPR2 condensate with repressors *in vivo* and *in vitro*. (**A**) Colocalization of TPR2-GFP variants with mCherry-tagged repressors. Representative confocal images (top panels) and quantification (bottom panels) showing TPR2-GFP variants (FL, ΔIDR1, M6, M8; green) co-expressed with mCherry alone (negative control), D53-mCherry, or IAA3-mCherry (magenta). Scale bars = 5 μm. **(J, K)** Representative images showing droplet formation of GFP or TPR2-GFP variants (FL, ΔIDR1, M6, M8) with D53-mCherry (**J**) or IAA3-mCherry (**K**) in the presence of 10% PEG8000. Scale bars = 5 μm.

• The data suggest that IAA3 and D53 may interact directly with IDR1. This is a testable hypothesis, and given that the authors have the proteins, a direct in vitro binding assay (e.g., ITC, BLI, SPR, or pull-down) should be performed.

Response: we thank the reviewer for this valuable suggestion. Previous studies have reported that TPR2 primarily interacts with transcriptional repressors through its N-terminal region. To directly test whether IDR1 contributes to binding with D53 or IAA3, we performed in vitro pull-down assays using purified proteins.

Our results show that the isolated IDR1 fragment of TPR2 does not interact with either D53 or IAA3. Consistently, a TPR2ΔIDR1 mutant retains the ability to bind D53 and IAA3, indicating that IDR1 is not required for these interactions. In contrast, the N-terminal fragment of TPR2 (aa 1–208) was sufficient to interact with both D53 and IAA3 *in vitro*.

Together, these results demonstrate that the interaction between TPR2 and these transcriptional repressors is primarily mediated by the N-terminal region, rather than IDR1. These findings are consistent with previous reports and clarify that the contribution of IDR1 to TPR2 condensation is unlikely to arise from direct binding to D53 or IAA3.

The results of these pull-down assays have been added to the revised manuscript and are presented in **Fig. EV4E, F**.

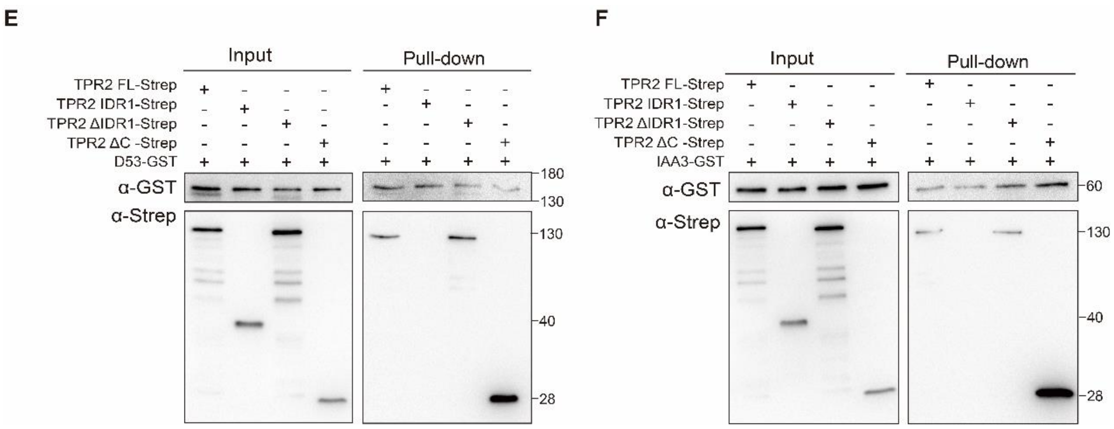

Figure EV4. (E-F) GST Pull-down assays indicate that the N-terminal domain of TPR2 is necessary and sufficient for direct interaction with both D53 and IAA3.

• Protein Quality and Specificity: The aggregation of TPR2 with D53 is difficult to interpret. The D53 protein preparation appears impure ("dirty") in ED Fig. 11, and the observed aggregates may be non-specific precipitation from contamination rather than a specific biological interaction. The purification protocol for D53 must be optimized (e.g., adding ion-exchange or size-exclusion chromatography after affinity purification) to ensure data reliability. The intriguing aggregation of M6/M8 with D53 should be investigated in a no-PEG condition to clarify its nature. PEG provides too extreme a

condition for these aggregates.

Response: we thank the reviewer for raising this important concern regarding protein quality and the interpretation of the aggregation behavior. To improve the purity of the D53 preparation, we have further optimized the purification procedures. In addition to affinity purification, we introduced size-exclusion chromatography (SEC) as an additional purification step, which yielded a substantially cleaner D53-mCherry preparation, as shown in **Fig. EV3B**. The detailed protein purification method is described in the **Protein expression and purification** section of the **Methods (line 483-488)**.

To further clarify the nature of the observed aggregation, we performed additional control experiments. First, when M6/M8 and D53 were incubated in the absence of PEG, no condensate or aggregation was observed, indicating that molecular crowding is required for this behavior. Second, to test whether the aggregation depends on excessively strong crowding conditions, we reduced the PEG concentration from 10% to 5%, and the aggregation phenotype was still observed (**Fig. EV3D**), suggesting that the phenomenon is not restricted to extreme crowding conditions.

Importantly, we also observed that D53 alone forms aggregates upon PEG addition (**Fig. EV3C**). This result suggests that the aggregation observed in mixtures of M6/M8 and D53 is likely related to the intrinsic aggregation propensity of D53 under crowding conditions rather than arising from nonspecific contamination.

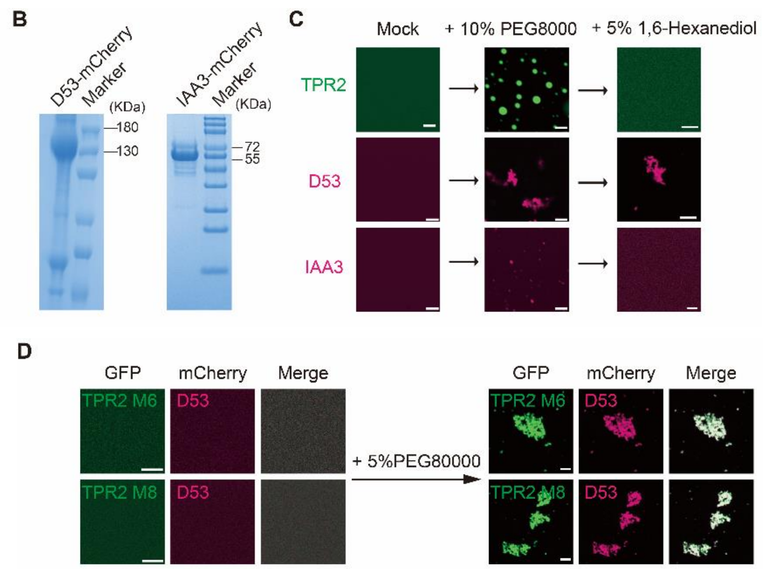

Figure EV3. PEG-induced molecular crowding drives the formation of solid-like D53 assemblies with the phase separation deficient TPR2 mutants. (**B**) SDS-PAGE analysis of D53-mCherry and IAA3-mCherry proteins after affinity purification followed by size-exclusion chromatography. (**C**) Representative confocal images showing purified TPR2-GFP, D53-mCherry, or IAA3-mCherry proteins incubated with 10% PEG8000 followed by addition of 5% 1,6-hexanediol. In the presence of 10% PEG8000 alone, TPR2-GFP formed large and abundant droplets, D53-mCherry formed aggregates, and IAA3-mCherry formed small and sparse droplets. Upon addition of 5% 1,6-hexanediol, droplets formed by TPR2-GFP and IAA3-mCherry were completely dissolved, whereas D53-mCherry condensates remained visible. Scale bars = 5 µm. (**D**) Representative confocal images showing purified TPR2M6-GFP and TPR2M8-GFP proteins incubated with D53-mCherry under conditions without PEG8000 (left panels) or with 5% PEG8000 (right panels).

3. Validation and Interpretation of Genomic Data (Figures 4 & 6):

• Figure 4: A critical missing control is an immunoblot to confirm protein levels of the IDR1, M6, and M8 mutants side-by-side with WT and *tpr2* in the assays shown in Figure 4b and 4d. Furthermore, the analysis should go beyond general consistency in the heatmap; the authors should comment on and explain specific gene hits that are consistent or inconsistent with phase separation behavior between the mutants.

Response: we thank the reviewer for this important suggestion. Because a specific antibody against TPR2 is not available, we initially quantified *TPR2* transcript levels in the complementation lines to ensure comparable expression among the different constructs. These analyses showed no significant differences in *TPR2* expression among *ZH11, COM-FL, COM-ΔIDR1, COM-M6*, and *COM-M8* lines.

To further address the reviewer's concern at the protein level, we have now performed

immunoblot analysis using an anti-GFP antibody to detect the GFP-tagged TPR2 proteins in the complementation lines, with Actin as a loading control. The results show that the protein levels of TPR2-FL, TPR2-ΔIDR1, TPR2-M6, and TPR2-M8 are comparable across the lines, indicating that the observed phenotypic and transcriptional differences are unlikely to arise from differences in protein abundance. These new data have been incorporated into the revised manuscript (**line 209-211**), and present in **Fig. EV2A-C.**

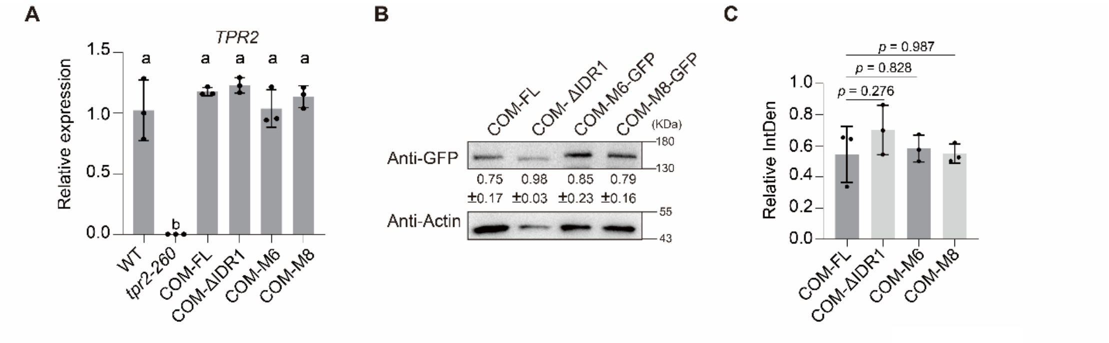

Figure EV2. Phase separation of TPR2 negatively regulates H3K9ac levels at its transcriptionally repressed genes. (**A**) Expression levels of *TPR2* in WT, *tpr2*, COM-FL, COM-ΔIDR1, COM-M6 and COM-M8 plants. Data are means ± SD ($n = 3$ biological replicates). Different lowercase letters above bars indicate a significant difference at the $P < 0.05$ level by one-way ANOVA with Tukey's multiple comparisons test. (**B**) Total proteins extracted from COM-FL, COM-ΔIDR1, COM-M6 and COM-M8 plants were analyzed by Western blot using anti-GFP antibody. Actin served as a loading control. Experiments were independently repeated at least three times with similar results. (C) Relative protein levels in (B) were quantified by densitometric analysis and normalized to the loading control. Data are mean ± SD ($n = 3$ biological replicates). *P* values were determined by one-way ANOVA with Dunnett's multiple-comparisons test.

Regarding the reviewer's second point on specific gene hits. In the original manuscript, we highlighted six well-characterized TPR2-associated target genes, including *OsTEM1* (Osnato et al., 2020), *OsA7* (Hu et al., 2022), *OsLUX* (Cai et al., 2022), *OsERF1* (Hu et al., 2008), *SAUR39* (Kant et al., 2009), and *OsJAR2* (Jain et al., 2006) in Figure 6, to illustrate how these loci may be co-regulated by TPR2 and its associated repressors. Following the reviewer's suggestion, we have now added the corresponding RNA-seq data for these representative genes, which show trends consistent with the repressive function of TPR2 and the impaired activity of its LLPS-deficient variants. These new data are now presented in **Fig. 6B**.

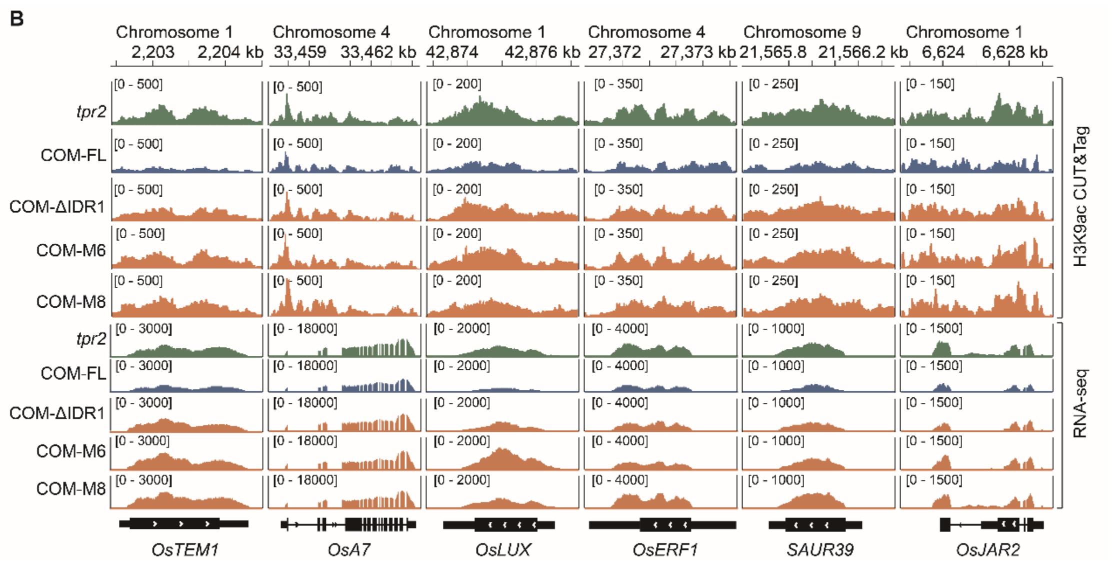

Figure 6. Phase separation of TPR2 promotes transcription suppression with repressors. (**B**) Integrative genome browser visualization of H3K9ac CUT&Tag signals and RNA-seq signals at *TME1*, *A7*, *LUX*, *ERF1*, *SAUR39*, and *JAR2* in *tpr2* and *pTPR2::TPR2FL-GFP/tpr2* (COM-FL), *pTPR2::TPR2ΔIDR1-GFP/tpr2* (COM-ΔIDR), *pTPR2::TPR2M6-GFP/tpr2* (COM-M6), and *pTPR2::TPR2M8-GFP/tpr2* (COM-M8) plants. Data from 20-day-old rice seedlings.

• Figure 6: The model that idr1 null, M6, and M8 phenocopy the full *tpr2* deletion based on H3K9ac patterns is not fully convincing from the presented data. Each of them looks so different in Fig. 6A. The analysis would be strengthened in 6C-F by including a negative control module (unrelated to D53 or IAA3) to demonstrate that the observed effects are specific to the relevant D53 and IAA3 pathways.

Response: We thank the reviewer for this very important comment. We fully agree that inclusion of a negative control module unrelated to the D53 or IAA3 pathways is essential for a more rigorous interpretation of the reporter assays.

Following this suggestion, we selected the commonly used reference genes *OsUBQ5* and *OsEF1α* for further analysis. Consistent with their expected behavior as negative controls, our genome-wide H3K9ac profiling showed no obvious differences at the *OsUBQ5* and *OsEF1α* loci among *tpr2*, COM-FL, COM-ΔIDR1, COM-M6 and COM-M8 plants. We therefore proceeded to test these promoters in the rice protoplast reporter system. However, unexpectedly, co-expression of TPR2 together with either the IPA1-D53 or the ARF25-IAA3 module reduced reporter expression driven by both the

OsUBQ5 and *OsEF1α* promoters, even though these promoters do not contain known IPA1- or ARF25-binding sites. In both cases, repression was partially alleviated when LLPS-impaired TPR2 variants were used, similar to what we observed for the six potential target genes (**Response Figure 1**). These results suggest that, under transient overexpression conditions, TPR2 likely acts as a broadly acting corepressor in protoplasts and can suppress reporter activity independently of the specific IPA1-D53 or ARF25-IAA3 regulatory context.

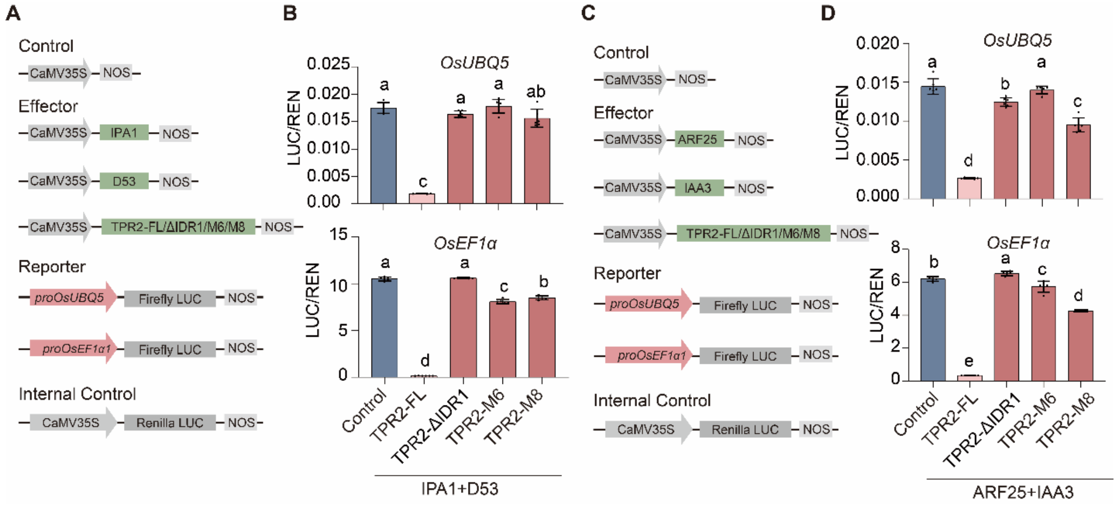

Response Figure 1. Dual-luciferase assay showing transcriptional repression by TPR2 full-length (FL) and variants (ΔIDR1, M6, M8) on negative control genes. (**A**, **C**) Schematics diagrams of constructs used in dual-LUC assay. (**B**, **D**) Effects on *OsUBQ5* and *OsEF1α* via the IPA1-D53 module (**B**) and the ARF25-IAA3 module. Reporters and effectors were co-expressed in rice protoplasts; relative LUC activities were normalized to REN activities (LUC/REN). Data are mean ± SD (n = 3 biological replicates, 2 technical replicates each). P values were determined by one-way ANOVA with Tukey's multiple-comparisons test.

These observations led us to conclude that the protoplast reporter system is not an ideal assay for validating pathway specificity in this case. In addition, because TPR2 is proposed to function through recruitment of histone deacetylation machinery, the validity of this system would depend on proper chromatinization of the transient reporter constructs, including nucleosome assembly on both the promoter and reporter regions, which is difficult to verify in protoplasts. We therefore consider this system suboptimal for assessing the specificity of epigenetic repression.

For this reason, we have removed the protoplast reporter data from the revised

manuscript and no longer rely on this assay to support our conclusions. In the revised version, we have included *OsUBQ5* and *OsEF1α* as negative controls in the genome-wide H3K9ac profiling analysis (**Figure EV5A**), and expression profiles of the potential target genes of the IPA1-D53-TPR2 and ARF5-IAA3-TPR2 pathway (**Figure 6**). In addition, we performed immunoblot analysis of H3K9ac across the mutant and complementation lines. As shown in **Figure 4E**, H3K9ac levels are markedly elevated in *tpr2*, restored in COM-FL, and remain high in the LLPS-deficient lines COM-ΔIDR1, COM-M6, and COM-M8, in strong agreement with the genome-wide H3K9ac profiling data. These new data, together with the H3K9ac profiles at *OsUBQ5* and *OsEF1α*, have now been incorporated into **Figure 4E** and **Figure EV5A**.

Taken together, these findings indicate that the transient protoplast reporter assay is not an appropriate system for assessing the specificity of TPR2-dependent repression in this context. We therefore revised our analysis to focus on the datasets that are most informative and internally consistent for this question, including genome-wide H3K9ac profiling (CUT&Tag), TPR2 binding data (CUT&Tag), target-gene expression analyses (RNA-seq), and H3K9ac immunoblotting across *tpr2*, COM-FL, COM-ΔIDR1, COM-M6, and COM-M8 plants. These independent but mutually supportive datasets consistently show that impaired TPR2 condensation is associated with chromatin states and expression at a subset of potential target genes linked to the D53 and IAA3 pathways. Accordingly, we have rewritten the text corresponding to **Figure 6**.

Figure 4

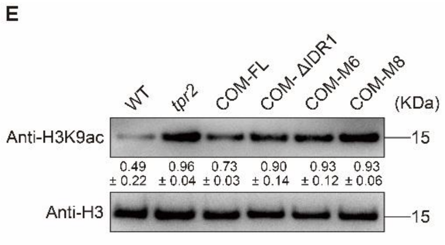

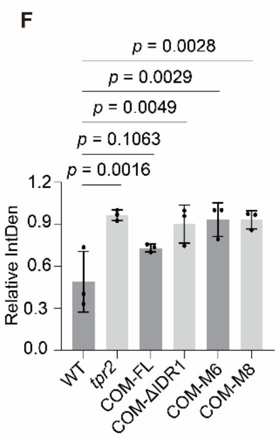

Figure EV2

Figure 4. Phase separation of TPR2 negatively regulates H3K9ac levels at its transcriptionally repressed genes. (**E**) Immunoblot analysis of H3K9 acetylation levels in WT, *tpr2*, COM-ΔIDR1, COM-M6 and COM-M8 lines. Histone extracts from WT, tpr2, COM-ΔIDR1, COM-M6, and COM-M8 seedlings were subjected to immunoblotting using anti-acetyl-H3K9 antibody. Total H3 was used as a loading control. Experiments were

independently repeated at least three times with similar results.

Figure EV2. Phase separation of TPR2 negatively regulates H3K9ac levels at its transcriptionally repressed genes. (F) Relative protein levels in fig. 4E were quantified by densitometric analysis and normalized to the loading control. Data are mean ± SD (*n* = 3 biological replicates). *P* values were determined by one-way ANOVA with Dunnett's multiple-comparisons test.

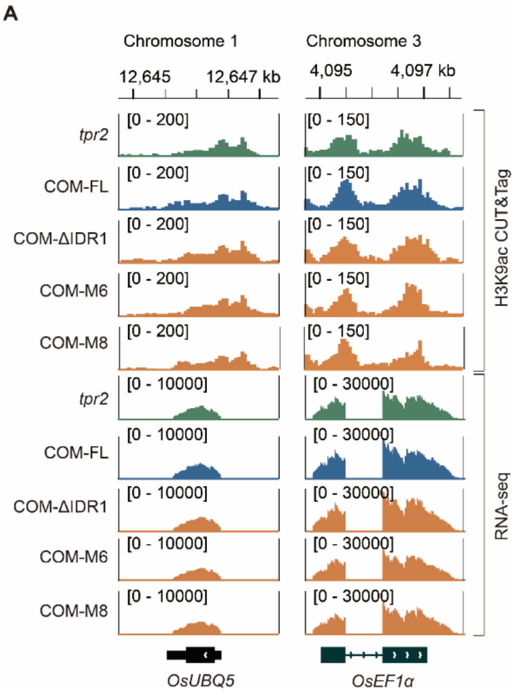

Figure EV5. H3K9 CUT&Tag and RNA-seq signals of negative control genes. (**A**) Integrative genome browser visualization of H3K9ac CUT&Tag (top) and RNA-seq signals (bottom) at *OsUBQ5* and *OsEF1α1* in *tpr2*, COM-FL, COM-ΔIDR, COM-M6, and COM-M8 plants.

4. Experimental Validation of the Model (Figure 7): The proposed structural states of the mutants (M6 as a dimer, M8 as a monomer) are excellent information for the model, but are not experimentally verified. These oligomeric states should be confirmed using biochemical methods such as SEC-MALS or simple SEC-based calibration.

Response: we thank the reviewer for this important suggestion. To experimentally validate the oligomeric states proposed in our model, we performed size-exclusion chromatography (SEC) for purified TPR2-FL, TPR2-M6, and TPR2-M8 proteins. The elution profiles indicate that both M6 and M8 mutants elute at positions corresponding approximately to dimeric species, whereas TPR2-FL elutes at its expected position for a tetrameric complex. These results provide biochemical support for the model and confirm that the M6 and M8 mutations alter the oligomeric assembly of TPR2 as

predicted.

We note, however, that because both M6 and M8 contain substitutions affecting the two tetramerization interfaces, SEC analysis alone does not allow us to determine which interface is retained in each mutant, nor does it exclude the possibility that multiple dimeric species coexist in the samples. Accordingly, we have revised the model so that both M6 and M8 are represented as dimers, which more accurately reflects the SEC data.

These new data have been incorporated into the revised manuscript (**line 182-189**), and present in **Fig. 2E** and **Fig. EV1 J.**

Figure 2

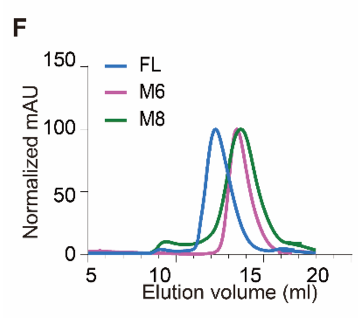

Figure EV1

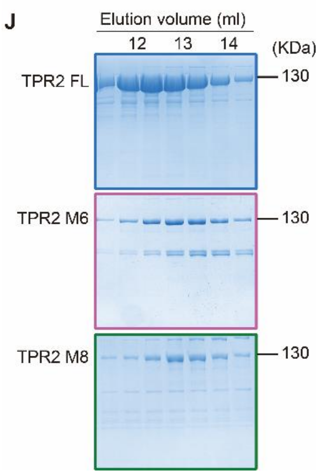

Figure 2. The IDR1 and tetramerization contributes to TPR2 phase separation. (F) Elution profiles of purified TPR2-FL, TPR2-M6, and TPR2-M8 proteins subjected to size-exclusion chromatography.

Figure EV1. **TPR2 phase separation is jointly mediated by N-terminal tetramerization and IDR1.** (J) Fractions with the same elution volume from each injection were subjected to SDS–PAGE.

Minor Points:

• Avoid claiming IDR as a direct condensation drive, which can also act as spacers only or modulators, a nuance the authors' own data supports (IDR2 likely as a spacer).

Response: we thank the reviewer for this important point. We agree that IDRs should not be uniformly described as direct drivers of condensation, as they may also function as spacers or modulators depending on sequence context and molecular environment. In the revised manuscript, we now discuss this issue more explicitly in the context of TPR2 **(line 386-399)**. Our current data support a model in which both the N-terminal

oligomerization domain and IDR1 contribute to TPR2 condensation, whereas IDR2 shows little detectable contribution to LLPS under our assay conditions. As also noted in response to Referee #2, this does not exclude the possibility that IDR2 contributes to other aspects of TPR2 function, even if it is not directly involved in condensation.

• The authors could easily strengthen the bioinformatic support for the roles of IDR1/IDR2 by using established biophysical prediction tools to analyze their potential as "stickers" or "spacers."

Response: we thank the reviewer for this insightful and inspiring suggestion. We agree that the sticker–spacer framework provides a useful way to think about how distinct IDRs may contribute differently to condensation behavior. Although we have not performed a formal residue-level sticker–spacer analysis in the current study, a qualitative inspection of the two sequences suggests that IDR1 and IDR2 are compositionally distinct. In particular, IDR1 is more low-complexity and enriched in Pro/Ala/Gly residues, with sparse hydrophobic/aromatic residues embedded in this background, whereas IDR2 is more compositionally mixed and enriched in charged and polar residues. This is broadly consistent with our functional observations that IDR1 contributes to TPR2 condensation, whereas IDR2 shows little detectable contribution to LLPS under our assay conditions. IDR1 and IDR2 sequences are given below.
At the same time, we agree that it will be very important in future work to characterize these regions at residue-level resolution, including whether IDR1 and IDR2 can be interpreted in terms of distinct sticker and spacer features, and how such sequence grammar contributes to TPR2 assembly and function. We are particularly interested in abiotic stress biology, where changes in molecular interactions and condensate behavior are often closely linked to environmental responses. In this context, a deeper understanding of the residue-level grammar of TPR2 disordered regions may prove especially informative. We thank the reviewer for raising this stimulating point, which suggests an important future direction for this work.
>IDR1 (aa209-285)

NGARAPPPANGPLVGPIPKSAAFPPMGAHAPFQPVVSPSPNAIAGWMTNANPS
LPHAAVAQGPPGLVQPPNTAAFLK
>IDR2 (675-752)
AYEGSRGPPQQINTKPPIVNTLGSVSNVSSPMAVNSERPDRALPTVSMSGLAP
MDVSRTPDVKPRITDESEKVKTWKL

• There are citation errors: ED Fig. 4a, b is incorrectly cited as ED Fig. 3a, b.

Response: we thank the reviewer for catching this error. It has now been corrected in the revised manuscript.

• There are labeling inconsistencies: The ΔC construct is defined as aa 209-1134, but is incorrectly labeled as (ΔC, aa 675-752) in line 157. A thorough check for similar errors is recommended.

Response: we thank the reviewer for pointing this out. The incorrect labeling of the ΔC construct has been corrected in the revised manuscript. We have also carefully re-checked the manuscript for similar labeling inconsistencies and corrected them where necessary.

• Expanding the analysis of TPR2-GFP condensation beyond root tips to other cell types and plant parts would provide valuable physiological insight into its role in development.

Response: we agree that expanding the analysis of TPR2-GFP condensation to additional cell types and tissues would provide further physiological insight. In the present study, we focused on root tip cells because they provide a robust and tractable system for imaging TPR2-GFP condensates *in vivo*. By contrast, imaging GFP signals in other rice tissues, particularly in differentiated tissues such as leaves, is intrinsically more challenging because of stronger autofluorescence from chlorophyll-rich cells,

thicker cell walls, and greater light scattering and optical complexity in mature tissues. We therefore used root tip cells as the most reliable system for the current study. Nevertheless, we agree that a broader survey across additional tissues and developmental stages will be valuable for defining the physiological contexts of TPR2 condensation and represents an important direction for future work.

Referee #2:

- general summary and opinion about the principle significance of the study, its questions and findings

The authors report that the rice corepressor TPR2 undergoes phase separation (likely LLPS) to regulate transcriptional repression. The authors show that TPR2 forms nuclear condensates through the cooperative action of the IDR1 and a plant-specific N-terminal tetramerisation domain. Phase separation-deficient TPR2 mutant variants failed to rescue developmental defects in tpr2 rice mutants, did not establish proper histone acetylation patterns, or effectively repress selected target genes with pleiotropic effects on general (de-)acetylation patterns. Furthermore, the study reveals that TPR2 co-condenses with repressors D53 and IAA3 to facilitate histone deacetylation and gene silencing in a phase separation-dependent manner. This is a significant and timely contribution to understanding transcriptional repression mechanisms in plants, connecting it to the emerging topic of phase separation. The work is relatively well-executed with appropriate approaches. The identification of a condensate-based mechanism for gene silencing fills an important gap in the relevant plant condensate literature. However, several major concerns regarding the specificity of LLPS, the mechanistic connection to histone deacetylation, and the distinction between LLPS-specific vs. oligomerisation-dependent functions need to be addressed. Authors need to improve the overall clarity of assessment, especially with their biochemical analyses and provide the necessary shortcomings of their observations and approaches. The manuscript has substantial merit and makes important contributions, but the concerns raised, particularly regarding mechanism and causality in the in vitro studies, require additional experimental work or more thorough discussion.

Major strengths

1. The authors provide a good demonstration of a condensate-based mechanism for transcriptional repression in plants (paradigm here of TPR2-likely, other TPRs follow the same pattern), filling an important gap in understanding LLPS roles beyond transcriptional activation. I would, though, recommend that the authors consider and succinctly discuss works on transcript expression regulation by other

condensates, such as processing bodies and stress granules.

2. The study combines in vivo and in vitro phase separation assays, structural biology, genetics, genomics, and chromatin analysis to build a narrative for functional condensation.

3. The identification of both IDR1 and the tetramerisation domain as cooperative drivers of phase separation (likely LLPS), with structure-guided mutagenesis (an identified interface from previously published datasets), is relevant.

4. The complementation experiments in rice clearly demonstrate that phase separation capacity correlates with biological function, with graded phenotypes matching those of condensation propensity.

5. The connection between TPR2 condensates, histone deacetylation (H3K9ac), and developmental outcomes provides a relatively meaningful mechanistic insight.

Response: we sincerely thank the reviewer for the positive and thoughtful evaluation of our work.

- specific major concerns essential to be addressed to support the conclusions

1.The authors use 10% PEG8000 as a macromolecular crowding agent to induce phase separation in vitro. While this is a commonly used approach, several concerns arise regarding the physiological relevance and experimental design. First, it remains unclear whether the condensates observed under such relatively high PEG concentrations are representative of assemblies that form in vivo. The use of PEG, particularly of high molecular weight, can artificially "push" phase separation by promoting excluded-volume effects rather than mimicking native crowding. I would agree with the authors if they would comment on the viscosity of the nucleus, where TPR2 condensation takes place, but all additions in the buffer need to be justified. Appropriate controls in the absence of PEG should be included to verify the intrinsic propensity of TPR2 to undergo LLPS. Furthermore, the authors should determine a phase diagram to define the saturation concentration (Csat), as this would provide a quantitative framework to interpret the phase behaviour. The chosen PEG concentration (10%) appears arbitrary;

justification is needed for this specific value, as lower (e.g., 5%) or higher (e.g., 20%) PEG levels could substantially alter condensate formation dynamics. In addition, more information on the physicochemical composition of the in vitro buffer system is required, particularly regarding salt identity and concentration, which can influence phase separation through salting-out or electrostatic screening effects. The inclusion of GFP alone as a negative control is appropriate; however, GFP differs substantially from TPR2 in both size and biophysical properties. Incorporating additional non-phase-separating proteins of comparable size, charge, and domain organisation would strengthen the argument that the observed condensates are specific to TPR2 rather than a general crowding artifact. It would also be important to contextualise the protein concentrations used in vitro (3-10 μM) relative to the estimated physiological levels of TPR2 in rice nuclei. This could be experimentally assessed through nuclear isolation followed by quantitative western blotting with anti-GFP antibodies, normalised to the number of nuclei. Finally, the use of a GFP fusion tag should be explicitly discussed as a potential limitation. GFP can act as a weak solubilising tag and, due to its size and rigidity, may influence condensate nucleation, stability, and molecular dynamics by increasing hydrodynamic radius and reducing diffusion.

Response: we thank the reviewer for this thoughtful and important comment. We agree that PEG8000 does not fully recapitulate the physicochemical complexity of the nucleus and that PEG-induced condensation, by itself, should not be overinterpreted as proof of physiological LLPS. We used PEG8000 as a controlled macromolecular crowding agent to test whether TPR2 has an intrinsic capacity to demix under crowded conditions, while recognizing that the nuclear environment is considerably more complex. We have now added this notion explicitly in the revised manuscript. The nucleus is a highly crowded compartment, and such crowding is thought to favor macromolecular association and compartment formation, but PEG-based assays remain an operational in vitro system rather than a direct mimic of the native nuclear milieu.

To address the reviewer's concern experimentally, we performed a systematic phase-diagram analysis by titrating both TPR2-GFP concentration (1–10 μM) and PEG8000

concentration (1–20%). As shown in **Fig. 1G, H**, TPR2 formed condensates at protein concentrations as low as 2 μM in the presence of only 2% PEG8000, indicating that TPR2 is not dependent on 10% PEG to condense. We selected 10% PEG8000 in the initial assays because it yielded robust and reproducible droplet formation across replicates, which facilitated comparative analyses of wild type and mutant proteins. The newly added phase diagram now provides the quantitative framework requested by the reviewer and shows that 10% PEG8000 is above, rather than defining, the phase boundary.

We also included additional controls addressing intrinsic LLPS propensity and possible tag effects. Under the same buffer conditions, GFP alone remained diffuse, whereas untagged full-length TPR2 formed condensates comparable to those of TPR2-GFP (**Fig. EV1C**), indicating that droplet formation is an intrinsic property of TPR2 rather than an artifact of the GFP fusion. We have also clarified the buffer composition in the Methods section as 20 mM Tris-HCl, pH 8.0, and 150 mM NaCl.

Regarding the physiological relevance of the protein concentrations used in vitro, we agree that direct quantification of endogenous TPR2 concentration in rice nuclei would be valuable. However, this is technically non-trivial in our system because it would require high-purity nuclear isolation from a specific developmental tissue, together with quantitative calibration of endogenous TPR2 abundance at the nuclear level. We therefore refrain from making a direct numerical claim for endogenous TPR2 concentration. Instead, we note more cautiously that the in vitro phase boundary we observe, around 2 μM under modest crowding, lies within the low-micromolar range reported for some nuclear regulatory proteins. Among the relatively few studies that have quantitatively estimated intranuclear concentrations, Belikov et al. reported values of approximately 2.5 μM for GR, 0.25 μM for NF1, and 1.5 μM for Oct1 (Belikov *et al*, 2004), while a later study estimated hormone-induced intranuclear concentrations of steroid receptors at around 1.5 μM (Belikov *et al*, 2016). These values do not establish the nuclear concentration of TPR2, but they do indicate that the concentration regime used in our assays is compatible with reported concentrations for certain nuclear regulatory proteins.

These new data have been incorporated into the revised manuscript (**line 118-131**).

References:

Belikov S, Berg OG, Wrange Ö (2016) Quantification of transcription factor-DNA binding affinity in a living cell. *Nucleic Acids Res* 44: 3045-3058

Belikov S, Holmqvist PH, Astrand C, Wrange O (2004) Nuclear factor 1 and octamer transcription factor 1 binding preset the chromatin structure of the mouse mammary tumor virus promoter for hormone induction. *J Biol Chem* 279: 49857-49867

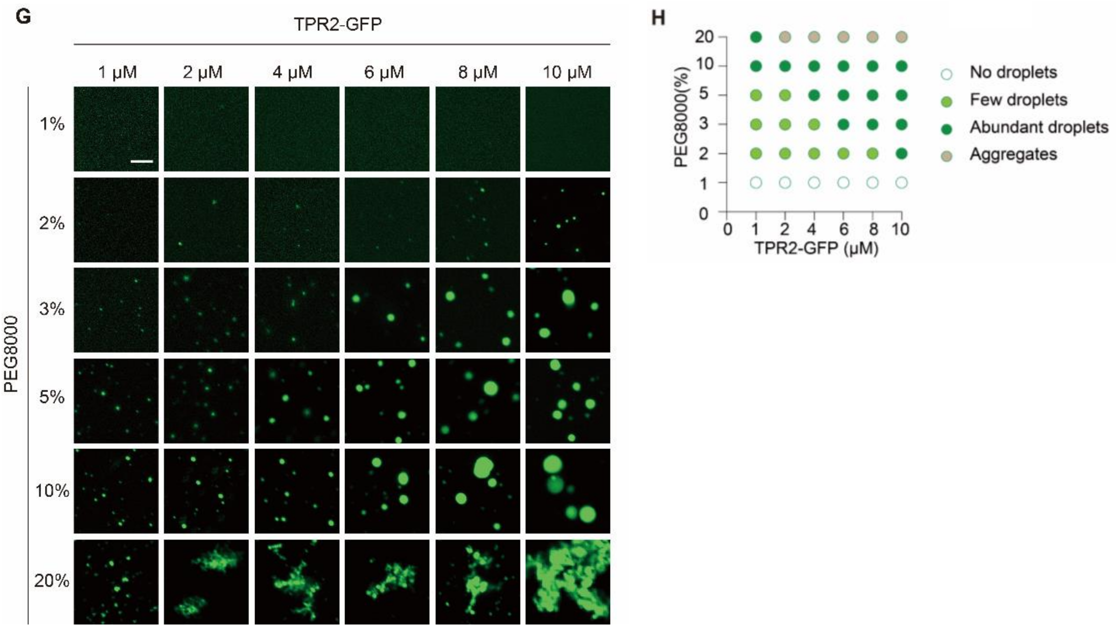

Figure 1. TPR2 forms liquid-like condensates *in vivo* and undergoes phase separation *in vitro*. (**G**) Confocal microscopy images showing TPR2 droplet formation at indicated concentrations of TPR2 and PEG8000. (**H**) *In vitro* TPR2-GFP phase diagram under different PEG8000 concentrations at room temperature. Scale bar = 5 μm.

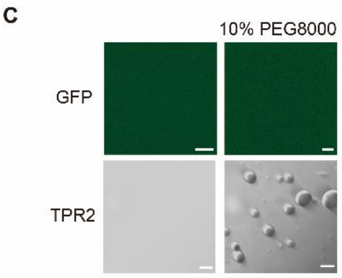

Figure EV1. TPR2 phase separation is jointly mediated by N-terminal tetramerization and IDR1. (**C**) Representative images showing purified GFP and untagged TPR2 proteins incubated with 10% PEG8000. Scale bar = 5 μm.

2.Lack of clarity in oligomerisation biochemistry: for example, lines 168-187, 281-290: The M6 and M8 tetramerisation mutants show reduced or absent condensate formation

in vivo, but in vitro results are more complex: with D53, the M6 and M8 variants form 'solid-like condensates/aggregates' rather than dynamic droplets. D53 in the presence of 10% PEG forms solid-like condensates, which confound the composition/properties of the co-condensate of TRP2/D53 (see, for example, the FRAP data and the linear relationship of signal recovery, suggesting an Ostwald-like ripening process, seen later through the increase in size of the co-condensate). Furthermore, the distinction between phase-separated liquid droplets and aggregates is critical but not always clear. How do the authors distinguish between failed LLPS and aggregation? Are there additional material property measurements (e.g., 1,6-hexanediol sensitivity, fusion dynamics)? Note the very slow dynamics reported in the paper (fusion in the order of minutes, which is not discussed). The gel-like properties of D53 alone (line 281-282) complicate interpretation, and additional discussion would be required. Please, provide additional characterisation and/or discussion of (putative) material properties and clarify terminology distinguishing liquid-phase, gel-phase, and aggregated states.

Response: we thank the reviewer for raising the critical issue of distinguishing between liquid-like, gel-like, and aggregated states in our TPR2-D53 system. We agree that this issue is critical for interpreting the in vitro reconstitution results. In particular, because these assays were performed at relatively high protein concentrations and, where indicated, in the presence of PEG8000 as a macromolecular crowding agent, we recognize that the resulting assemblies may not fully reflect the material properties of condensates formed in vivo. To address it comprehensively, we have characterized the material properties of these assemblies using a combination of FRAP analysis and 1,6-hexanediol (1,6-HD) sensitivity assays, with careful inclusion of appropriate controls.
FRAP measurements revealed a clear difference between wild-type and mutant TPR2-containing assemblies. While TPR2 FL-D53 co-condensates exhibited substantial fluorescence recovery, assemblies formed by TPR2 ΔIDR1 -D53, TPR2 M6 -D53, and TPR2 M8 -D53 were almost completely immobile after photobleaching, with mobile fractions approaching zero (**Fig. EV3E, F**). These results indicate that when TPR2 phase separation is impaired, the mixed assemblies lose dynamic behavior and instead

resemble the more rigid aggregates formed by D53 alone.

We next examined their sensitivity to 1,6-HD, a reagent that selectively disrupts weak hydrophobic interactions characteristic of liquid-like condensates. As expected, TPR2 FL condensates dissolved completely upon 5% 1,6-HD treatment, confirming their liquid-like nature. By contrast, D53 assemblies were fully resistant, consistent with a solid-like or aggregated state in this in vitro assay (**Fig. EV3C**). Importantly, TPR2 FL-D53 condensates were largely dissolved by 1,6-HD, whereas TPR2 ΔIDR1 -D53, TPR2 M6 -D53, and TPR2 M8 -D53 assemblies were unaffected, closely resembling D53 alone (**Fig. EV3G**). These data suggest that, under the in vitro conditions used here, phase separation-defective TPR2 mutants do not support the formation of dynamic co-condensates but are instead incorporated into D53-dominated solid-like assemblies or co-aggregates. Unlike the D53-containing assemblies, all TPR2-IAA3 combinations, including those containing TPR2 mutants, were fully dissolved by 1,6-HD (**Fig. EV3H)**, indicating that the solid-like behavior is not a general feature of TPR2-containing assemblies, but rather reflects a specific property of the TPR2-D53 pair, likely due to the intrinsic tendency of D53 to form less dynamic aggregates in vitro.

Based on these integrated datasets, we have refined the terminology used throughout the manuscript. TPR2 FL alone and TPR2-IAA3 assemblies are now described as liquid-like condensates; D53 alone as a solid-like aggregates in vitro; TPR2 FL -D53 co-condensates as exhibiting liquid-like properties with TPR2 enhancing fluidity; and TPR2 mutant-D53 assemblies as solid-like co-aggregates dominated by D53's intrinsic rigidity.

These new data have been incorporated into the revised manuscript (**line 305-332).**

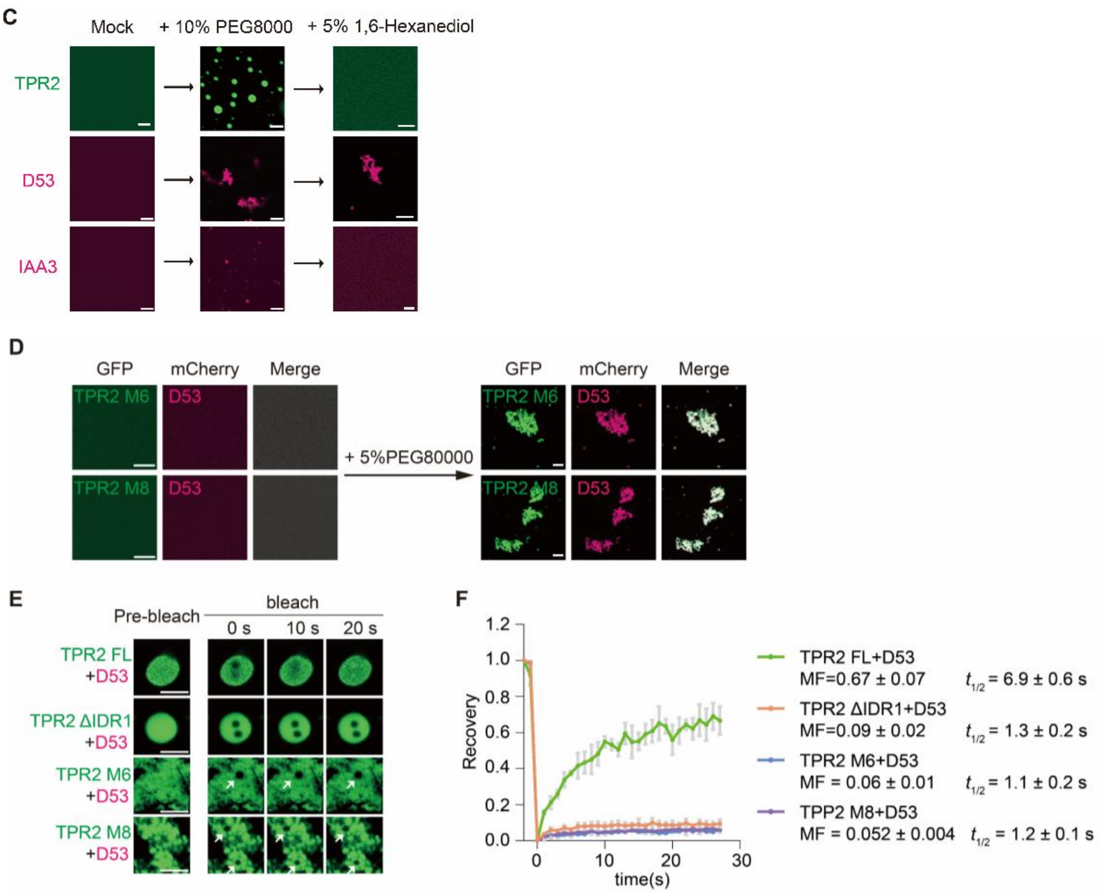

Figure EV3. PEG-induced molecular crowding drives the formation of solid-like D53 assemblies with the phase separation deficient TPR2 mutants. (**C**) Representative confocal images showing purified TPR2-GFP, D53-mCherry, or IAA3-mCherry proteins incubated with 10% PEG8000 followed by addition of 5% 1,6-hexanediol. In the presence of 10% PEG8000 alone, TPR2-GFP formed large and abundant droplets, D53-mCherry formed condensed structures, and IAA3-mCherry formed small and sparse droplets. Upon addition of 5% 1,6-hexanediol, droplets formed by TPR2-GFP and IAA3-mCherry were completely dissolved, whereas D53-mCherry condensates remained visible. Scale bars = 5 μm. (**E**) Representative images of FRAP assays on droplets formed by purified TPR2 FL-GFP and variants(ΔIDR1, M6, M8) with D53-mCherry *in vitro*. (**F**) FRAP recovery curves of TPR2 FL-GFP and variants (ΔIDR1, M6, M8) with D53-mCherry droplets. MF and $t_{1/2}$ values are indicated on the graph. (**G**) Representative confocal images showing purified TPR2-FL-GFP and variants (ΔIDR1, M6, M8) incubated with D53-mCherry in the presence of 10% PEG8000, followed by treatment with 5% 1,6-hexanediol. Under these conditions, droplets formed by TPR2-FL-GFP largely disappeared, while condensates formed by ΔIDR1, M6, and M8 variants remained visible. Scale bars = 5 μm. (**H**) Representative confocal images showing purified TPR2-FL-GFP and variants (ΔIDR1, M6, M8) incubated with IAA3-mCherry in the presence of 10% PEG8000, followed by treatment with 5% 1,6-hexanediol. Under these conditions, phase-separated droplets formed by all TPR2 variant combinations completely disappeared. Scale bars = 5 μm.

3.Lines 271-275: The TPR2 mutants retain physical interactions with D53 and IAA3 (Co-IP and Y2H) but fail to form condensates and cannot rescue function. Is the functional defect truly due to loss of LLPS, or could it result from: altered interaction dynamics/kinetics not captured by Co-IP? Disrupted higher-order assembly

independent of liquid phase properties? Effects on nucleosome binding or other functions? Further discussion is required here.

Response: we thank the reviewer for raising this important conceptual point. We agree that the functional defects observed in the phase separation-defective TPR2 mutants cannot be attributed to loss of LLPS alone with absolute certainty, and that other effects, including altered interaction dynamics not captured by Co-IP or Y2H, disrupted higher-order assembly, or impaired chromatin engagement, may also contribute. Accordingly, we have revised the Discussion to more clearly distinguish binary partner binding from higher-order assembly and to clarify the current limits of causal inference regarding LLPS versus oligomerization-dependent functions.

The following paragraph has been added to the Discussion to explicitly acknowledge (**line 400-415**) this important interpretative consideration, which is broadly relevant to the LLPS field:

"Another important issue raised by our results is how to interpret the loss of function in phase separation-defective TPR2 variants. Although these mutants retain detectable interactions with D53 and IAA3, they show markedly reduced condensation capacity in vivo and fail to restore transcriptional repression, histone deacetylation, or developmental function. These observations indicate that simple binary partner binding is not sufficient for TPR2 activity and instead support the functional importance of condensation-competent higher-order repressive assemblies. At the same time, as in many studies using LLPS-defective variants, it remains difficult to determine to what extent the observed phenotypes reflect disruption of phase-separated organization itself or additional consequences of the mutations, including altered interaction dynamics, changes in assembly architecture, or impaired chromatin engagement that are not captured by Co-IP or yeast two-hybrid assays. More broadly, this highlights an important general consideration in the LLPS field: under physiological conditions, mutations that reduce condensate formation may perturb multiple interconnected properties of macromolecular assemblies, and functional defects therefore cannot always be assigned to loss of LLPS in isolation".

4.Lines 362-375: The discussion of EAR2 motifs promoting TPR2 oligomerisation raises the key question of whether oligomerization per se, rather than liquid phase properties, is the key functional feature; I do not see the suggested causality here. The authors should more explicitly address whether they can distinguish LLPS-specific functions from general requirements for oligomerization or multivalent interactions. This is a fundamental question for the field, but the manuscript creates confusion here. The link between the oligomeric motif and IDR1 interaction is unclear. There is no evidence that oligomer precedes the formation of the condensate. The authors do not attempt any experiment in this direction. I would assume that an opposing scenario would be valid: the oligomeric state is reduced due to the interaction with IDR1 in high Csat (where crowding overtakes low affinity), and hence, the oligomers do not form. Instead, a condensate forms that builds up all the effects described. This intellectual clarity is required as the authors do not address this fundamental query. Furthermore, the authors shall perform SEC analyses or other similar approaches to determine the oligomeric state. This can also be done by purifying the condensates through sedimentation (under conditions promoting condensation). Under the same conditions, the affinity to D53 or IAA3 shall be examined.

Response: we thank the reviewer for raising this important conceptual point. We agree that our current data do not allow us to unambiguously distinguish LLPS-specific functions from the more general requirement for oligomerization or multivalent higher-order assembly, nor do they establish the temporal order between oligomer formation and condensate formation. We also agree that the previous Discussion did not state this point with sufficient clarity.

Our results support three conclusions. First, both the N-terminal tetramerization domain and IDR1 contribute to TPR2 condensation capacity and biological activity. Second, mutations in these regions impair not only condensate formation but also TPR2 function in transcriptional repression, histone deacetylation, and development. Third, partner proteins such as D53 enhance TPR2 condensation, consistent with the idea that

additional multivalent interactions promote higher-order assembly. However, these data do not by themselves demonstrate that LLPS, rather than oligomerization per se, is the sole functional determinant.

We have therefore revised the Discussion to clarify that we favor a model in which oligomerization, multivalency, and LLPS are mechanistically coupled features of the same underlying assembly process. In this framework, the tetramerization domain, IDR1, and partner-derived interaction motifs together promote the formation of condensation-competent repressive compartments. At present, our data do not resolve whether oligomerization precedes condensation, whether condensation further enriches oligomeric species, or whether both emerge cooperatively from the same multivalent interaction network, or whether condensation breaks up oligomeric species. We have also explicitly discussed the broader interpretative issue that, under physiological conditions, mutations that reduce condensate formation may perturb multiple interconnected properties of macromolecular assemblies, and that functional defects therefore cannot always be assigned to loss of LLPS in isolation.

With respect to the reviewer's suggestion for SEC-based analysis, we agree that this is a valuable approach for assessing oligomeric states in dilute solution. Indeed, in response to Referee #1, we have now included SEC data for TPR2-FL, M6, and M8, showing that these mutations alter the oligomeric assembly state of TPR2. At the same time, because SEC is performed under non-condensing conditions, it does not directly report on the assembly state within condensates. For this reason, we have avoided making a strong causal claim that oligomerization precedes LLPS and have instead revised the manuscript to explicitly acknowledge this limitation.

Accordingly, we have revised the Discussion to more clearly distinguish oligomerization, multivalency, and phase separation, and to present our current model as a working framework rather than a resolved mechanistic sequence. This expanded discussion is now incorporated into **paragraphs 2–4** of the revised Discussion.

5.Lines 234-235: 'Immunoblot analyses revealed increased acetylation at histone

H3K9ac, H3K14ac, and H3K27ac in the tpr2 mutant' and lines 71-73: 'TPL/TPRs are known to associate with chromatin-remodelling and histone deacetylase (HDAC) complexes, although whether these interactions are direct or mediated through adaptor proteins remains unclear.' The authors demonstrate that TPR2 condensates are required for histone deacetylation but do not show whether HDACs are enriched in TPR2 condensates, whether the HDAC recruitment/activity is enhanced by LLPS, or the molecular mechanism linking condensate formation to deacetylase activity. They can count a relatively few number of condensates in the nucleoplasm, and the real action may take place in percolation diffraction-limited condensates not visible by confocal microscopy. At least, this interpretation shall be discussed. Perhaps, authors could try to colocalise these complexes in vivo.

Response: we thank the reviewer for raising this important mechanistic question. Indeed, while our genetic data established that TPR2 condensates are required for proper histone deacetylation, the molecular link between phase separation and HDAC activity remained undefined. We have now performed two complementary experiments to address this gap.

To establish a quantitative link between TPR2's phase separation capacity and histone acetylation levels *in planta*, we measured H3K9ac abundance across our panel of transgenic lines by immunoblot. The results, now shown in new **Fig. 4E**, reveal a clear pattern: wild-type ZH11 and the complementation line expressing full-length TPR2 (COM-FL) exhibit low H3K9ac levels, consistent with normal HDAC activity. In contrast, the *tpr2* mutant and complementation lines expressing phase separation-deficient variants (COM-ΔIDR1, COM-M6, COM-M8) all show markedly elevated H3K9ac, comparable to the mutant. This direct correlation between condensate-forming ability and histone deacetylation provides strong *in vivo* evidence that TPR2's phase separation capacity is functionally coupled to chromatin regulation.

Figure 4

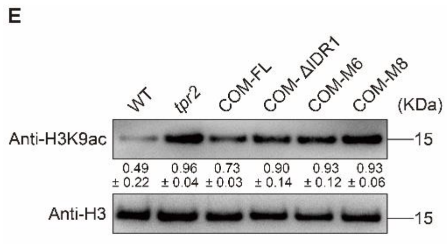

Figure EV2

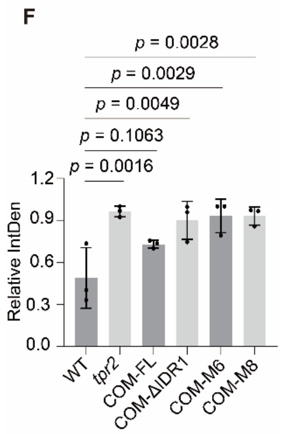

Figure 4. Phase separation of TPR2 negatively regulates H3K9ac levels at its transcriptionally repressed genes. (**E**) Immunoblot analysis of H3K9 acetylation levels in WT, *tpr2*, COM-ΔIDR1, COM-M6 and COM-M8 lines. Histone extracts from WT, *tpr2*, COM-ΔIDR1, COM-M6, and COM-M8 seedlings were subjected to immunoblotting using anti-acetyl-H3K9 antibody. Total H3 was used as a loading control. Experiments were independently repeated at least three times with similar results.

Figure EV2. Phase separation of TPR2 negatively regulates H3K9ac levels at its transcriptionally repressed genes. (**F**) Relative protein levels in **fig. 4E** were quantified by densitometric analysis and normalized to the loading control. Data are mean ± SD (n = 3 biological replicates). P values were determined by one-way ANOVA with Dunnett's multiple-comparisons test.

To explore whether TPR2 condensates may recruit HDACs, we performed co-localization experiments in tobacco by co-expressing TPR2-GFP with two HDACs, HDAC1-BFP and HDT701-BFP, respectively. We observed that HDAC1-BFP, but not HDT701-BFP, partitions into TPR2-GFP condensates (**Response figure 2**). In addition, HDAC1 has previously been reported as a putative interactor of TPR3, a homolog of TPR2, in rice (Tang et al., 2016). These observations are therefore consistent with the possibility that TPR2 condensates may recruit HDACs to modulate histone acetylation. At present, however, because the observed co-localization may reflect indirect rather than direct interactions, we can only propose a tentative model in which a transcription factor-repressor-corepressor-X-HDAC condensate, where X denotes a potential linker protein between the corepressor and the HDAC, contributes to robust chromatin deacetylation. Rigorous testing of this hypothesis will require substantially deeper experimental support, including in vivo visualization of such complexes using multiple fluorescently tagged components under native promoter control, as well as in vitro reconstitution of higher-order condensates containing the relevant components. We believe this represents a highly interesting direction for future work, with potential

conceptual implications not only for the biomolecular condensate field but also for gene regulation and epigenetic control. For these reasons, we decided not to include these preliminary results in the revised manuscript. Instead, we have revised the Discussion to more cautiously consider the potential recruitment of HDACs in our model.

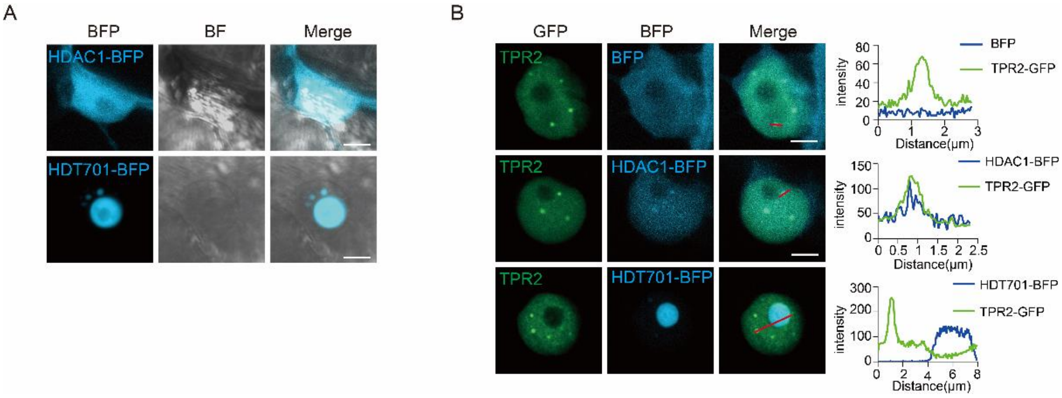

Response figure 2. Subcellular localization and co-localization analysis of HDACs with TPR2. (A) Subcellular localization of histone deacetylases HDAC1 (top) and HDT701 (bottom) in *Nicotiana benthamiana* leaf cells. (**B**) Confocal microscopy analysis of TPR2 co-localization with control BFP (top), HDAC1 (middle), and HDT701 (bottom). TPR2 co-localizes with HDAC1 in nuclear condensates, whereas no detectable co-localization is observed with BFP or HDT701.

6.Lines 361-375: The discussion oligomeriz plant-specific EAR2 motifs in D53, but the relationship between EAR2-mediated oligomerization and LLPS is unclear. Is EAR2 required for co-condensation, or is it merely enhancing? Do other EAR-containing repressors also co-condense with TPR2?

Response: we thank the reviewer for raising this question. To determine whether the EAR2 motif in D53 is required for co-condensation with TPR2, we generated a D53 mutant with a disrupted EAR2 motif (D53 mEAR2) and co-expressed it with TPR2-GFP in tobacco cells.

As shown in **Response figure 3**, D53 mEAR2 retains the ability to form condensates with TPR2, indicating that the EAR2 motif is not strictly required for co-condensation. However, compared to wild-type D53, D53 mEAR2 forms condensates that are significantly smaller in size but increased in number, suggesting that the EAR2 motif contributes to condensate growth rather than serving as an essential trigger for phase separation.

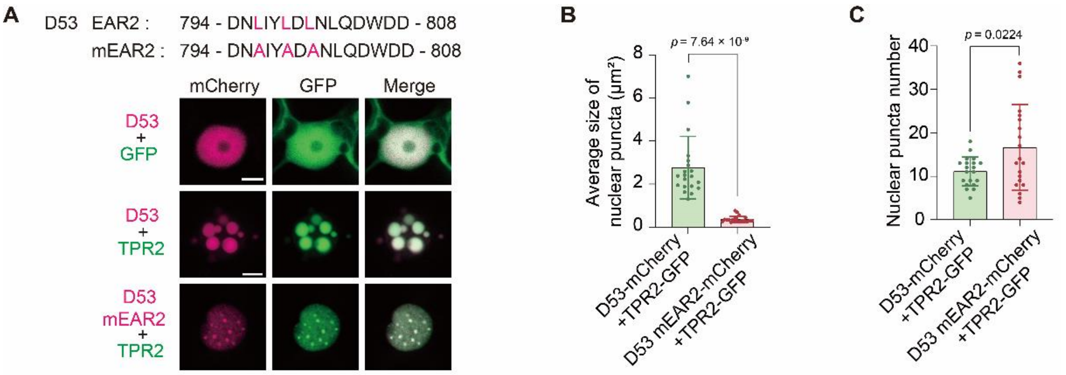

Response figure 3. EAR2 motif mutation weakens D53 co-phase separation with TPR2. (**A**) Confocal images of co-expressing TPR2-GFP with D53-mCherry (top) or D53-mEAR2-mCherry (bottom) in *N. benthamiana* leaf cells. Scale bars = 5 µm. (**B**) Quantification of the average size of nuclear puncta in *N. benthamiana* leaf cells co-expressing TPR2-GFP with D53-mCherry or with D53-mEAR2-mCherry. (n = 20). (**C**) Quantification of the number of nuclear puncta per cell in *N. benthamiana* leaf cells co-expressing TPR2-GFP with D53-mCherry or with D53-mEAR2-mCherry. (n = 20)**.** (**A-C**) Data are mean ± SD Different lowercase letters above bars indicate a significant difference at the $P < 0.05$ level by one-way ANOVA with Tukey's multiple comparisons test.

Regarding other EAR-containing repressors, we tested *DST* (*DROUGHT AND SALT TOLERANCE*), a well-known stress-responsive gene also referred to as *WL1* (*WIDE LEAF 1*), which encodes a TPR2-interacting protein containing the EAR motif. As shown in **Response figure 4**, DST co-localized with TPR2-GFP in nuclear condensates, indicating that recruitment into TPR2 phase-separated may extend to other EAR motif-containing repressors.

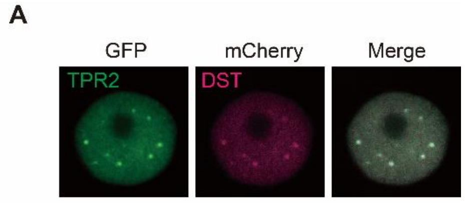

Response figure 4. EAR motif–containing protein DST co-expressed with TPR2 in *N. benthamiana* leaf cells. (**A**) Confocal microscopy analysis showing that DST–mCherry is recruited into TPR2-mediated nuclear condensates.

We were pleased to find that these preliminary observations are consistent with our hypothesis that heterotypic oligomerization may enhance phase separation. However, we decided not to include these data in the current manuscript. As the reviewer correctly pointed out, the mechanistic connection between oligomerization, particularly heterotypic oligomerization, and LLPS is conceptually important and deserves a more

in-depth investigation supported by a more complete dataset before firm conclusions can be drawn. We appreciate the reviewer's interest in this question and consider it an important direction for future work.

7.The authors use only PONDR to predict IDRs. They should extend this part using additional tools for accuracy, and provide the datasets at least as supplemental data. This is because different disorder prediction algorithms use distinct training sets, features, and machine learning approaches, leading to potentially different predictions. Current best practices in the IDR/LLPS field recommend using multiple complementary prediction tools to establish consensus regions. Other tools shall be used to define the molecular grammar of these IDRs, and composition analysis (charge patterning, etc., defined through e.g., PLAAC). Authors could produce a chimeric GFP, testing whether IDR1 and tetramerisation from TPR2 by themselves can drive phase separation in heterologous contexts. Do TPR1 and TPR3 (which also phase separate, Extended Data Fig. 1) have similar IDRs?

Response: we thank the reviewer for the insightful suggestions regarding IDR prediction and validation. We agree that relying on a single prediction tool is insufficient, and that functional validation is essential.

Following the reviewer's recommendation, we have expanded our analysis using the PhaSePred platform, which integrates multiple complementary algorithms including catGRANULE, PLAAC, Pscore, and ESpritz. We submitted the full-length TPR2 sequence for prediction, and the complete results are now provided in **Appendix Fig. S4A**. The analysis reveals that TPR2 exhibits relatively high scores for catGRANULE (0.753, rank 0.727) and Pscore (1.510, rank 0.701), indicating stronger propensity for granule formation and π-π interactions—features commonly associated with phase separation. In contrast, PLAAC and SEG scores are low, suggesting that TPR2 does not contain prion-like domains nor relies on low-complexity sequences for its phase behavior. Regarding TPR1 and TPR3, we examined their sequences using the same prediction pipeline **Appendix Fig. S4B, C**. Both contain predicted IDRs in

regions analogous to TPR2, but sequence alignment reveals no significant similarity among them. This suggests that while the presence of an IDR adjacent to a tetramerization domain may be conserved within the TPR family, the specific sequences have diverged.

A

	catGRANULE	PLAAC	Pscore	ESpritz (DisProt)	Hydropathy	SEG	Charged residue	Phos frequency	DeepPhase	DeepCoil
Score	0.753	-0.010	1.510	0.028	0.466	0.014	0.207	0.000	/	0.000
Rank	0.727	0.910	0.701	0.648	0.414	0.135	0.332	/	/	/

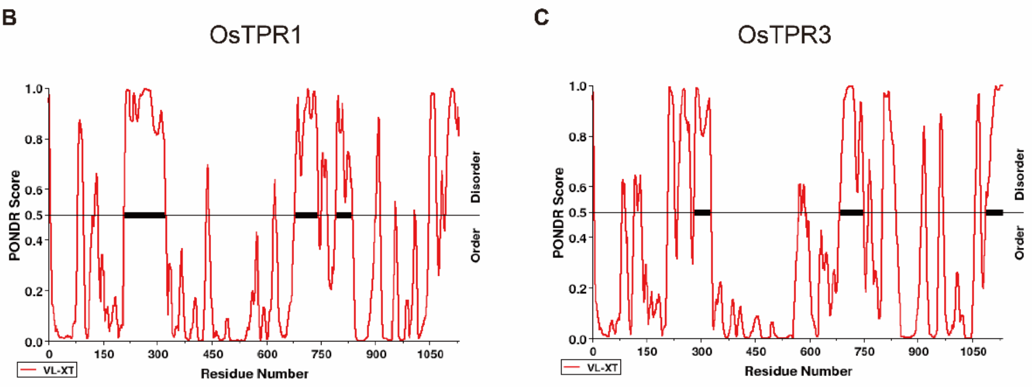

Appendix Figure S4: Prediction of phase separation propensity of rice TPR proteins (**A**) Phase-separation–related sequence properties of OsTPR2 were predicted using the PhaSePred web server (http://predict.phasep.pro/), which integrates multiple predictors including catGRANULE, PLAAC, PScore, ESpritz, DeepPhase and DeepCoil. Scores and proteome-wide ranks for each feature are shown. (**B-C**) Prediction of IDRs in OsTPR1 and OsTPR3 using PONDR (www.pondr.com).

To directly test the phase separation capacity of individual domains, we purified IDR1-GFP and ΔC-GFP proteins. As shown in **Fig. EV1F, G**, ΔC-GFP readily formed phase-separated droplets *in vitro*, whereas IDR1-GFP remained diffuse under identical conditions. This demonstrates that IDR1 alone is insufficient for phase separation, it requires the N-terminal tetramerization domain to achieve the multivalency necessary for condensation. These new data have been incorporated into the revised manuscript (**line 154-157**).

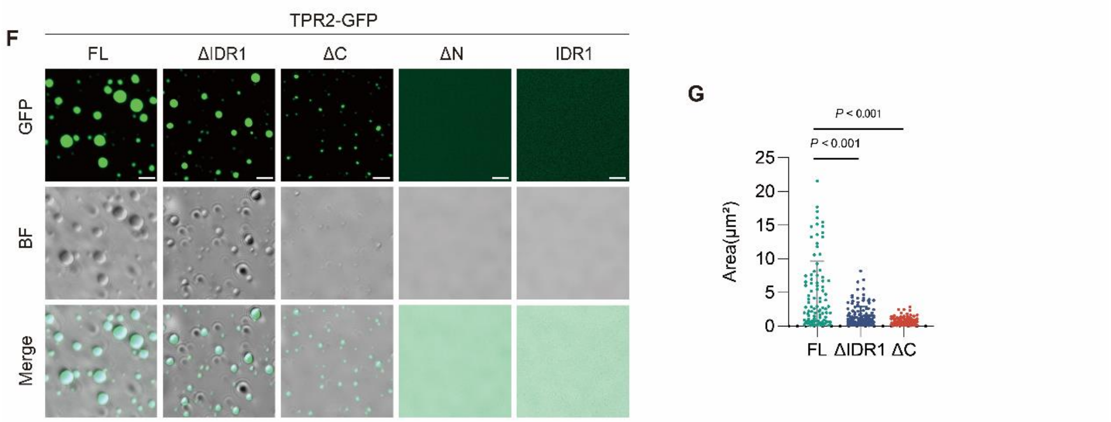

Figure EV1. TPR2 phase separation is jointly mediated by N-terminal tetramerization and IDR1. (**F-G**) *In vitro* phase separation of purified TPR2-FL, ΔIDR1, ΔC, ΔN, and IDR1 proteins in the presence of 10% PEG8000 (**F**). Proteins were labeled with GFP for visualization. Scale bar = 5 µm. (**G**) Quantification of droplet area. Data are mean ± SD (n = 3 fields per group); P values were determined by one-way ANOVA with Dunnett's multiple-comparisons test.

Of lesser concern

1.Lines 145-147: 'Whereas the removal of the IDR2 had little effect on TPR2 LLPS, deletion of the IDR1 domain considerably reduced, but did not eliminate, droplet formation.' If IDR2 has no apparent role in LLPS, what is its function? Is it involved in other aspects of TPR2 activity?

Response: we thank the reviewer for this thoughtful question. Our data indicate that IDR2 is not a major determinant of TPR2 phase separation under the conditions tested. However, this does not exclude an important functional role for this region in other aspects of TPR2 activity. IDRs are common features of transcriptional regulators and can serve functions beyond directly promoting LLPS, including acting as flexible linkers between structured domains, modulating protein conformation, tuning the accessibility or spacing of interaction interfaces, or contributing to context-dependent interactions with other protein or chromatin factors.

At present, we do not have direct experimental evidence defining the specific role of IDR2 in TPR2. One possibility is that IDR2 primarily serves as a structural spacer that helps position adjacent functional regions for optimal assembly or target engagement. Alternatively, it may contribute to regulatory interactions or post-translational

modification-dependent control that were not captured in our current LLPS assays. We agree that this is an interesting question that will require further investigation.

2.Lines 116-118, 277: FRAP recovery is shown for TPR2 alone and co-condensates, but quantitative analysis of recovery rates, mobile fractions, and half-times would be valuable. Comparison between different conditions (FL vs. condensate mutants, {plus minus} repressors) is missing. See also comment 2 in the major concerns above for the description of kinetics.

Response: we thank the reviewer for this helpful suggestion. Accordingly, we have now performed quantitative FRAP analysis and added the mobile fraction (MF) and half-time of recovery ($t_{1/2}$) for all condensate conditions in the revised manuscript. These data are presented in Fig. 1F, K and Fig. 5G, I, M, O.

3.Lines 234-235: Multiple acetylation marks increase in *tpr2* mutants (H3K9ac, H3K14ac, H3K27ac), but CUT&Tag focuses only on H3K9ac. Are other repressive marks, such as H3K27me3, H3K9me2, affected? This would provide a more complete picture of chromatin state changes-the authors provide only correlative evidence.

Response: we thank the reviewer for this important suggestion. In our initial immunoblot analyses, we found that multiple acetylation marks, including H3K9ac, H3K14ac, and H3K27ac, were elevated in the *tpr2*. Among these, H3K9ac showed the most robust and reproducible difference, and we therefore selected it as the primary readout for the subsequent CUT&Tag analyses. This choice was intended to provide a sensitive and reliable assay for assessing chromatin changes associated with TPR2 function. To further address the reviewer's question regarding other repressive chromatin marks, we examined H3K9me2 levels in ZH11 and *tpr2* by immunoblot and did not detect a significant difference between the two genotypes (**Response figure 5**). This result is consistent with the current view that TPR2 primarily acts through histone deacetylation-associated repression, rather than through broad changes in H3K9

demethylation.

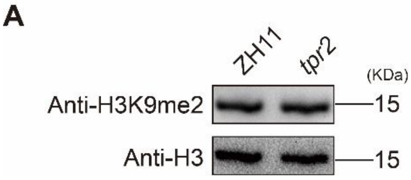

Response figure 5. H3K9me2 levels in ZH11 and *tpr2* by immunoblot. (A) Immunoblot analysis of H3K9me2 levels in WT and *tpr2* seedlings. Total H3 was used as a loading control. Experiments were independently repeated at least three times with similar results.

4.The manuscript does not address: How quickly condensates form/dissolve, and whether condensate dynamics correlate with gene expression kinetics and how simple developmental or hormonal signals might regulate TPR2 condensation.

Response: we thank the reviewer for raising this important point. We agree that the kinetics of TPR2 condensate formation and dissolution, as well as their relationship to gene expression dynamics and developmental or hormonal regulation, are highly relevant questions. In the current study, our primary focus was to establish that TPR2 forms condensates, to define the structural determinants underlying this behavior, and to connect condensation capacity with transcriptional repression and biological function. We did not systematically examine the real-time kinetics of condensate assembly/disassembly or their temporal coupling to transcriptional responses.

Our current FRAP analyses provide evidence that TPR2 condensates are dynamic, but they do not directly address how rapidly condensates form or dissolve in response to developmental or signaling cues. Likewise, although TPR2 is known to act in hormone-responsive pathways through partners such as D53 and IAA3, the extent to which hormonal or developmental signals acutely regulate TPR2 condensation remains unresolved. We agree that these are important directions for future work, and that live-cell imaging combined with inducible hormonal treatments and time-resolved gene expression analyses will be required to address these questions directly.

5.Extended Data Figure 5a: The schematic of mutation sites would benefit from

showing the actual structural context.

Response: we thank the reviewer for this suggestion. The mutation sites are shown in their structural context in current version **Fig. 2E, and the positional information of these residues are shown in** current version **Figure EV1H.**

6.Figure 6a, b: IGV browser tracks need scale bars and y-axis labels for direct comparison.

Response: we thank the reviewer for this suggestion. We have now added complete scale bars and y-axis labels to Figure 6a and 6b to enable direct quantitative comparison across samples.

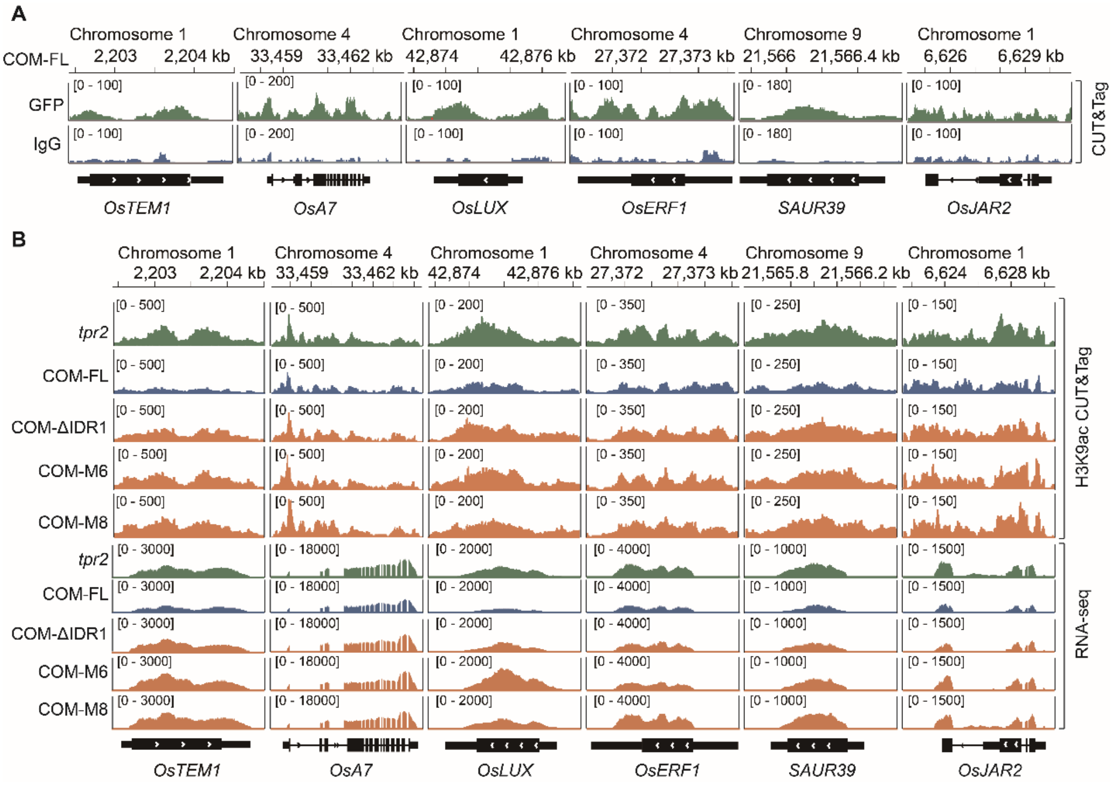

Figure 6. Phase separation of TPR2 promotes transcription suppression with repressors. (**A**) Integrative genome browser tracks showing GFP CUT&Tag signals at the same loci in COM-FL plants. (**B**) Integrative genome browser visualization of H3K9ac CUT&Tag signals (top) and RNA-seq signals (bottom) at *OsTME1*, *OsA7*, *OsLUX*, *OsERF1*, *SAUR39*, and *OsJAR2* in *tpr2* and *pTPR2::TPR2FL-GFP/tpr2* (COM-FL), *pTPR2::TPR2ΔIDR1-GFP/tpr2* (COM-ΔIDR), *pTPR2::TPR2M6-GFP/tpr2* (COM-M6), and *pTPR2::TPR2M8-GFP/tpr2* (COM-M8) plants. (**A-B**) Data from 20-day-old rice seedlings.

7.Lines 428-430: 'Phase separation was induced by supplementing the reaction with 10% (w/v) PEG8000'. In addition, temperature control details?

Response: we thank the reviewer for this query. All *in vitro* phase separation assays were performed at room temperature (~22°C).

8.Abstract: acronyms shall be explained, for example, D53, IAA3 and TPR. Furthermore, the presumptive dynamicity has not been shown. Do they dissolve in the presence of hexanediol or other compounds?

Response: we thank the reviewer for these helpful suggestions. We have revised the Abstract to define all acronyms upon first use, including D53 (DWARF 53), IAA3 (AUXIN/INDOLE-3-ACETIC ACID INDUCIBLE 3), and TPR (TOPLESS-RELATED).

Regarding the request for evidence of condensate dynamicity, we treated TPR2, D53, and IAA3 assemblies with 5% 1,6-hexanediol (**Fig. EV3C**). TPR2 condensates dissolved completely, consistent with liquid-like properties. IAA3 assemblies, which formed weak phase separation, also dissolved upon treatment. In contrast, D53 aggregates remained unaffected, indicating their gel-like or solid-like nature. These results have been incorporated into the revised manuscript (**line 305-309**).

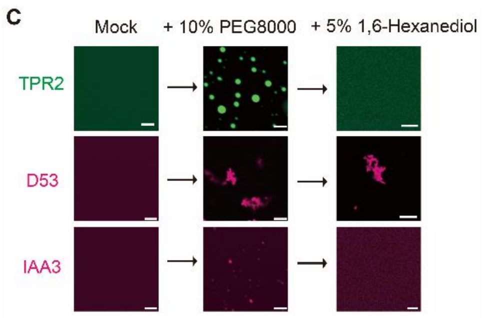

Figure EV3. PEG-induced molecular crowding drives the formation of solid-like D53 assemblies with the phase separation deficient TPR2 mutants. (C) Representative confocal images showing purified TPR2-GFP, D53-mCherry, or IAA3-mCherry proteins incubated with 10% PEG8000 followed by addition of 5% 1,6-hexanediol. In the presence of 10% PEG8000 alone, TPR2-GFP formed large and abundant droplets, D53-mCherry formed condensed structures, and IAA3-mCherry formed small and sparse droplets. Upon addition of 5% 1,6-hexanediol, droplets formed by TPR2-GFP and IAA3-mCherry were completely dissolved, whereas D53-mCherry condensates

remained visible. Scale bars = 5 μm.

9.Line 106: The cleavage of the TPR2 by NAL1 is not fully explained here. Does it also play a role in the condensation process?

Response: we thank the reviewer for raising this point. Our previous study identified TPR2 as a substrate of the protease NAL1. To explore whether NAL1-mediated cleavage affects TPR2 condensation, we co-expressed TPR2-GFP with NAL1-mCherry in tobacco cells. The results were heterogeneous: in some cells, TPR2 condensates were completely dissolved; in others, several nuclear puncta remained; and in a subset of cells, large nuclear aggregates were observed. This complexity suggests that NAL1 may modulate TPR2 condensation in a context-dependent manner, possibly through partial cleavage or spatiotemporal regulation (**Response Figure 6**).

Because these results are complex and preliminary, and because the relationship between NAL1-mediated cleavage and TPR2 condensation is not a central focus of the present study, we have not incorporated these data into the main manuscript. Nevertheless, we agree that this is an intriguing question that warrants further mechanistic investigation in future work.

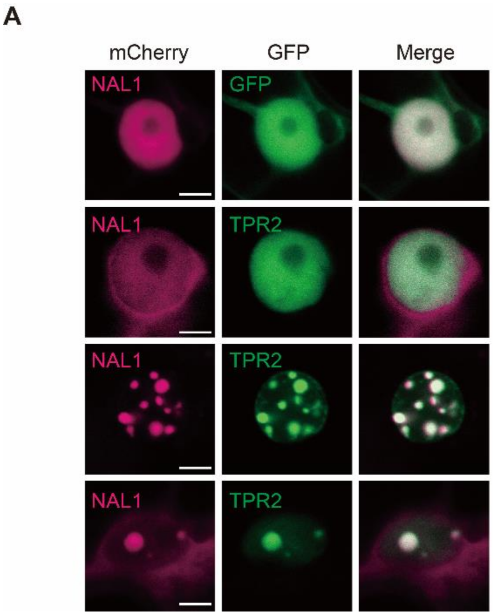

Response figure 6. Subcellular localization of NAL1 co-expressed with TPR2. (A) Representative subcellular localization patterns of NAL1 when co-expressed with GFP or TPR2 (showing three distinct localization types) in *N. benthamiana* leaf cells.

10.Line 128: As suggested above, the mobile fraction shall be defined.

Response: we thank the reviewer for this suggestion. As recommended, we have now included mobile fraction (MF) and half-time of recovery ($t_{1/2}$) for all FRAP data throughout the manuscript

11.Line 142 and elsewhere: the IDR acronym has been defined, and the authors still spell it out.

Response: we thank the reviewer for this careful observation. We have corrected these errors throughout the manuscript.

12.Line 281: Please, rephrase as using the word 'under' is a bit confusing and may mean "lower than".

Response: we thank the reviewer for this wording suggestion, we have rephrased the sentence to: "In the presence of 10% PEG8000, D53 formed gel-like aggregates."

13.The GFP-CUT&Tag shall be explained for non-experts.

Response: we thank the reviewer for this suggestion. We have added a brief explanation of the GFP-CUT&Tag technique in the Results section to make it accessible to non-specialists. The revised text now reads:

To determine whether TPR2 is associated with these loci in vivo, we performed GFP-CUT&Tag in *pTPR2::TPR2-GFP/tpr2* plants to selectively enrich genomic loci bound by TPR2-GFP. In this approach, an anti-GFP antibody is used to selectively target the GFP-tagged TPR2 protein and thereby enrich nearby chromatin fragments, allowing high-resolution mapping of genomic regions associated with TPR2. Compared with IgG-CUT&Tag controls, significant enrichment was observed at these loci (Fig. 6A and

Appendix Fig. S3A), indicating that TPR2 is associated with these potential target genes in planta. (**line 349-354**).

14.In general, the proteins purified are of low purity (for example, Extended Data Fig. 5 and 11, for D53 and IAA3). Could the authors comment on this? I agree that sometimes, especially with IDR-containing proteins, high purity cannot be achieved. Did the authors try to tandemly use additional purification steps? This is a certain limitation in the approach they used, and shall be highlighted in the text.

Response: we thank the reviewer for this constructive comment. We agree that protein purity is critical for *in vitro* phase separation assays.

Following this suggestion, we have re-purified D53-mCherry and IAA3-mCherry using an additional size exclusion chromatography (SEC) step after affinity purification. This yielded substantially purer protein preparations, as shown in the updated **Fig. EV3B**. All *in vitro* experiments involving D53 and IAA3 have been repeated using these purified proteins, and the results remain consistent with our previous conclusions. We have updated the Methods section accordingly to reflect the revised purification protocol (**line 483-488**).

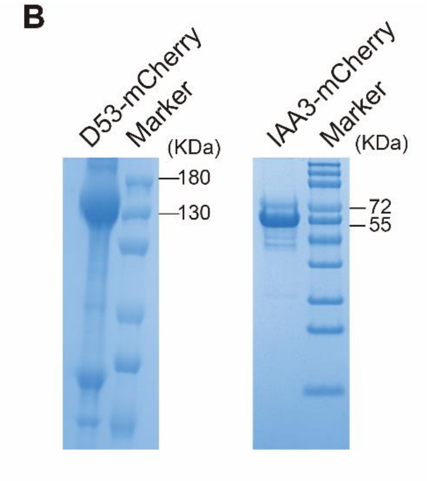

Figure EV3. PEG-induced molecular crowding drives the formation of solid-like D53 assemblies with the phase separation deficient TPR2 mutants. (**B**) SDS-PAGE analysis of D53-mCherry and IAA3-mCherry proteins after affinity purification followed by size-exclusion chromatography.

15.Extended Fig.7: The b and c panels are interesting, but are not sufficiently discussed in the text; I also miss the context here.

Response: we thank the reviewer for pointing this out. We agree that the biological context of Extended Fig. 7b, c was not sufficiently explained in the original manuscript. We have now expanded the corresponding text to better interpret these enrichment results. In particular, we note that the enrichment analysis recovered pathways closely related to known TPR2-associated functions. Plant hormone signal transduction emerged as the most significantly enriched term in the KEGG pathway analysis, and defense response was also prominently enriched in the GO analysis, consistent with the established roles of TPR family proteins in the transcriptional regulation of growth, stress responses, and immunity. Beyond these expected categories, we also observed enrichment of secondary metabolism-related terms, suggesting that TPR2 may indirectly influence defense-associated metabolic programs. In addition, the enrichment of nuclear export-related terms raises the possibility that TPR2 condensates may coordinate not only transcriptional repression but also aspects of RNA or protein export, a hypothesis that warrants further investigation. These points are now discussed in the revised manuscript (**line 237-249**).

16.Some additional clarity on statistics would be required in the Figure Legends. For example, authors report a population size of n=6 for seed setting, but it is not fully clear from how many biological replicates the population size comes from.

Response: we thank the reviewer for this suggestion. We have revised all Figure legends to provide clearer statistical information. For seed setting data, the reported population size ($n = 6$) represents 6 biological replicates, each biological replicate representing the average seed setting rate of three panicles per plant. This clarification has been added to the Figure legend, and similar details have been included for all other quantitative data in the revised manuscript.

17.Fig. 3a: In the M8, panel a, the image quality is either low or the protein has diffused into the nucleolus. Could the authors please comment? Why do they pick regions with different distances from each nucleus for this panel? The same cells shall be used.

Response: we thank the reviewer for pointing this out. Upon re-examination, we confirmed that the M8 signal in the original image appeared ambiguous due to suboptimal focusing. We have now replaced it with a clearer image from the same experiment showing that M8-GFP does not localize to the nucleolus but remains diffuse in the nucleoplasm. All images in **Fig. 3A** are now from representative cells with comparable nuclear regions. The Figure has been updated accordingly.

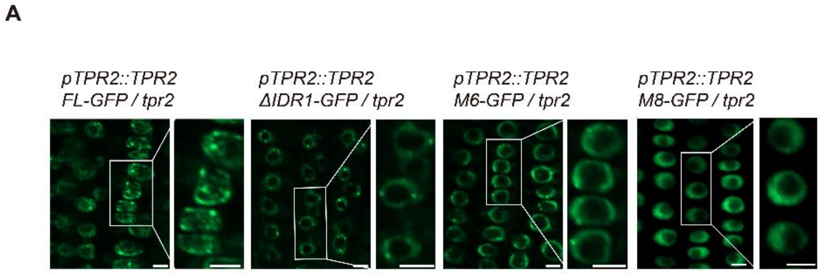

Figure 3. Phase separation is essential for TPR2 to regulate rice growth and development. (A) Confocal images of lateral root tip cells of *pTPR2::TPR2FL-GFP/tpr2* (COM-FL), *pTPR2::TPR2ΔIDR1-GFP/tpr2* (COM-ΔIDR), *pTPR2::TPR2M6-GFP/tpr2* (COM-M6) and *pTPR2::TPR2M8-GFP/tpr2* (COM-M8). Scale bar = 5μm. Representative images from three independent experiments.

Dear Prof. Lai,

Thank you for submitting a revised version of your manuscript. The study has now been evaluated by all original referees. Referee #1 considers that their previous concerns have been satisfactorily addressed and now recommends publication. Referee #2 is also overall supportive but raises several minor points, which we find balanced and constructive and would ask you to address in a revised version of the manuscript.
In addition, there remain a few mainly editorial points that will need to be addressed before I can proceed with formal acceptance of the manuscript.

- FUNDING INFO: missing in eJP: the Natural Science Foundation of Hubei Province (2023AFA095), the Fundamental Research Funds for the Central Universities (2662025SKPY007), the Ministry of Science of Technology of China (Cai Yuanpei - Project), and the Earmarked Fund for China Agriculture Research System (CARS-01)

- As we are switching from a free-text author contribution statement towards a more formal statement based on Contributor Role Taxonomy (CRediT) terms, please remove the present Author Contribution section and instead specify each author's contribution(s) directly in the Author Information page of our submission system during upload of the final manuscript. See https://casrai.org/credit/ for more information.

- FIGURE CALLOUTS: missing callout for Appendix Figure S4

- DATASET EV LEGENDS: source file names, titles, legends and manuscript callouts all need to be updated to Dataset EV1-EV4 instead of EV Tables; they should be uploaded individually as Dataset files with legends in a separate tab/sheet in each Excel file
APPENDIX 1 FILE WITH ToC: author info (names and affiliations) could be removed from title page; highlights should be removed from text, as Appendix PDF will not be typeset

- Please provide suggestions for a short 'blurb' text prefacing and summing up the conceptual aspect of the study in two sentences (max. 250 characters), followed by 3-5 one-sentence 'bullet points' with brief factual statements of key results of the paper; they will form the basis of an editor-written 'Synopsis' accompanying the online version of the article. Please also provide an altered synopsis image, making sure that the aspect ratio conforms to our website's format - it should be exactly 550 pixels wide and 300 pixels high.

- Figure Legends (main + EV):
1. Please note that the exact p values are not provided in the legends of figures 2D, H, J; 5B-E; EV1 G

2. Please indicate the statistical test used for data analysis in the legends of figures 4A, EV2 D, E

3. Please note that information related to n is missing in the legend of figure 4A

4. Please note that the scale bar needs to be defined for figures 1G, EV3 D, E

5. Please note that the white arrows are not defined in the legend of figure 1I, 5F, H; 5L, N; EV3 E. This needs to be rectified.

- Sections need to be named and the order should be corrected: Title page - Abstract - Introduction - Results - Discussion - Methods - Data Availability - Acknowledgements - Disclosure and Competing Interests Statement - References - Figure Legends - Table(s) - Expanded View Figure Legends.

Please let me know if you have any questions regarding any of these points. You can use the link below to upload the revised files.
Thank you again for giving us the chance to consider your manuscript for The EMBO Journal. I look forward to receiving the final version.

With best regards,

Cornelius Schneider

Cornelius Schneider, PhD
Editor | The EMBO Journal
c.schneider@embojournal.org

Please refer to our figure preparation guideline in order to ensure proper formatting and readability in print as well as on screen:

https://link.springer.com/journal/44318/submission-guidelines#cms-Figure-and-data-presentation

Use the link below to submit your revision:

https://emboj.msubmit.net/cgi-bin/main.plex

Referee #1:

The revised manuscript has successfully addressed the major concerns raised through substantial new experimentation and improved data presentation. The scientific framework linking TPR2 phase separation to chromatin-based gene repression is now better supported by biochemistry, genetic, and genomic evidence at high quality. I am satisfied with the revision and supportive of the publication by the EMBO Journal.

Referee #2:

Authors considered most of the comments; there are some that require some more work-either textually or experimentally:
The phase diagram (Fig. 1G,H) showing condensation at 2 µM 2% PEG is a strong addition; it directly answers the concern that 10% PEG was arbitrary and shows TPR2 condenses well below that threshold. The untagged TPR2 control (Fig. EV1C) adequately addresses the GFP tag concern. Buffer composition is now stated. The citation of Belikov et al. for nuclear protein concentrations in the low-micromolar range is reasonable but indirect, as they openly acknowledge they cannot quantify endogenous TPR2, which is honest.
The combination of FRAP (Fig. EV3E,F) with mobile fractions near zero for mutant-D53 assemblies, plus 1,6-hexanediol sensitivity assays distinguishing TPR2-FL (dissolved) from D53 alone (resistant) and mutant-D53 co-aggregates (resistant), directly addresses the concern. The refined terminology (liquid-like, solid-like, co-aggregate) is appropriate. The comparison between D53- and IAA3-containing assemblies (IAA3 combinations all dissolve) is a useful internal control showing the solid-like behavior is D53-specific.
One minor issue that I sitll have: I flagged the slow fusion dynamics (minutes timescale) and asked for explicit discussion. The response doesn't directly address fusion kinetic; it focuses on FRAP. This is a soft gap but not critical.
The SEC data (Fig. 2F) showing M6 and M8 as dimers vs FL as tetramer is a valuable addition. The revised Discussion acknowledging that the temporal order (oligomer-first vs condensate-first) is unresolved is honest and appropriate. I specifically suggested purifying condensates by sedimentation and measuring affinity to D53/IAA3 under condensing conditions. This was not done, nor was it explicitly acknowledged as a limitation. The response pivots entirely to SEC under dilute (non-condensing) conditions and notes that SEC doesn't report on assembly within condensates, which is correct but does not substitute for the suggested experiment. A sentence acknowledging this as a future direction would have strengthened the manuscript.
The HDAC1-BFP co-localization with TPR2 condensates (but not HDT701-BFP) is a clean result. The H3K9ac immunoblot across all complementation lines (Fig. 4E) showing elevated acetylation in all LLPS-deficient lines is convincing correlative evidence. The decision not to include the HDAC co-localization in the main manuscript, because it is preliminary and the interaction may be indirect, is conservative and defensible. The revised discussion appropriately proposes a TF-repressor-corepressor-X-HDAC model. I also raised the possibility that sub-diffraction-limit percolation condensates may be doing the work rather than the visible puncta. This point is not addressed in the response. It is a conceptually important point that deserved at least a sentence...
M6 and M8 both elute as apparent dimers, but these mutants target different tetramerization interfaces. The authors correctly note that SEC alone cannot determine which interface is retained. However, the revised model now represents both as "dimers", which is parsimonious but loses the original rationale for designing two different mutants (M6 vs M8 were supposed to distinguish the two interfaces). If both look the same by SEC and both behave similarly in all functional assays, the reader is left wondering what the two-mutant strategy actually resolved. This point deserves explicit discussion.
The authors use 1,6-HD sensitivity as a key criterion for distinguishing liquid-like from solid-like assemblies. However, 1,6-HD disrupts weak hydrophobic interactions non-specifically and can dissolve assemblies that are not phase-separated (e.g., certain protein complexes held together by hydrophobic interfaces). Recent literature (Wheeler et al., Kroschwald et al.) has emphasized that 1,6-HD sensitivity alone is insufficient to demonstrate LLPS. The authors use it as confirmatory alongside FRAP, which is better than relying on it alone, but they never discuss the limitations of 1,6-HD as a tool.
All minor issues have been resolved.

Referee #1:

The revised manuscript has successfully addressed the major concerns raised through substantial new experimentation and improved data presentation. The scientific framework linking TPR2 phase separation to chromatin-based gene repression is now better supported by biochemistry, genetic, and genomic evidence at high quality. I am satisfied with the revision and supportive of the publication by the EMBO Journal.

Response: We sincerely thank the reviewer for the thoughtful and constructive comments, which have significantly improved our work.

Referee #2:

Authors considered most of the comments; there are some that require some more work-either textually or experimentally:

The phase diagram (Fig. 1G,H) showing condensation at 2 μM 2% PEG is a strong addition; it directly answers the concern that 10% PEG was arbitrary and shows TPR2 condenses well below that threshold. The untagged TPR2 control (Fig. EV1C) adequately addresses the GFP tag concern. Buffer composition is now stated. The citation of Belikov et al. for nuclear protein concentrations in the low-micromolar range is reasonable but indirect, as they openly acknowledge they cannot quantify endogenous TPR2, which is honest.

Response: We thank the reviewer for acknowledging the improvements made in our revised manuscript.

The combination of FRAP (Fig. EV3E,F) with mobile fractions near zero for mutant-D53 assemblies, plus 1,6-hexanediol sensitivity assays distinguishing TPR2-FL (dissolved) from D53 alone (resistant) and mutant-D53 co-aggregates (resistant), directly addresses the concern. The refined terminology (liquid-like, solid-like, co-aggregate) is appropriate. The comparison between D53- and IAA3-containing assemblies (IAA3 combinations all dissolve) is a useful internal control showing the solid-like behavior is D53-specific.

Response: We thank the reviewer for the positive assessment of our revision.

One minor issue that I sitll have: I flagged the slow fusion dynamics (minutes timescale) and asked for explicit discussion. The response doesn't directly address fusion kinetic; it focuses on FRAP. This is a soft gap but not critical.

Response: We thank the reviewer for this helpful comment. We agree that the *in vitro* fusion of TPR2 droplets is relatively slow. However, the *in vivo* TPR2 condensates showed much faster FRAP recovery, within approximately 20s, indicating dynamic molecular exchange in plant nuclei. The difference between *in vitro* and *in vivo* kinetics may reflect the distinct assay conditions, as purified TPR2 droplets formed under PEG-induced crowding may have different size, concentration, and material properties from condensates formed in the cellular nuclear environment. Therefore, we consider the *in vitro* fusion behavior compatible with the overall conclusion that TPR2 forms dynamic, liquid-like condensates.

The SEC data (Fig. 2F) showing M6 and M8 as dimers vs FL as tetramer is a valuable addition. The revised Discussion acknowledging that the temporal order (oligomer-first vs condensate-first) is unresolved is honest and appropriate. I specifically suggested purifying condensates by sedimentation and measuring affinity to D53/IAA3 under condensing conditions. This was not done, nor was it explicitly acknowledged as a limitation. The response pivots entirely to SEC under dilute (non-condensing) conditions and notes that SEC doesn't report on assembly within condensates, which is correct but does not substitute for the suggested experiment. A sentence acknowledging this as a future direction would have strengthened the manuscript.

Response: We thank the reviewer for this helpful suggestion. We agree that biochemical analysis of the condensed phase would be informative. However, how heterotypic condensates form, how their components are organized and interact within the condensed phase, and how such interactions can be quantitatively described remain challenging questions in the condensate field. We have added a sentence to acknowledge this point as an important future direction in line 452-455.

The HDAC1-BFP co-localization with TPR2 condensates (but not HDT701-BFP) is a clean result. The H3K9ac immunoblot across all complementation lines (Fig. 4E) showing elevated acetylation in all LLPS-deficient lines is convincing correlative evidence. The decision not to include the HDAC co-localization in the main manuscript, because it is preliminary and the interaction may be indirect, is conservative and defensible. The revised discussion appropriately proposes a TF-repressor-corepressor-X-HDAC model. I also raised the possibility that sub-diffraction-limit

percolation condensates may be doing the work rather than the visible puncta. This point is not addressed in the response. It is a conceptually important point that deserved at least a sentence...

Response: We thank the reviewer for this thoughtful comment. We agree that the visible TPR2 puncta observed by confocal microscopy may represent only one form of higher-order TPR2 assembly. Smaller sub-diffraction-limit or percolated assemblies could also contribute to local HDAC recruitment, chromatin deacetylation, and transcriptional repression. We have added a sentence to the Discussion to acknowledge this possibility in line 476-480.

M6 and M8 both elute as apparent dimers, but these mutants target different tetramerization interfaces. The authors correctly note that SEC alone cannot determine which interface is retained. However, the revised model now represents both as "dimers", which is parsimonious but loses the original rationale for designing two different mutants (M6 vs M8 were supposed to distinguish the two interfaces). If both look the same by SEC and both behave similarly in all functional assays, the reader is left wondering what the two-mutant strategy actually resolved. This point deserves explicit discussion.

Response: We thank the reviewer for this insightful comment. We agree that the rationale and limitation of the M6/M8 strategy should be discussed more explicitly. M8 contains two additional substitutions on the basis of M6 and was initially designed to further disrupt TPR2 tetramerization. However, SEC showed that both M6 and M8 elute as apparent dimers, indicating that these mutations compromise tetramerization but do not fully disrupt dimer formation. Therefore, these mutants cannot distinguish the specific function of each dimerization interface, nor can SEC determine which interface is retained. We have added several sentences (Line396-410) to clarify this limitation and to note that future generation of *bona fide* monomeric TPR2 variants will be important to further define how structured oligomerization contributes to TPR2 condensation and function.

The authors use 1,6-HD sensitivity as a key criterion for distinguishing liquid-like from solid-like assemblies. However, 1,6-HD disrupts weak hydrophobic interactions non-specifically and can dissolve assemblies that are not phase-separated (e.g., certain protein complexes held together by hydrophobic interfaces). Recent literature (Wheeler et al., Kroschwald et al.) has emphasized that 1,6-HD sensitivity alone is insufficient to demonstrate LLPS. The authors use it as confirmatory

alongside FRAP, which is better than relying on it alone, but they never discuss the limitations of 1,6-HD as a tool.

Response: We thank the reviewer for raising this important point. We agree that 1,6-hexanediol sensitivity is not specific to LLPS and should not be used as a stand-alone criterion for defining phase-separated assemblies. In our study, we used 1,6-hexanediol only as a complementary assay to compare the material properties of different TPR2-repressor assemblies, together with FRAP, droplet formation, fusion behavior, concentration-dependent condensation, variant analyses, and in vivo functional assays. We have now added text to explicitly discuss both the usefulness and limitations of 1,6-hexanediol (Line 426-432). In light of the reviewer's comment, we have also expanded the Discussion to note that chemical perturbation is more feasible at the cellular level than at the whole-plant level, and that orthogonal engineering approaches, such as fusion to MBP or oligomerization domains to reduce or enhance condensation, respectively, may help further dissect the functional contribution of LLPS in future studies (Line 432-438).

All minor issues have been resolved.

Response: We sincerely thank the reviewer for the thoughtful and constructive comments, which have significantly improved our work.

Dear Prof. Iai,

I am pleased to inform you that your manuscript has been accepted for publication in the EMBO Journal.

Your manuscript will be processed for publication by EMBO Press. It will be copy edited and you will receive page proofs prior to publication. Please note that you will be contacted by Springer Nature Author Services to complete licensing and payment information.

You may qualify for financial assistance for your publication charges - either via a Springer Nature fully open access agreement or an EMBO initiative. Check your eligibility: https://link.springer.com/journal/44318/how-to-publish-with-us

Should you be planning a Press Release on your article, please notify the production team via this form in order to coordinate publication and release dates: https://forms.cloud.microsoft/e/eH4A8JVP6g

If you have any questions, please do not hesitate to contact the Editorial Office. Thank you for your contribution to The EMBO Journal.

Yours sincerely,

Cornelius Schneider, PhD
Editor
The EMBO Journal
c.schneider@embojournal.org

*****
Please note that it is The EMBO Journal policy for the transcript of the editorial process (containing referee reports and your response letters) to be published as an online supplement to each paper. If you should prefer removal of any referee-only figures included in the point-by-point response(s), e.g. because they may still be used for future publication or because they have been reproduced from published work by others, please do let us know immediately via response email.
More information is available here: https://link.springer.com/partners/embo-press/editorial-policies#Peer%20review

** Click here to be directed to your login page: https://emboj.msubmit.net

EMBO Press Author Checklist

Corresponding Author Name: Xuelei Lai, Lizhong Xiong
Journal Submitted to: The EMBO Journal
Manuscript Number: EMBOJ-2025-122526

USEFUL LINKS FOR COMPLETING THIS FORM
The EMBO Journal - Author Guidelines
EMBO Reports - Author Guidelines
Molecular Systems Biology - Author Guidelines
EMBO Molecular Medicine - Author Guidelines

Reporting Checklist for Life Science Articles (updated January
This checklist is adapted from Materials Design Analysis Reporting (MDAR) Checklist for Authors. MDAR establishes a minimum set of requirements in transparent reporting in the life sciences (see Statement of Task: 10.31222/osf.io/9sm4x). Please follow the journal's guidelines in preparing your
Please note that a copy of this checklist will be published alongside your article.

Abridged guidelines for figures
1. Data
The data shown in figures should satisfy the following conditions:
- ➞ the data were obtained and processed according to the field's best practice and are presented to reflect the results of the experiments in an accurate and unbiased manner.
- ➞ ideally, figure panels should include only measurements that are directly comparable to each other and obtained with the same assay.
- ➞ plots include clearly labeled error bars for independent experiments and sample sizes. Unless justified, error bars should not be shown for technical
- ➞ if n<5, the individual data points from each experiment should be plotted. Any statistical test employed should be justified.
- ➞ Source Data should be included to report the data underlying figures according to the guidelines set out in the authorship guidelines on Data

2. Captions
Each figure caption should contain the following information, for each panel where they are relevant:
- ➞ a specification of the experimental system investigated (eg cell line, species name).
- ➞ the assay(s) and method(s) used to carry out the reported observations and measurements.
- ➞ an explicit mention of the biological and chemical entity(ies) that are being measured.
- ➞ an explicit mention of the biological and chemical entity(ies) that are altered/varied/perturbed in a controlled manner.
- ➞ the exact sample size (n) for each experimental group/condition, given as a number, not a range;
- ➞ a description of the sample collection allowing the reader to understand whether the samples represent technical or biological replicates (including how many animals, litters, cultures, etc.).
- ➞ a statement of how many times the experiment shown was independently replicated in the laboratory.
- ➞ definitions of statistical methods and measures:
  - common tests, such as t-test (please specify whether paired vs. unpaired), simple χ2 tests, Wilcoxon and Mann-Whitney tests, can be unambiguously identified by name only, but more complex techniques should be described in the methods section;
  - are tests one-sided or two-sided?
  - are there adjustments for multiple comparisons?
  - exact statistical test results, e.g., P values = x but not P values < x;
  - definition of 'center values' as median or average;
  - definition of error bars as s.d. or s.e.m.

Please complete ALL of the questions below.
Select "Not Applicable" only when the requested information is not relevant for your study.

Materials

Newly Created Materials	Information included in the manuscript?	In which section is the information available? (Reagents and Tools Table, Materials and Methods, Figures, Data Availability Section)
New materials and reagents need to be available; do any restrictions apply?	Yes	Reagents and Tools Table

Antibodies	Information included in the manuscript?	In which section is the information available? (Reagents and Tools Table, Materials and Methods, Figures, Data Availability Section)
For **antibodies** provide the following information:<br>- Commercial antibodies: RRID (if possible) or supplier name, catalogue number and or/clone number<br>- Non-commercial: RRID or citation	Yes	Reagents and Tools Table

DNA and RNA sequences	Information included in the manuscript?	In which section is the information available? (Reagents and Tools Table, Materials and Methods, Figures, Data Availability Section)
**Short novel DNA or RNA including primers, probes**: provide the sequences.	Yes	Reagents and Tools Table

Cell materials	Information included in the manuscript?	In which section is the information available? (Reagents and Tools Table, Materials and Methods, Figures, Data Availability Section)
**Cell lines:** Provide species information, strain. Provide accession number in repository **OR** supplier name, catalog number, clone number, and/**OR** RRID.	Yes	Reagents and Tools Table
**Primary cultures:** Provide species, strain, sex of origin, genetic modification status.	Not Applicable	
Report if the cell lines were recently **authenticated** (e.g., by STR profiling) and tested for mycoplasma contamination.	Not Applicable	

Experimental animals	Information included in the manuscript?	In which section is the information available? (Reagents and Tools Table, Materials and Methods, Figures, Data Availability Section)
**Laboratory animals or Model organisms:** Provide species, strain, sex, age, genetic modification status. Provide accession number in repository **OR** supplier name, catalog number, clone number, **OR** RRID.	Not Applicable	
**Animal observed in or captured from the field:** Provide species, sex, and age where possible.	Not Applicable	
Please detail **housing and husbandry conditions**.	Not Applicable	

Plants and microbes	Information included in the manuscript?	In which section is the information available? (Reagents and Tools Table, Materials and Methods, Figures, Data Availability Section)
**Plants:** provide species and strain, ecotype and cultivar where relevant, unique accession number if available, and source (including location for collected wild specimens).	Yes	Reagents and Tools Table
**Microbes:** provide species and strain, unique accession number if available, and source.	Yes	Reagents and Tools Table

Human research participants	Information included in the manuscript?	In which section is the information available? (Reagents and Tools Table, Materials and Methods, Figures, Data Availability Section)
If collected and within the bounds of privacy constraints report on age, sex and gender or ethnicity for all study participants.	Not Applicable	

Core facilities	Information included in the manuscript?	In which section is the information available? (Reagents and Tools Table, Materials and Methods, Figures, Data Availability Section)
If your work benefited from core facilities, was their service mentioned in the acknowledgments section?	Not Applicable	

Design

Study protocol	Information included in the manuscript?	In which section is the information available? (Reagents and Tools Table, Materials and Methods, Figures, Data Availability Section)
If study protocol has been **pre-registered, provide DOI in the manuscript**. For clinical trials, provide the trial registration number **OR** cite DOI.	Not Applicable	
Report the **clinical trial registration number** (at ClinicalTrials.gov or equivalent), where applicable.	Not Applicable	

Laboratory protocol	Information included in the manuscript?	In which section is the information available? (Reagents and Tools Table, Materials and Methods, Figures, Data Availability Section)
Provide DOI OR other citation details if **external detailed step-by-step protocols** are available.	Not Applicable	

Experimental study design and statistics	Information included in the manuscript?	In which section is the information available? (Reagents and Tools Table, Materials and Methods, Figures, Data Availability Section)
Include a statement about **sample size** estimate even if no statistical methods were used.	Yes	Figures
Were any steps taken to minimize the effects of subjective bias when allocating animals/samples to treatment (e.g. **randomization procedure**)? If yes, have they been described?	Yes	Figures
Include a statement about **blinding** even if no blinding was done.	Not Applicable	
Describe **inclusion/exclusion criteria** if samples or animals were excluded from the analysis. Were the criteria pre-established?<br><br>If sample or data points were omitted from analysis, report if this was due to attrition or intentional exclusion and provide justification.	Not Applicable	
For every figure, are **statistical tests** justified as appropriate? Do the data meet the assumptions of the tests (e.g., normal distribution)? Describe any methods used to assess it. Is there an estimate of variation within each group of data? Is the variance similar between the groups that are being statistically compared?	Yes	Figures

Sample definition and in-laboratory replication	Information included in the manuscript?	In which section is the information available? (Reagents and Tools Table, Materials and Methods, Figures, Data Availability Section)
In the figure legends: state number of times the experiment was **replicated** in laboratory.	Yes	Figures
In the figure legends: define whether data describe **technical or biological replicates**.	Yes	Figures

Ethics

Ethics	Information included in the manuscript?	In which section is the information available? (Reagents and Tools Table, Materials and Methods, Figures, Data Availability Section)
Studies involving **human participants**: State details of **authority granting ethics approval** (IRB or equivalent committee(s), provide reference number for approval.	Not Applicable	
Studies involving **human participants**: Include a statement confirming that **informed consent** was obtained from all subjects and that the experiments conformed to the principles set out in the WMA Declaration of Helsinki and the Department of Health and Human Services Belmont Report.	Not Applicable	
Studies involving **human participants:** For publication of **patient photos**, include a statement confirming that consent to publish was obtained.	Not Applicable	
Studies involving experimental **animals**: State details of **authority granting ethics approval** (IRB or equivalent committee(s), provide reference number for approval. Include a statement of compliance with ethical regulations.	Not Applicable	
Studies involving **specimen and field samples:** State if relevant **permits** obtained, provide details of authority approving study; if none were required, explain why.	Not Applicable	

Dual Use Research of Concern (DURC)	Information included in the manuscript?	In which section is the information available? (Reagents and Tools Table, Materials and Methods, Figures, Data Availability Section)
Could your study fall under dual use research restrictions? Please check biosecurity documents and list of **select agents and toxins** (CDC): https://www.selectagents.gov/sat/list.htm	Not Applicable	
If you used a select agent, is the security level of the lab appropriate and reported in the manuscript?	Not Applicable	
If a study is subject to dual use research of concern regulations, is the name of the **authority granting approval and reference number** for the regulatory approval provided in the manuscript?	Not Applicable	

Reporting

The MDAR framework recommends adoption of discipline-specific guidelines, established and endorsed through community initiatives. Journals have their own policy about requiring specific guidelines and recommendations to complement MDAR.

Adherence to community standards	Information included in the manuscript?	In which section is the information available? (Reagents and Tools Table, Materials and Methods, Figures, Data Availability Section)
State if relevant guidelines or checklists (e.g., **ICMJE, MIBBI, ARRIVE, PRISMA**) have been followed or provided.	Not Applicable	
For **tumor marker prognostic studies,** we recommend that you follow the **REMARK** reporting guidelines (see link list at top right). See author guidelines, under 'Reporting Guidelines'. Please confirm you have followed these guidelines.	Not Applicable	
For **phase II and III randomized controlled trials**, please refer to the **CONSORT** flow diagram (see link list at top right) and submit the CONSORT checklist (see link list at top right) with your submission. See author guidelines, under 'Reporting Guidelines'. Please confirm you have submitted this list.	Not Applicable	

Data Availability

Data availability	Information included in the manuscript?	In which section is the information available? (Reagents and Tools Table, Materials and Methods, Figures, Data Availability Section)
Have **primary datasets** been deposited according to the journal's guidelines (see 'Data Deposition' section) and the respective accession numbers provided in the Data Availability Section?	Yes	Data Availability Section
Were **human clinical and genomic datasets** deposited in a public access-controlled repository in accordance to ethical obligations to the patients and to the applicable consent agreement?	Not Applicable	
Are **computational models** that are central and integral to a study available without restrictions in a machine-readable form? Were the relevant accession numbers or links provided?	Not Applicable	
If publicly available data were reused, provide the respective **data citations in the reference list**.	Not Applicable	
